# Identification of Glutathione S-Transferase Genes in Hami Melon (*Cucumis melo* var. *saccharinus*) and Their Expression Analysis Under Cold Stress

**DOI:** 10.3389/fpls.2021.672017

**Published:** 2021-06-08

**Authors:** Wen Song, Fake Zhou, Chunhui Shan, Qin Zhang, Ming Ning, Xiumin Liu, Xinxin Zhao, Wenchao Cai, Xinquan Yang, Guangfei Hao, Fengxian Tang

**Affiliations:** ^1^Engineering Research Center for Storage and Processing of Xinjiang Characteristic Fruit and Vegetables, Ministry of Education, College of Food, Shihezi University, Shihezi, China; ^2^College of Life Science and Food Engineering, Hebei University of Engineering, Handan, China

**Keywords:** Hami melon, cold stress, storage, glutathione S-transferases, genome-wide

## Abstract

As a group of multifunctional enzymes, glutathione S-transferases (GSTs) participate in oxidative stress resistance and cellular detoxification. Here, we identified 39 CmGST genes with typical binding sites from the Hami melon genome, and they can be classified into seven subfamilies. Their molecular information, chromosomal locations, phylogenetic relationships, synteny relationships, gene structures, protein–protein interactions, structure of 3-D models, and expression levels under cold stress were analyzed. Expression analysis indicates that cold-tolerant Jia Shi-310 (JS) had higher GST enzyme activities and expression levels of 28 stress-related genes under cold stress. Some CmGSTs belonging to Tau, Phi, and DHAR classes play significant roles under cold stress, and they could be regarded as candidate genes for further studies. The present study systematically investigated the characterization of the Hami melon GST gene family, extending our understanding of Hami melon GST mediated stress-response mechanisms in this worldwide fruit.

## Introduction

As a characteristic fruit of Xinjiang, China, Hami melon (*Cucumis melo* var. *saccharinus*) is economically and nutritiously significant. The latest data from the Food and Agriculture Organization of the United Nations (FAO^1^) shows that, in 2018, the production of Hami melon was 12.79 million tons in China. To preserve a high quality of nutrition and commercialization, the firm and ripe fruit are usually stored at low temperatures (0.5°C) in local areas. However, Hami melon is generally sensitive to low temperatures and long-term cold storage of Hami melon results in the chilling injury, known as peel pitting, softening, etc. Therefore, investigating the mechanism of postharvest Hami melon at low temperatures is a significant target in Hami melon storage programs.

Cold stress adversely affects the growth and development of the plant. Plant exposure to abiotic stresses, such as low temperature, results in the accumulation of reactive oxygen species (ROS), which, thus, causes oxidative stress (Ghosh et al., 2014). Singlet oxygen (^1^O_2_), superoxide anion (O_2_^–^), hydrogen peroxide (H_2_O_2_), and hydroxyl radical (HO⋅) produced by ROS are highly reactive and toxic and can cause the oxidative destruction of cells (Asada, 2006). To protect itself from damage under stress, plants may eliminate ROS through some antioxidant defense systems (Dionisio-Sese and Tobita, 1998; Moradi and Ismail, 2007; Montezano and Touyz, 2014). In plants, the antioxidant defense system includes non-enzymatic and enzymatic antioxidants. Non-enzymatic antioxidants contain ascorbate (AsA), glutathione (GSH), carotenoids, flavanones, tocopherols, anthocyanins, etc. (Nahar et al., 2015; Surowka et al., 2020). Enzymatic antioxidants contain superoxide dismutase (SOD), catalase (CAT), ascorbate peroxidase (APX), mono-dehydroascorbate reductase (MDHAR), dehydroascorbate reductase (DHAR), glutathione reductase (GR), glutathione peroxidase (GPX), glutathione S-transferase (GST), etc. (Venkateswarlu et al., 2012). Glutathione S-transferase belongs to the antioxidant enzyme family that plays a vital role in plant growth and development as well as stress management (Islam et al., 2019).

It is recognized that glutathione S-transferases could detoxify endobiotic and xenobiotic compounds by conjugating GSH to a hydrophobic substrate (Oztetik, 2008). This detoxification involves three phases: transformation, conjugation, and compartmentation (Light et al., 2005). A typical GST has two binding sites, the GSH binding site (G-site) in the N-terminal (GST-N) and the adjacent electrophilic substrate binding site (H-site) mainly formed by the C-terminal (GST-C). The GST-N is well conserved possibly due to its role in binding GSH, and GST-C is variable probably due to its combining multiple substances (Edwards and Dixon, 2005; Sylvestre-Gonon et al., 2019). The plant GST family is divided into seven classes, including Tau, Phi, Theta, Zeta, Lambda, glutathione-dependent dehydroascorbate reductase (DHAR), and tetrachlorohydroquinone dehalogenase (TCHQD) (Liu et al., 2013). Among these, Tau, Phi, Lambda, and DHAR classes are unique to the plant (Wang et al., 2013).

Numerous reports show that GSTs play vital roles in stress responses. Flax (*Linum usitatissimum*) GSTs are significant in the detoxification process of ROS and cell wall modification (Dmitriev et al., 2016). In the walnut tree (*Juglans regia*), JrGSTTau1 plays a positive role in osmotic tolerance and could be regulated by multiple upstream regulators (Yang et al., 2019). Jha et al. (2011) report that the expression of the tobacco GST gene is upregulated by different stresses, and overexpression of Tau class SbGST genes in transgenic tobacco show a better abiotic stress tolerance. Wang et al. (2013) find that MaGSTs play a key role in both development and abiotic stress responses in banana (*Musa acuminate L.* AAA group, cv. *Cavendish*). It is also reported that GSTs are key components in the metabolism of anthocyanins, flavonols, proanthocyanidins, cinnamic acid (Marrs et al., 1995; Liu et al., 2019), methyl jasmonate (Wagner et al., 2002), salicylic acid (Chen and Singh, 1999), auxin (Marrs, 1996; Chen and Singh, 1999), and ethylene (Zhou and Goldsbrough, 1993).

Based on our previous study, we find that GSTs are positively expressed in cold-tolerant Hami melon (JS) and are regarded as candidate cold-resistance proteins (Song et al., 2020). Herein, a comprehensive study was carried out to identify the Hami melon GST gene family on the aspect of bioinformatics analysis, including molecular information, distribution on chromosomes, phylogenetic relationships, synteny relationships, gene structures, protein–protein interactions (PPIs), 3-D models, functional annotation and expression levels of total enzyme activities, and genes in response to cold stress. Our results can provide new insights into the characteristics of GST gene family in Hami melon.

## Materials and Methods

### Identification of CmGST Genes

The whole Hami melon gene sequence (Melon (DHL92) genome 3.5.1) was obtained from the Cucurbit Genomics Database^2^. *Arabidopsis* GSTs (AtGSTs) gene sequences were downloaded from TAIR^3^. To get the Hami melon GSTs, we performed a BLASTP search against the *Arabidopsis* GSTs with a cutoff *E*-value (≤e^–3^). The identified Hami melon GST sequences were submitted to the NCBI Conserved Domain search tool (CD-search^4^) and Pfam^5^ to confirm the typical GST functional domain (Ding et al., 2017). The parameters used in the CD search were as follows: *E*-value, 0.01; the maximum number of hits, 500; and the result mode, concise. Furthermore, the ExPasy tool^6^ and Softberry^7^ were utilized to obtain molecular weights, isoelectric points, length of the sequence, and subcellular location of identified CmGSTs.

### Chromosomal Locations and Synteny Analysis of the CmGSTs

We performed multiple sequence alignments by MUSCLE. The phylogenetic trees were constructed using MEGA 7.0 by the neighbor-joining (NJ) method with 1000 bootstraps. Hami melon and other species (cucumber, zucchini squash, and watermelon) GST gene information was retrieved from the Cucurbit Genomics Database (see footnote 2). Chromosomal locations of CmGST genes were drawn using the MapChart 2.3 software program (Vijayakumar et al., 2016). Synteny analysis among Hami melon and other species was performed by the Multiple Collinearity Scan toolkit (MCScanX) and TBtools software v0.674 program^8^ (Chen et al., 2020). Then, the syntenic relationships were drawn using Circos (Krzywinski et al., 2009; Yan et al., 2019). The construction of PPI networks was conducted using the STRING database and Cytoscape 3.6.1 software program (Zhang et al., 2019). The substitution rate of non-synonymous (Ka) and synonymous (Ks) was calculated by KaKs Calculator 2.0 (Zhang et al., 2006). The divergence time of these CmGST paralogous pairs was calculated using the following formula (Baloglu et al., 2014):

T=Ks/2λ(λ=6.5×10e)-9.

### Structural Analysis of CmGST Genes

Exon-introns of CmGST genes were analyzed and presented by the Gene Structure Display Server 2.0^9^ (Gao et al., 2018). Conserved motifs were identified using the MEME Suite 5.1.1 program (Bailey et al., 2009; Cheng et al., 2018), and the parameters were set as follows: the maximum number of motifs: 18; motif site distribution: any number of repetitions (anr). The *cis*-elements of the coding region were analyzed by PlantCARE^10^ (Faraji et al., 2018).

### Prediction of 3-D Structure Models

The models of CmGST proteins were predicted by SWISS-MODEL^11^ (Biasini et al., 2014) and I-TASSER^12^ (Zhang, 2008). The validation of the predicted structure was performed according to the model evaluation score calculated by ProQ (Cristobal et al., 2001) and C-score (Roy et al., 2010; Yang et al., 2015). VMD software was utilized to visualize the 3-D models of CmGST proteins.

### Plant Materials and Stress Treatments

Two Hami melon species (*Cucumis melo* var. *saccharinus*), cold-tolerant Jia Shi-310 (JS) and cold-sensitive Golden Empress-308 (GE), were selected from No. 121 Regiment farm in Shihezi, Xinjiang, China. They were identified by the Processing and Storage of Fruit & Vegetables Institute, Shihezi University, Xinjiang, China. No other permissions were necessary to select the samples. After harvest, fruit of uniform size were stored in chambers at 0.5°C (±0.5°C) for 0 (control), 6, 12, 18, and 24 days. The fruit were divided randomly into three replicates per species, each consisting of six samples. The exocarp of each sample was collected and stored at –80°C for further analysis.

### Functional Analysis

GO enrichment analysis was performed using the TBtools software v0.674 program (see footnote 8) (Chen et al., 2020). KEGG^13^ was performed to identify the metabolic pathways (Kanehisa et al., 2008).

### Assay of Enzyme Activities, H_2_O_2_, and MDA Contents

ELISA kits (from Sino Best Biological Technology) were used to determine GST, GR activities. To assess GSH and glutathione oxidized (GSSG) contents, ELISA was performed according to its instruction.

H_2_O_2_ and MDA contents were determined according to Carvajal et al. (2018).

### Assay of Metabolite

Gas chromatography/mass spectrometry (GC/MS) was optimized for amino acids as previously described (Song et al., 2020). Raw data analysis was finished with Chroma TOF (V 4.3x,LECO) software, and the LECO-Fiehn Rtx5 database was utilized for metabolite identification by matching the mass spectrum and retention index (Shah et al., 2020). Six biological replicates were performed for each cultivar at each time point.

### Real-Time PCR Analysis

Real-time PCR was carried out according to Ning et al. (2019), and three biological replicates were used for each sample. A Hami melon GAPDH gene (LOC103484230), amplified with primers 5′-AAAGACTGGAGAGGTGGAAGAGC-3′ and 5′-TCAACGGTAGGAACACGGAAAGA-3′, was used as the internal reference gene. The relative expression level was calculated with the 2^–Δ^ ^Δ^ ^*Ct*^ method (Baloglu et al., 2014).

### Statistical Analysis

Data were presented as the mean ± SE (*n* = 3) and analyzed by IBM SPSS Statistics 25. The results were compared by Student’s *t*-test. Principal component analysis (PCA) was carried out with R software (version 3.6.1) (Cai W. et al., 2020; Cai W. C. et al., 2020). Pearson’s correlation coefficients (*r*) were visualized through Python 3. The scatterplot was visualized through imageGP^14^.

## Results

### Identification and Phylogenetic Analysis of CmGST Genes

In this work, a total of 39 CmGSTs were confirmed as Hami melon GSTs with a typical GST N-terminal or C-terminal domain, and the nomenclature followed the rules for their chromosomal position. The identified CmGST genes were distributed on 11 of 12 Hami melon chromosomes. The lengths of the CmGSTs were between 72 (CmGSTU24) and 301 amino acids (CmGSTU3). The theoretical pI values of CmGSTs were between 4.64 (CmGSTU24) and 10.13 (CmGSTU7), and the molecular weights of CmGSTs ranged from 8.08 kDa (CmGSTU24) to 35.61 kDa (CmGSTU3). The predicted subcellular location analysis revealed that 21 CmGSTs were located in cytoplasmic, 10 in nuclear, five in chloroplast, and one in membrane, respectively. We also identified two extracellular CmGSTs (CmGSTU3, CmGSTU7) (Supplementary Table 1).

**FIGURE 1:**
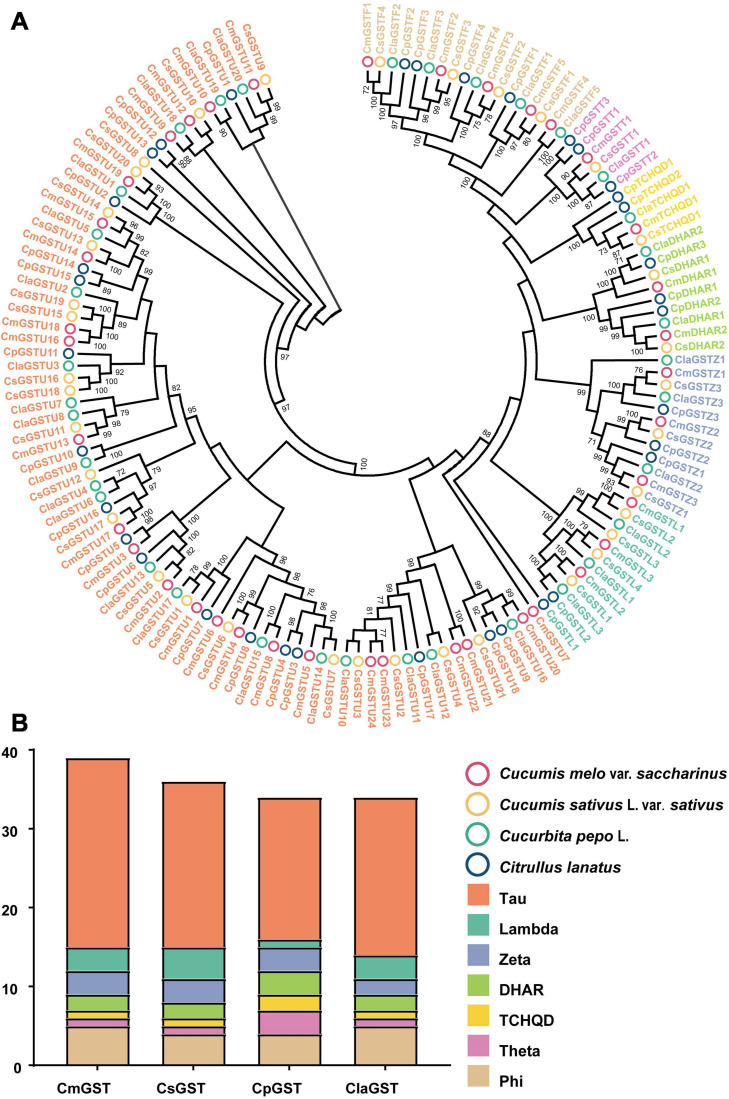
Phylogenetic analysis of GSTs from Hami melon, cucumber, zucchini squash, and watermelon. **(A)** A total of 145 GST proteins were divided into seven major subfamilies. **(B)** Comparison of GST members from Hami melon, cucumber, zucchini squash, and watermelon.

To classify the Hami melon GST proteins into subfamilies and identify the evolutionary relationships among Hami melon (*Cucumis melo* var. *saccharinus*), cucumber (*Cucumis sativus* L. var. *sativus*), zucchini squash (*Cucurbita pepo* L.), and watermelon (*Citrullus lanatus*), the sequences of the 39 CmGSTs, 36 CsGSTs, 35 CpGSTs, and 35 ClaGSTs, respectively, were utilized to construct a phylogenetic tree by the NJ method (Supplementary Table 2). The phylogram of 145 GST proteins from Hami melon and other species was divided into seven major subfamilies: Tau, Lambda, Phi, Zeta, TCHQD, DHAR, and Theta (Figure 1A). In Hami melon, a total of 24 CmGSTs were classified as the Tau subfamily, and five were attributed to the Phi clade. Three CmGSTs each were clustered into the Zeta and Lambda categories, respectively. Moreover, two CmGSTs were grouped into DHAR, and only one was categorized as the Theta and TCHQD subfamily, respectively (Figure 1B). The results indicate that the members of the Tau subfamilies occupied a prominent role in Hami melon GSTs. Intriguingly, GST genes in the same subfamily from different species were more similar than those of the same species but belonging to various subfamilies, which indicates a positive synteny between the same CmGSTs subfamily across distinct species (Zhao et al., 2018).

### Chromosomal Locations and Gene Duplications of CmGSTs

**FIGURE 2:**
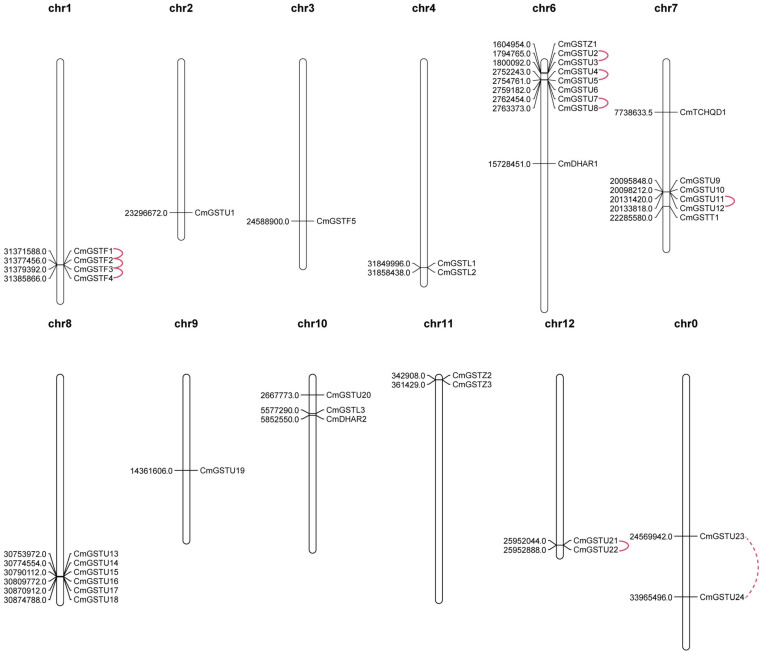
Chromosomal locations of CmGST genes. Tandem duplication gene pairs were linked by the solid line, and the segmental duplication gene pair was linked by the dashed line.

CmGSTs were widely distributed in Hami melon chromosomes. In our study, 37 of 39 CmGST genes were physically mapped in 11 chromosomes of Hami melon unevenly although only two genes (CmGSTU23, CmGSTU24) were mapped on unplaced scaffolds, and most of them were located on the proximate or the distal ends of the chromosomes (Figure 2). Chromosome 6 contained the largest number of CmGSTs (9), and chromosome 2/3/9 contained only one CmGST (Figure 2). No CmGST was mapped in chromosome 5. Comparing with other subfamilies, Tau members were diffusely located in seven chromosomes (chr2, 6, 7, 8, 9, 10, 12) (Figure 2).

Tandem and segmental duplication are regarded as the crucial genetic events to the generation of the gene family (Lynch and Conery, 2000; Cannon et al., 2004). A chromosomal region within 200 kb containing two or more genes is defined as a tandem duplication event (Holub, 2001). Segmental duplications multiply genes through polyploidy followed by chromosome rearrangements (Cannon et al., 2004; Yu et al., 2005). In the present work, eight out of nine CmGST gene pairs were tandem duplication although only one pair (CmGSTU23/24) was segmental duplication, indicating that both tandem and segmental duplication contributed to the expansion of this gene family in Hami melon, but the former played a pivotal driving force (Figure 2). Among tandem duplication, five pairs (five of eight, 62.5%) were in the Tau class, and three pairs (three of eight, 37.5%) were in the Phi class, indicating that the tandem duplication events had contributed more to Tau family expansion.

According to the ratio of non-synonymous to synonymous substitutions (Ka/Ks), we can better understand the Darwinian evolutionary selection of the CmGST gene family and measure the history of selection acting on coding sequences (Li et al., 1981; Chen et al., 2014; Cheng et al., 2018). In the present work, we found that six CmGST duplicated gene pairs had a Ka/Ks < 1, which suggests that these duplicated gene pairs are mainly under purifying selection. We also calculated the divergence time of these CmGST paralogous pairs, and the results reveal that the duplication events occurred approximately between 13,109,615 to 1,900,842 years ago (Table 1).

**TABLE 1:** Ka/Ks values and divergence time of six CmGST duplication gene pairs.

Duplicated Gene Pairs	Duplication Type	Ka	Ks	Ka_Ks	Type of Selection	Divergence Time (year)
CmGSTF1/2	Tandem	0.148401	0.865254	0.171512	Purify selection	6655800
CmGSTF2/3	Tandem	0.251933	1.70425	0.147826	Purify selection	13109615
CmGSTU2/3	Tandem	0.23079	0.660588	0.349371	Purify selection	5081446
CmGSTU4/5	Tandem	0.253447	0.772492	0.32809	Purify selection	5942247
CmGSTU11/12	Tandem	0.122053	0.780249	0.156429	Purify selection	6001914
CmGSTU21/22	Tandem	0.166094	0.247109	0.672146	Purify selection	1900842

### Synteny Analysis of GST Genes Among Hami Melon and Other Species

**FIGURE 3:**
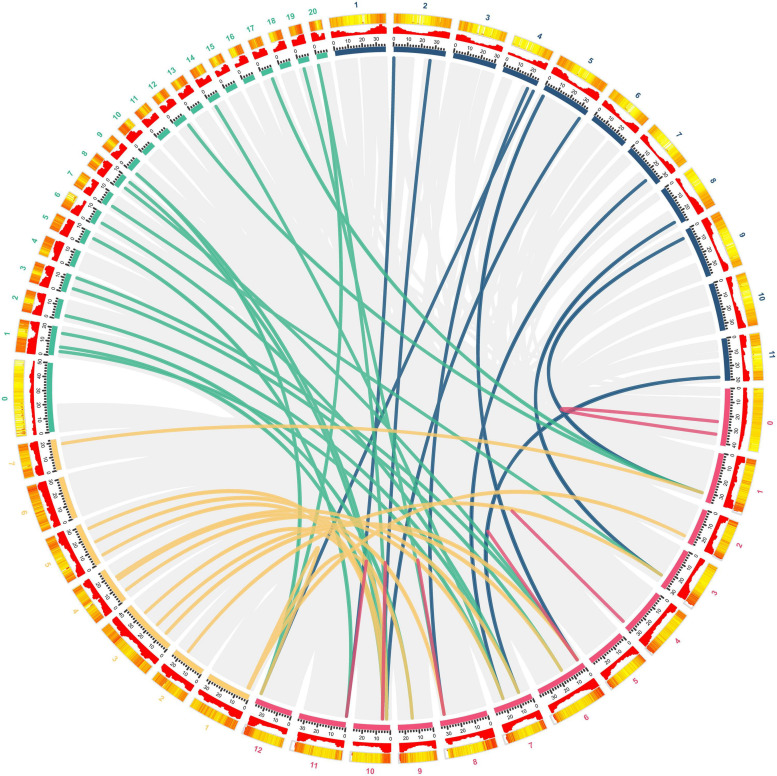
Syntenic analysis of GST genes from Hami melon, cucumber, zucchini squash, and watermelon. Red, yellow, green, and blue curves represent homologous gene pairs between Hami melon/Hami melon, Hami melon/cucumber, Hami melon/zucchini squash, and Hami melon/watermelon, respectively. Gray lines represent collinear gene pairs in Hami melon/Hami melon, Hami melon/watermelon, Hami melon/zucchini squash, and Hami melon/cucumber genomes.

To further explore the phylogenetic relationship of CmGST members, a synteny map of Hami melon (CmGSTs) with cucumber (CsGSTs), zucchini squash (CpGSTs), and watermelon (ClaGSTs) was constructed (Figure 3). A total of 19 CmGSTs presented a syntenic relationship with those in zucchini squash, followed by cucumber (16) and watermelon (11), indicating that CmGST genes were more closely related to those of zucchini squash (*Cucurbita pepo* L.) and cucumber (*Cucumis sativus* L. var. *sativus*) than watermelon (*Citrullus lanatus*) (Figure 3). Besides this, 10 CmGST collinear pairs were commonly identified between Hami melon and all the other three species, which implies that these gene pairs formed before the ancestral divergence (Xie et al., 2018). Of interest, we also identified six CmGSTs (CmGSTL3/CpGSTL1/2; CmGSTF1/CpGSTF2/3; CmGSTU9/CpGSTU1/12; CmGS TU20/CpGSTU9/17; CmGSTU21/CpGSTU9/17; CmTCHQD1/CpTCHQD1/2), and two CmGSTs (CmGSTL3/CsGSTL1/4; CmGSTU20/CsGSTU3/21) had two syntenic gene pairs between Hami melon/zucchini squash and Hami melon/cucumber, respectively. Half of these gene pairs were from the Tau subfamily. Thus, we conjecture that Tau members have played a major role during the evolution.

### Structural and Motif Analyses of CmGSTs

**FIGURE 4:**
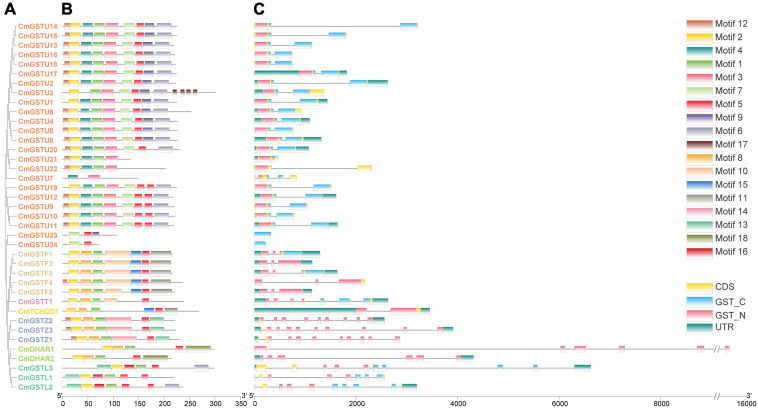
Phylogenetic relationships, conserved amino acid motifs, and gene structures of GSTs from Hami melon. **(A)** The phylogenetic tree of 39 Hami melon GST genes based on their deduced amino acid sequences. **(B)** Distributions of conserved amino acid motifs in CmGST genes. **(C)** Exon-intron organizations of CmGST genes.

To obtain more information about the structural characteristics of CmGSTs, we constructed a phylogenetic tree of all the Hami melon GST genes based on their deduced amino acid sequences. The conserved motifs, the typical GST domains and the exon-intron organizations were analyzed. The phylogenetic analysis showed that 39 CmGSTs belonging to the same subfamilies were clustered closely (Figure 4A). Moreover, most of the tandem duplicated genes were clustered closely, such as CmGSTU2/3, CmGSTU21/22, CmGSTF1/2, CmGSTL1/2, which confirmed the reliability of phylogenetic tree and synteny analysis.

To search the conserved amino acid motifs among CmGST proteins, the MEME web tool was utilized. A total of 18 distinct conserved motifs were identified (Figure 4B and Supplementary Figure 1). All members of CmGSTs contained motifs 1, 2, and 5. Besides this, motifs 3, 4, 6, 7, 9, and 17 were specifically observed in the Tau subfamily, and motif 14 was only present in the Zeta subfamily. Motif 18 was unique to DHAR members, and motif 16 was specific to the Lambda subfamily. Diverse motifs identified in Hami melon indicate the functional divergence of CmGSTs among different subfamilies. More interestingly, CmGST members within the same clade shared similar motifs, such as CmGSTU2/4/5/6/8/13/14/15/16/17/18, CmGSTF1/2/3/5, CmGSTZ1/2/3, and CmDHAR1/2, implying that the genes within the same subfamily are highly conserved, and their potential function might be similar.

As depicted in Figure 4C, CmGST genes possess 1 to 10 exons with a complete GST N-terminal or C-terminal domain and have a similar structure within the same subfamily. Except for CmGSTU7/21/23/24, the remaining 20 Tau members all contain two exons. All of the genes in the Phi clade process three exons, and seven and two exons were found in the Theta and TCHQD subfamilies, respectively. In Zeta members, CmGSTZ1 contains 10 exons, and CmGSTZ2/3 contains nine exons, respectively. All of the genes in the DHAR class process six exons. In the Lambda class, eight exons were present in CmGSTL1, and 10 were found in CmGSTL2/3, respectively. These results indicate that Phi and DHAR members are more conserved in Hami melon. Intriguingly, most of the tandem genes in the same group had the same numbers of exons, revealing the functional similarity among these genes.

### Prediction of PPI Networks and 3-D Structure Models of CmGST Proteins

**FIGURE 5:**
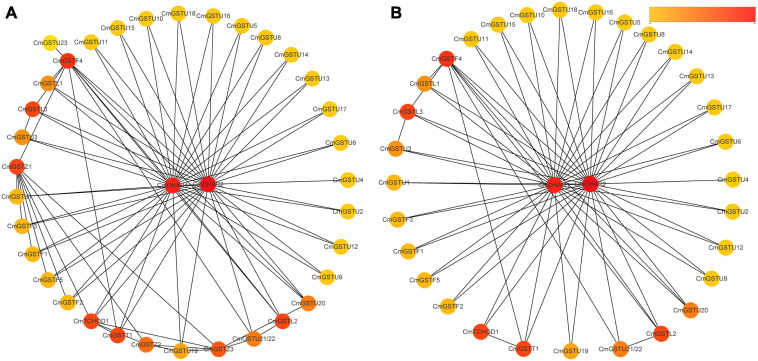
Protein–protein interactions networks of CmGST proteins. **(A)** PPI networks of 37 CmGST proteins. **(B)** PPI networks between two CmDHAR proteins and other members of CmGST proteins. Networks were ranked by the MCC method. The score was represented by a yellow-red gradient color: Yellow nodes indicate a low score, and red nodes indicate a high score.

Protein–protein interactions regulate approximately all cellular activities as well as adjust metabolic pathways in plants (Faraji et al., 2018). To further investigate the functions of CmGSTs, an interaction network among different members of CmGST proteins was constructed. As shown in Figure 5A, a high degree of interaction was exhibited between CmDHARs and other members of CmGSTs. Among these CmGSTs, Tau members occupied a large number (21), followed by Phi members (5), Lambda members (3), Theta (1), and TCHQD (1) (Figure 5B and Supplementary Table 3). Therefore, as the most linked proteins, we speculated that CmDHARs played a predominant role in the regulation mechanisms among CmGSTs in Hami melon.

**FIGURE 6:**
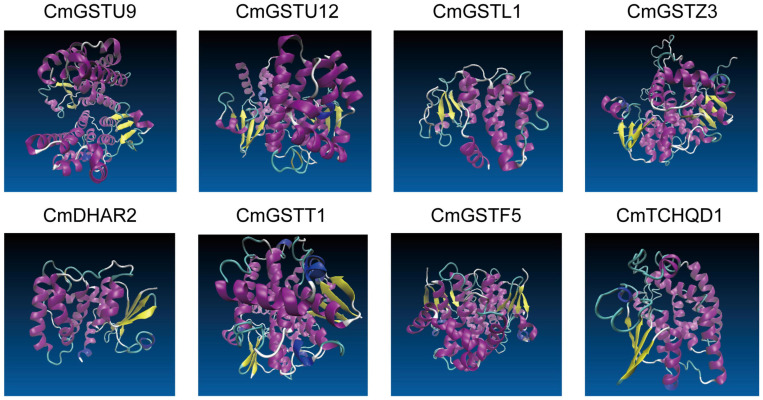
The 3-D models of eight CmGST proteins. Different colors show the types of structures: α helices in purple, β sheets in yellow, turn in cyan, and random coil in white.

Eight CmGST protein models with validation are presented in Figure 6. Except for CmTCHQD1, the protein models of the other seven CmGST members were predicted by the homology modeling method using SWISS-MODEL. The protein structure of these seven CmGSTs was modeled at >30% sequence identity (Bienert et al., 2017; Waterhouse et al., 2018; Studer et al., 2020), and the ProQ results show a >4 LG score and >0.1 Max Sub, indicating the high reliability of the model prediction (Supplementary Table 4 and Supplementary Figure 2). The model of CmTCHQD1 was constructed by I-TASSER with a *C*-score of –0.93, revealing that the predicted model was of good quality (Roy et al., 2010; Yang et al., 2015). The 3-D models of all these eight CmGSTs chiefly contain α helix, β sheet, turn, and random coil (Figure 6). The helices observed in the C-terminal domain contribute to the formation of the H-site and, therefore, to the xenobiotic substrate specificity (Pouliou et al., 2017). Intriguingly, the C-terminal domain was composed mainly of α helices, and the N-terminal domain was composed of α helices and β sheets. The present work offers a preliminary basis for understanding the structure of CmGST proteins, and the relationship between protein structure and molecular function will be discussed in further studies.

### Expression Profiles of Stress-Related CmGSTs Under Cold Treatment

Regulation of gene expression at the promoter level is mainly controlled by the *cis*-elements localized upstream of the transcriptional start site (Hernandez-Garcia and Finer, 2014). To understand the transcriptional regulation mechanisms of CmGST members under abiotic stress, CmGST genes located in 1000-bp DNA sequence upstream were selected to observe the predicted *cis*-elements via PlantCare. According to their predicted functions, we identified 45 promoter cis-elements that were clustered into five clades: light-responsive, hormone-responsive, development-related, abiotic stress-response, and other elements (Supplementary Tables 5,6). As clade one, the light-responsive elements were composed of G-box, AE-box, GT1-motif, Sp1, ATCT-motif, Box 4, I-box, MRE, GATA-motif, TCCC-motif, TCT-motif, LAMP-element, Gap-box, 3-AF1 binding site, and GTGGC-motif, among which G-box (17) was the most abundant (Zhu et al., 2014). Another clade of *cis*-elements was hormone-responsive elements, which included P-box, TCA-element, TGA-element, TATC-box, SARE, ERE, TGACG-motif, CGTCA-motif, and ABRE. The cis-element ABRE involved in the abscisic acid responsiveness appeared to be the most abundant hormone-related element (20), followed by TGACG-motif (14) and CGTCA-motif (14), which were involved in the MeJA-responsiveness. Clade three was development-related elements, mainly including GCN4-motif, circadian, CAT-box, CCGTCC-box, MSA-like, DOCT, HD-Zip 1, and O2-site, of which zein metabolism regulation related O2-site was the most abundant development-related element (10). The fourth clade was associated with abiotic stress-response elements, comprising LTR, WUN-motif, GC-motif, ARE, MBS, W box, and TC-rich repeats. Among these elements, ARE, essential for the anaerobic induction, was the most abundant (20). Clade five was clustered in other elements, including TCA, STRE, and MYC, etc. (Supplementary Tables 5, 6).

**FIGURE 7:**
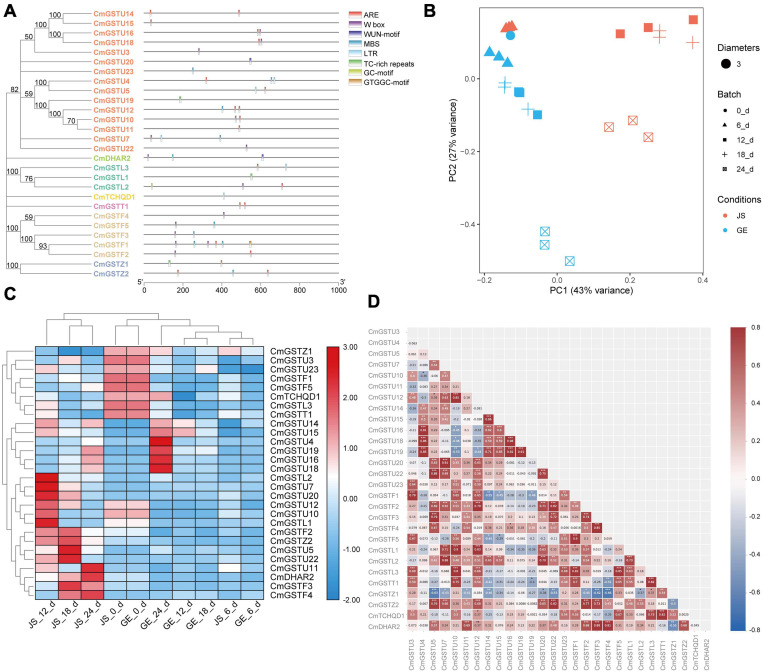
Stress-related CmGSTs in JS and GE of cold treatment. **(A)** Stress-related cis-elements in CmGSTs promoters. **(B)** Principal component analysis of stress-related CmGSTs from JS and GE after cold treatment. **(C)** Hierarchical clustering analysis of stress-related CmGSTs from JS and GE. **(D)** Correlation analysis of 28 stress-related CmGSTs.

**FIGURE 8:**
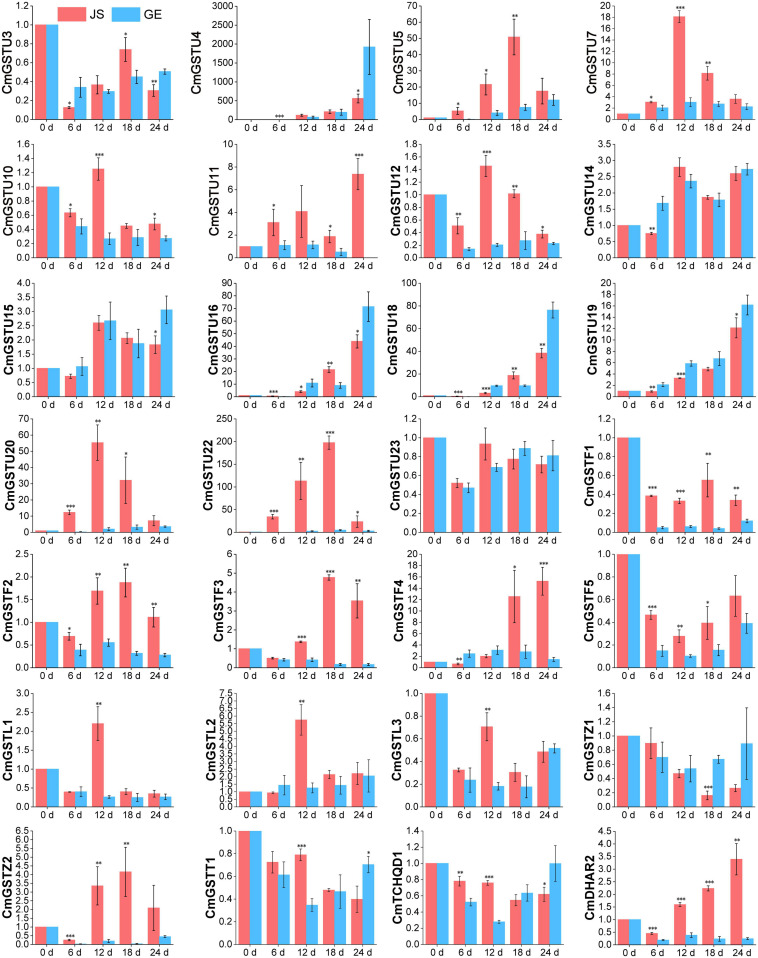
Expression patterns of 28 stress-related CmGSTs in JS and GE under cold treatment as revealed by q-PCR. Values are means ± standard error of three replicates. The error bars represent standard error of the means. The asterisk indicates significant difference (*P < 0.05, **P < 0.01, ***P < 0.001) among cold treatment for the same sampling day.

Moreover, 28 of 39 (71.8%) CmGSTs contained at least one stress-related element, which indicates that most CmGSTs could respond to environmental stresses (Figure 7A). Tau possesses a top number of stress-related elements (26), followed by Phi (12), Lambda (6), Zeta (5), DHAR (3), Theta (2), and TCHQD (1). Quantitative RT-PCR was applied to investigate the expression profiles of each CmGST (Supplementary Table 7). Based on the expression profiles of 28 stress-related CmGSTs, PCA was performed (Figure 7B). PCA analysis revealed that 70% of the overall variance was accounted for the first two principal components. After 6 days of cold treatment, the changes of 28 stress-related CmGSTs in JS and GE clustered away from each other, indicating the different expression patterns of CmGSTs in JS and GE after a long period of cold treatment. Hierarchical clustering expression analysis showed that many more CmGSTs were upregulated in JS during cold treatment (Figure 7C). Moreover, most of these stress-related CmGSTs were positively correlated (Figure 7D). Approximately 61% of these CmGSTs were continually upregulated, and nine CmGSTs (CmGSTU4/5/7/16/18/19/20/22 and CmGSTF4) were highly expressed in both JS and GE during long-term cold treatment (Figure 8). Of note, 12 CmGSTs (CmGSTU5/7/11/12/20/22, CmGSTF2/3/4, CmGSTL2, CmGSTZ2, and CmDHAR2) were significantly highly expressed in JS. We also found that 18 (64.29%) CmGSTs were significantly upregulated in JS at 12 days of storage. The results suggest that these stress-related CmGSTs might play crucial roles in response to stress (Islam et al., 2019).

### Functional Annotation of CmGSTs

**FIGURE 9:**
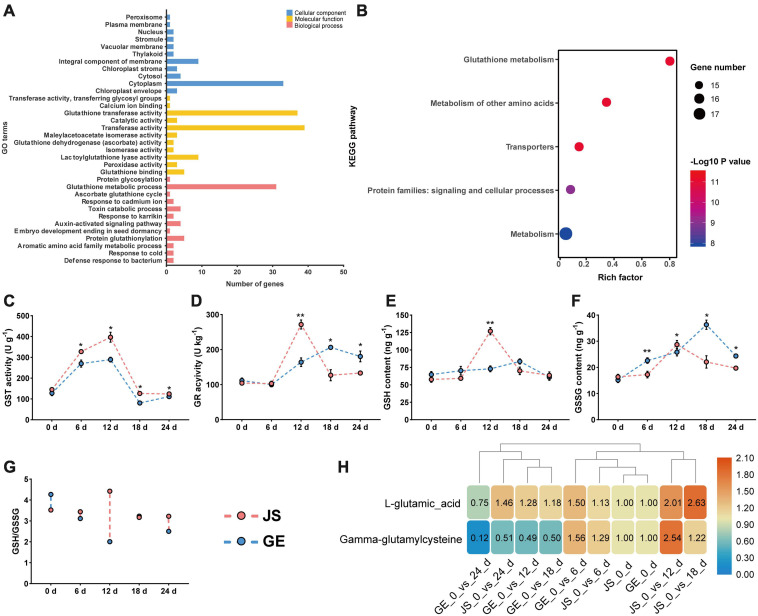
Functional annotation of CmGSTs and detection of enzyme activities. **(A)** GO function annotation of 39 CmGSTs. **(B)** KEGG enrichment scatterplot of 28 stress-related CmGSTs. Activities of **(C)** GST and **(D)** GR, contents of **(E)** GSH and **(F)** GSSG, ratio of **(G)** GSH/GSSG, and contents of **(H)** L-glutamic acid and gamma-glutamylcysteine of JS and GE under cold stress during storage. Values are means ± standard error of three replicates. The error bars represent standard error of the means. The asterisk indicates significant difference (*P < 0.05, **P < 0.01) among cold treatment for the same sampling day.

In Figure 9A, 39 CmGST genes were grouped into 34 GO terms, including 11 cellular component (CC), 11 molecular functions (MF), and 12 biological processes (BP). “Cytoplasm” (53.2%) and “integral component of membrane” (14.5%) were the top two highly represented terms in CC and “transferase activity” (37.1%) and “glutathione transferase activity” (35.2%) in MF. “Glutathione metabolic process” (54.4%) was the most highly represented term in BP. Further, KEGG enrichment analysis of 28 stress-related CmGSTs revealed that a total of 18 CmGSTs were predicted to be involved in five pathways, among which “glutathione metabolism” was the most enriched pathway (Figure 9B and Supplementary Table 8).

**FIGURE 10:**
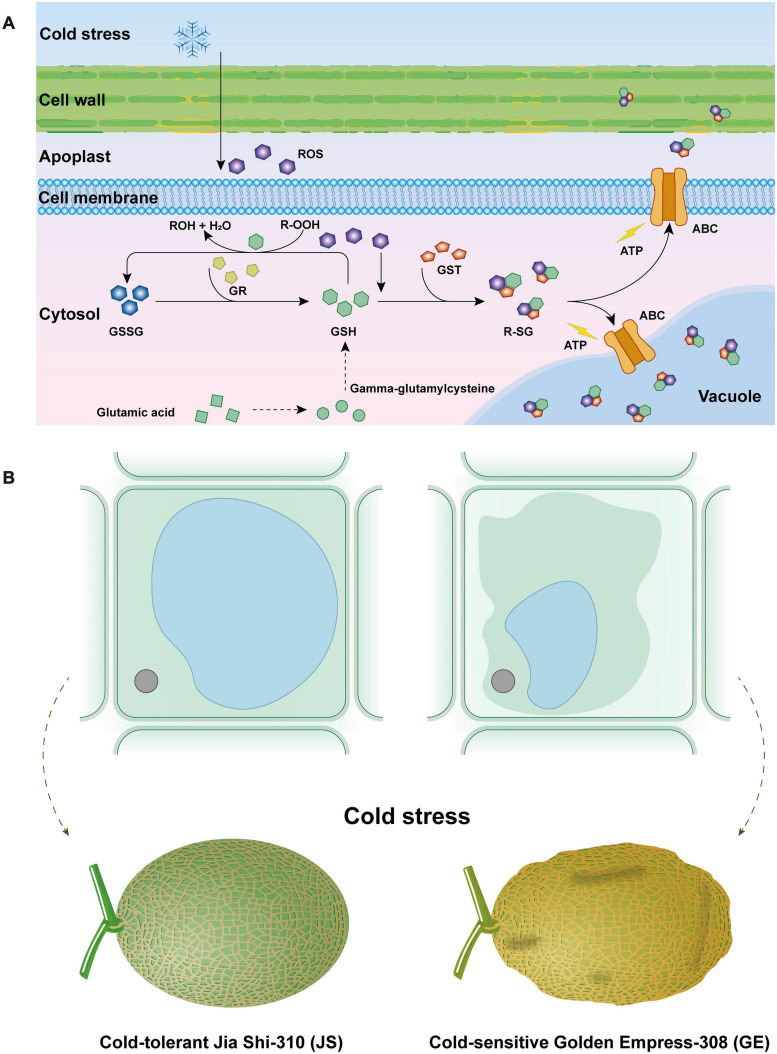
Effect of cantaloupe “glutathione metabolism” process in response to cold stress **(A)** and osmotic stress caused by cold stress to cantaloupe **(B)**.

Based on the KEGG enrichment analysis, we found that the “glutathione metabolism” pathway was crucial in the process of detoxification reactions (Figure 10A). Accordingly, enzyme activities of GST and GR and contents of GSH and GSSG were detected then. The GST activities peaked at 12 days in both JS and GE (Figure 9C). However, they were higher in JS than in GE during the whole storage period (Figure 9C). The GR activities reached a peak on 12 days of storage and decreased dramatically afterward in JS although the peaking time was delayed up to 18 days in GE, and the contents of GSH showed a similar tendency (Figures 9D,E). A sign of stress, GSSG contents were higher in GE than in JS (Figure 9F). JS showed maximum GSSG contents on 12 days of storage, and the GSSG content peaked at 18 days in GE (Figure 9F). Compared with cold-sensitive GE, cold-tolerant JS revealed a higher level of GST and GR activities on 12 days of storage. In Figure 9G, the higher ratio of GSH/GSSG in JS showed better cold stress–tolerance capacities, which was in accordance with previous findings (Hasanuzzaman et al., 2011; Alam et al., 2014). Moreover, changes of L-glutamic acid and gamma-glutamylcysteine reveal that the upregulation of glutathione biosynthesis-related amino acids was crucial for cold resistance (Figure 9H and Supplementary Table 9). The ROS levels of JS and GE at different storage periods were counted, including the H_2_O_2_ and MDA contents (Table 2). After cold treatment, the ROS levels increased and then decreased in Hami melons. The H_2_O_2_ contents peaked at 12 days in both JS and GE but were significantly higher in GE. Compared with JS, the MDA contents were significantly higher in GE at 12 and 18 days of storage. Taken together, we found that higher transcript and protein activities of the GSTs contributed to the cold tolerance of Hami melon.

**TABLE 2:** Reactive oxygen species levels in JS and GE at different storage periods^*a*^.

ROS levels	JS					GE				
	**0 day**	**6 days**	**12 days**	**18 days**	**24 days**	**0 day**	**6 days**	**12 days**	**18 days**	**24 days**
H_2_O_2_ (μmol g^–1^)	34.56 ± 0.02	32.37 ± 0.93	41.72 ± 2.38**	41.40 ± 1.06	30.11 ± 1.19	34.52 ± 1.6	30.54 ± 0.8	48.61 ± 0.76	39.7 ± 1.34	28.71 ± 0.26
MDA (nmol g^–1^)	6.53 ± 0.24*	9.35 ± 0.95	10.66 ± 0.92**	14.28 ± 2.16*	9.16 ± 0.7	7.91 ± 0.88	8.44 ± 0.03	15.48 ± 0.84	8.6 ± 0.95	8.6 ± 0.7

^*a*^Values are means ± standard error of three replicates. The asterisk indicates significant difference (*P < 0.05, **P < 0.01) among cold treatment for the same sampling day.

## Discussion

### Identification of Hami Melon GSTs

Glutathione S-transferases (GSTs; EC. 2.5.1.18) are a family of ubiquitous enzymes that are involved in cellular detoxification by detoxifying a diverse class of exogenous and endogenous electrophilic substrates (Simarani et al., 2016; Shehu et al., 2019). Plant GSTs showed a critical role in improving abiotic stress resistance. In rice, 79 GST genes were identified, and many of the GST genes were commonly regulated during various abiotic (20), arsenate (32), and biotic stress (48) conditions (Jain et al., 2010). Pouliou et al. (2017) find that GmGSTU5-5 and GmGSTU8-8 exhibited high activities as glutathione peroxidases, capable of reducing toxic hydroperoxides in *Glycine max*. Wang et al. (2019) report that 14 of 330 TaGST genes could respond to different abiotic stresses and hormones, especially salt stress and abscisic acid in wheat. In *Brassica oleracea*, Vijayakumar et al. (2016) find 65 *Brassica oleracea* glutathione transferases (BoGST), most of which were highly expressed at 1 and 6 h in the cold-susceptible (CS) and cold-tolerant (CT) lines, respectively, and three BoGSTs (BoGSTU10/19/24) were regarded as candidate genes in resisting stress. In the present work, a total of 39 CmGST members were identified and divided into seven clades, among which Tau (24) was the largest clade. CmGST genes were widely distributed on 11 of 12 Hami melon chromosomes. The subcellular location analysis indicated that 21 of 39 (53.8%) were located in cytoplasm, revealing that GSTs were soluble (Oztetik, 2008). Except for two extracellular CmGSTs (CmGSTU3, CmGSTU7), the rest of the CmGSTs were located in nuclear (10), chloroplast (5), and membrane (1), respectively. The chromosome and subcellular location analysis indicates that the wide distribution of CmGSTs in Hami melon leads to the diversity and complexity of this gene family, which could be one key factor for their roles in the process of catalysis and detoxification (Kayum et al., 2018).

Phylogenetic tree analysis reveals that the CmGST members were more closely related to those in the same clade from different species than to the other CmGSTs from the same species, implying that higher synteny might exist in these GST proteins (Zhao et al., 2018). More interestingly, we found that the most adjacent CmGST genes clustered together into the phylogenetic tree were on the same chromosome, which reveals that members within the same subfamily might have common evolutionary origins and a similar pathway or biological process (Zhang et al., 2018).

### Evolution and Gene Structure of Hami Melon GSTs

The expansions of gene families and genome evolutionary mechanisms mainly depend on gene duplication events (Vision et al., 2000). The major duplication patterns are tandem and segmental duplication (Kong et al., 2007). In the present study, tandem duplication played a predominant driving force in the expansion of the CmGST gene family. We find that tandem duplication events contributed more to Tau clade expansion. Probably due to the roles of detoxification and defending responses, the large-scale expansion within the Tau clade CmGSTs could enhance tolerance of plants to various environmental stresses (He et al., 2016).

It is reported that the exon-intron structure is significant in the evolution of genes (Xu et al., 2012). In Hami melon, CmGST genes in the same clade share similar exon-intron structure, demonstrating high conservation in each subfamily, particularly in the Phi and DHAR subfamilies. Jeffares et al. (2008) report that a compact gene structure with fewer introns could respond in a timely manner to stress. Thus, we inferred that Tau, Phi, and TCHQD members with fewer introns could rapidly respond to stress. The conserved motifs analysis suggests that the CmGSTs within the same subfamily were highly conserved, and their potential function might be similar. Taken together, the characteristics of the exon-intron structure and putative motifs were well conserved in recent subfamilies.

### CmGSTs Gene Expression Profile Under Cold Stress

*Cis*-elements could control or regulate the expression of genes, thus modulating plant response against stress and developmental changes (Narusaka et al., 2003). The current work detected 45 promoter *cis*-elements, and they could be clustered into five groups: light-responsive, hormone-responsive, development-related, abiotic stress-response, and other elements, showing that various kinds of *cis*-elements exist in the promoters of CmGST genes. All these *cis*-elements might work synergistically depending on the type of diverse functions to confer CmGSTs a potential function in response to various stimuli. Furthermore, 28 CmGSTs with at least one stress-associated element were selected for q-PCR analysis. The results show that most of CmGST genes were positively correlated and upregulated in cold-tolerant JS than in cold-sensitive GE, revealing that CmGSTs are essential factors in Hami melon tolerance mechanism under cold stresses.

### Functional Analysis of Hami Melon GSTs

**FIGURE 11:**
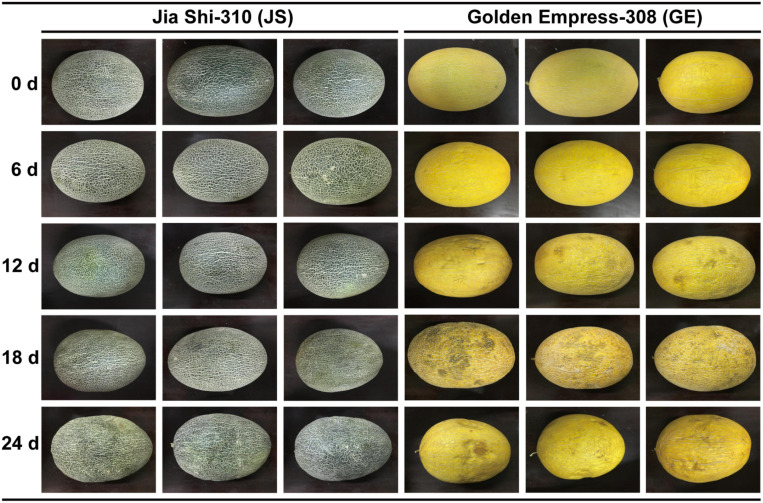
Different cold tolerance between “Jia Shi-310” (JS) and “Golden Empress-308” (GE), and experimental design.

GO and KEGG functional analysis indicate that the “glutathione metabolism” pathway plays a significant role in detoxification reactions (Figure 10A). Under cold stress, part of glutathione (GSH) is oxidized by ROS (e.g., H_2_O_2_) as the form of GSSG. Besides this, a critical component in the antioxidant system, GSH could also transform R-OOH to R-OH and H_2_O. When the GSH decreased, glutamic acid and gamma-glutamylcysteine biosynthesis pathways were activated to increase its content. Also, the glutathione reductase (GR) activity was increased, which could transform GSSG to GSH and keep it more reduced. Then, the GSH conjugates so formed are rendered less reactive and more water-soluble, thus facilitating their eventual elimination (Oztetik, 2008; Rahantaniaina et al., 2017). Generally, GST reactions with xenobiotics result in the formation of an *S*-glutathionylated (R-SG) reaction product as a consequence of the conjugation of the toxic substrate (Oztetik, 2008). Then, ATP-binding cassette transporter (ABC) can transfer these conjugates across membranes, and they are either sequestered in the vacuole for further processing or transferred to the apoplast for deposition into lignin or other cell wall components (Sandermann, 1992; Marrs, 1996; Dixon et al., 1998; Edwards and Dixon, 2004). Hence, GSTs are usually detoxification reactions. Adverse environmental stresses such as cold stress could lead to water deficit, which can generate osmotic stress (OS) to plant cells (Zhu, 2016; Lozano-Juste et al., 2020). Higher accumulation of ROS could result in the disorder and severe OS in plant cells (Figure 10B). When water leaves plant cells by osmosis, the cell membrane and its contents shrink away from the rigid cell wall, and turgor pressure decreases, which is called plasmolysis, causing Hami melon peel pitting and softening (Figures 10B, 11). In the present work, we find that compared with cold-sensitive GE, cold-tolerant JS revealed a higher level of GST and GR activities on 12 days of storage. Accordingly, we assume that the higher expression levels of GST genes and enzyme activities could scavenge the accumulation of ROS and, thus, reduce the osmotic stress, maintaining a higher level of cold tolerance.

## Conclusion

A comprehensive analysis of the Hami melon GST gene family was performed in this work. A total of 39 CmGST genes were confirmed. Then, we analyzed their molecular information, chromosomal locations, phylogenetic relationships, synteny relationships, gene structures, 3-D models, PPIs, functional annotation, total enzyme activities, and gene expression levels under cold stress. Compared with cold-sensitive GE, total GST activities and most of the stress-related CmGST genes were upregulated in cold-tolerant JS, indicating that CmGSTs were significant in resisting the cold stress. Our findings identified the characteristics of Hami melon GST genes and could provide new clues to better understand the relationship between the CmGST genes and cold-tolerance mechanisms of Hami melon.

## Data Availability Statement

The original contributions presented in the study are included in the article/Supplementary Material, further inquiries can be directed to the corresponding author/s.

## Author Contributions

CS, FT, and XY designed the experiments. QZ, XL, and FZ performed the experiments. WC, XZ, and WS performed the HCL and PCA analysis. WS, MN, and GH analyzed the data. WS wrote the manuscript. All authors approved the final version of manuscript.

## Conflict of Interest

The authors declare that the research was conducted in the absence of any commercial or financial relationships that could be construed as a potential conflict of interest.

## Footnotes

1 http://www.fao.org/worldfoodsituation/csdb/en/

2 http://cucurbitgenomics.org

3 https://www.arabidopsis.org/

4 https://www.ncbi.nlm.nih.gov/Structure/cdd/wrpsb.cgi

5 http://pfam.xfam.org/search#tabview=tab1

6 http://web.expasy.org/protparam/

7 www.softberry.com

8 https://github.com/CJ-Chen/TBtools

9 http://gsds.cbi.pku.edu.cn/

10 http://bioinformatics.psb.ugent.be/webtools/plantcare/html/

11 https://swissmodel.expasy.org/interactive

12 https://zhanglab.ccmb.med.umich.edu/I-TASSER/

13 http://www.genome.jp/kegg/

14 http://www.ehbio.com/ImageGP

## Supplementary Material

Supplementary Figure 1 Putative motifs predicted in Hami melon GSTs.

Supplementary Figure 2 Ramachandran plot analyses of 3-D model of eight CmGST proteins.

## Abbreviations

JS: Jia Shi-310
GE: Golden Empress-308
GST: glutathione S-transferase
PPI: protein–protein interactions
ROS: reactive oxygen species
H_2_O_2_: hydrogen peroxide
MDA: malondialdehyde
GSSG: glutathione oxidized
GR: glutathione reductase
GSH: glutathione
DHAR: dehydroascorbate reductase
TCHQD: tetrachlorohydroquinone dehalogenase.
