# Supplementary figures and images for: Identification of Glutathione S-Transferase Genes in Hami Melon (Cucumis melo var. saccharinus) and Their Expression Analysis Under Cold Stress
Source: Front Plant Sci. 2021 Jun 8;12:672017. doi: 10.3389/fpls.2021.672017 (PMC8217883; doi:10.3389/fpls.2021.672017)

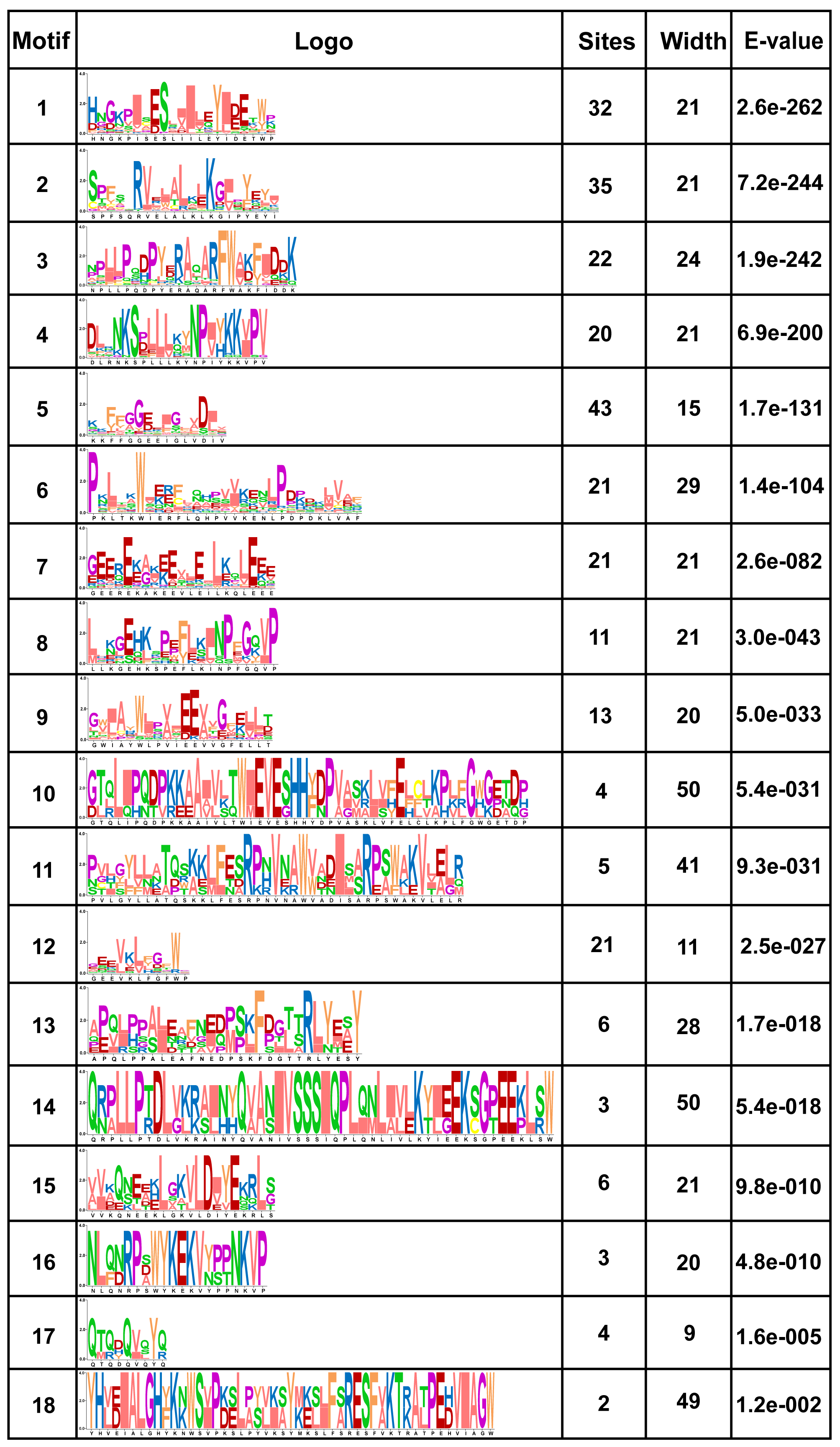

Supplement: Supplementary Figure 1 — Putative motifs predicted in Hami melon GSTs. [file Image_1.TIF]

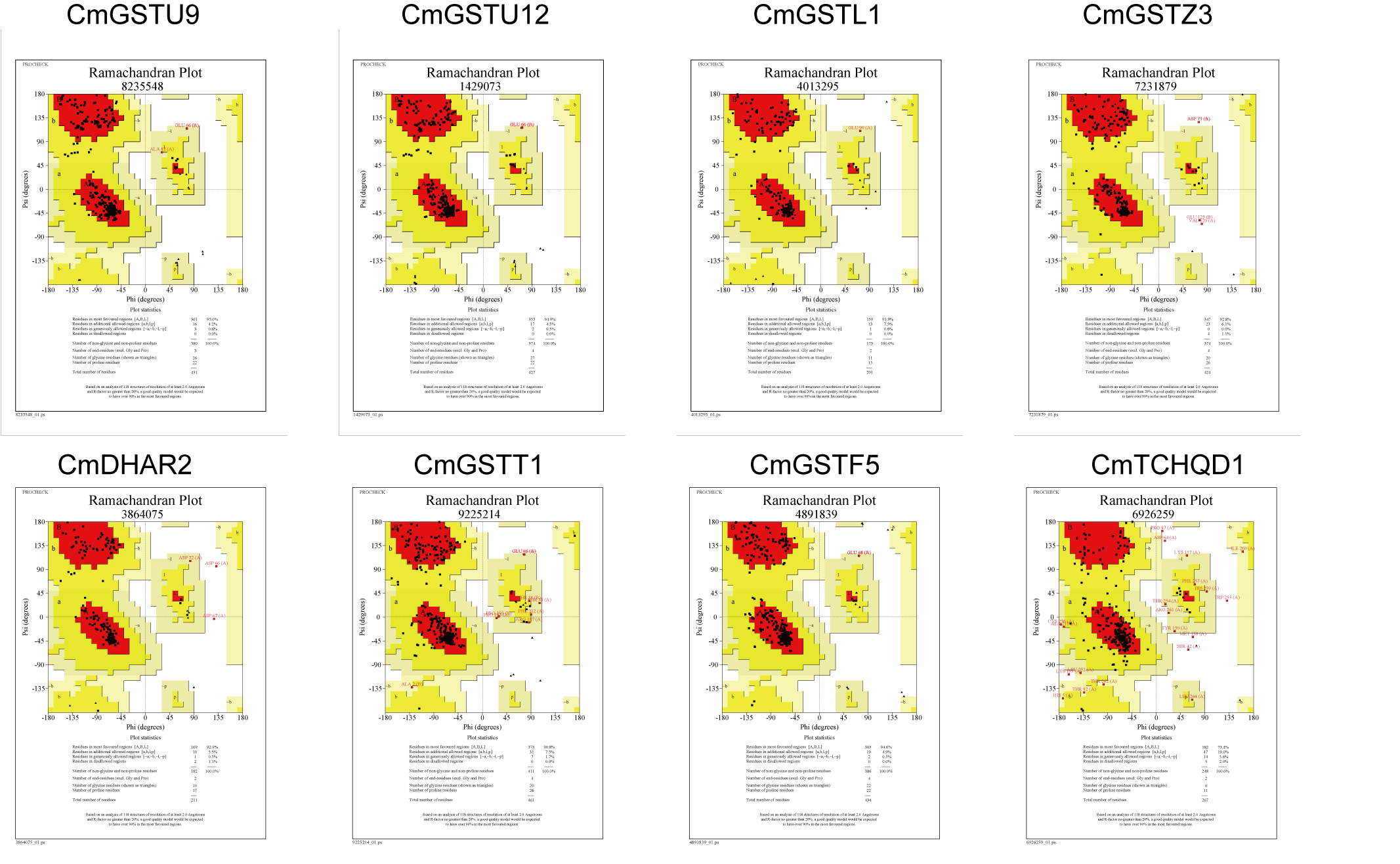

Supplement: Supplementary Figure 2 — Ramachandran plot analyses of 3-D model of eight CmGST proteins. [file Image_2.TIF]
