# Supplementary material for: Identification of Glutathione S-Transferase Genes in Hami Melon (Cucumis melo var. saccharinus) and Their Expression Analysis Under Cold Stress
Source: Front Plant Sci. 2021 Jun 8;12:672017. doi: 10.3389/fpls.2021.672017 (PMC8217883; doi:10.3389/fpls.2021.672017)
Supplement: Supplementary file 3 [file Data_Sheet_1.doc]

**Table S1**

Description of the 39 Hami melon glutathione S-transferase (GST) genes.

| **Gene Name** | **Gene ID** | **Exon-Intro** | **Motif** | **Chromosome** | **Start Pos** | **End Pos** | **Strand** | **Length** | **Theoretical pI** | **Molecular weight (average)** | **Location** |
| --- | --- | --- | --- | --- | --- | --- | --- | --- | --- | --- | --- |
| CmGSTF1 | MELO3C016031T1 | 3 | 7 | Chr1 | 31371587 | 31372877 | - | 216 | 6.24 | 23832.4 | Cytoplasmic |
| CmGSTF2 | MELO3C016032T1 | 3 | 7 | Chr1 | 31377455 | 31378591 | + | 215 | 6.07 | 24016.71 | Cytoplasmic |
| CmGSTF3 | MELO3C016033T1 | 3 | 7 | Chr1 | 31379391 | 31381019 | - | 217 | 6.23 | 23985.36 | Cytoplasmic |
| CmGSTF4 | MELO3C016034T1 | 3 | 7 | Chr1 | 31385866 | 31388038 | - | 237 | 6.69 | 27250.52 | Cytoplasmic |
| CmGSTU1 | MELO3C017341T1 | 2 | 8 | Chr2 | 23296672 | 23298107 | + | 225 | 5.4 | 25660.59 | Nuclear |
| CmGSTF5 | MELO3C011334T1 | 3 | 7 | Chr3 | 24588900 | 24590027 | + | 221 | 5.89 | 24959.36 | Cytoplasmic |
| CmGSTL1 | MELO3C009300T3 | 8 | 9 | Chr4 | 31849995 | 31852556 | - | 220 | 4.89 | 24930.55 | Chloroplast |
| CmGSTL2 | MELO3C009299T2 | 10 | 9 | Chr4 | 31858440 | 31861638 | - | 238 | 5.23 | 27202.95 | Chloroplast |
| CmGSTZ1 | MELO3C006189T1 | 10 | 6 | Chr6 | 1604954 | 1607819 | + | 230 | 7.61 | 26825.46 | Cytoplasmic |
| CmGSTU2 | MELO3C006220T1 | 2 | 9 | Chr6 | 1794765 | 1797388 | - | 223 | 5.34 | 25700.81 | Nuclear |
| CmGSTU3 | MELO3C006221T1 | 2 | 12 | Chr6 | 1800092 | 1801458 | - | 301 | 6.61 | 35610.67 | Extracellular |
| CmGSTU4 | MELO3C006352T1 | 2 | 8 | Chr6 | 2752243 | 2753329 | - | 226 | 5.32 | 26239.22 | Nuclear |
| CmGSTU5 | MELO3C006353T1 | 2 | 8 | Chr6 | 2754761 | 2755527 | + | 226 | 5.71 | 26003.08 | Cytoplasmic |
| CmGSTU6 | MELO3C006354T1 | 2 | 8 | Chr6 | 2759182 | 2760098 | + | 253 | 5.3 | 28869.16 | Cytoplasmic |
| CmGSTU7 | MELO3C006356T1 | 4 | 2 | Chr6 | 2762454 | 2763279 | + | 150 | 10.13 | 16871.13 | Extracellular |
| CmGSTU8 | MELO3C006357T1 | 2 | 9 | Chr6 | 2763373 | 2764688 | + | 226 | 5.61 | 26633.63 | Nuclear |
| CmDHAR1 | MELO3C020204T1 | 6 | 5 | Chr6 | 15728451 | 15743335 | + | 298 | 8.67 | 33082.95 | Chloroplast |
| CmTCHQD1 | MELO3C010516T3 | 2 | 12 | Chr7 | 7738634 | 7742086 | + | 268 | 9.26 | 31389.31 | Plasma membrane |
| CmGSTU9 | MELO3C016166T1 | 2 | 9 | Chr7 | 20095849 | 20096890 | + | 220 | 6.14 | 25379.55 | Cytoplasmic |
| CmGSTU10 | MELO3C016167T1 | 2 | 9 | Chr7 | 20098212 | 20098990 | + | 221 | 6.01 | 25537.56 | Cytoplasmic |
| CmGSTU11 | MELO3C016168T1 | 2 | 9 | Chr7 | 20131419 | 20133053 | + | 219 | 6.83 | 25479.8 | Cytoplasmic |
| CmGSTU12 | MELO3C016169T1 | 2 | 9 | Chr7 | 20133818 | 20135426 | + | 218 | 5.65 | 25137.16 | Cytoplasmic |
| CmGSTT1 | MELO3C016349T2 | 7 | 7 | Chr7 | 22285579 | 22288211 | - | 239 | 9.47 | 27079.78 | Nuclear |
| CmGSTU13 | MELO3C003188T2 | 2 | 9 | Chr8 | 30753971 | 30755102 | + | 219 | 6.02 | 24892.04 | Nuclear |
| CmGSTU14 | MELO3C003190T1 | 2 | 9 | Chr8 | 30774554 | 30777775 | + | 225 | 5.77 | 26034.9 | Nuclear |
| CmGSTU15 | MELO3C003192T1 | 2 | 8 | Chr8 | 30790112 | 30791921 | + | 225 | 5.82 | 25553.47 | Nuclear |
| CmGSTU16 | MELO3C003194T1 | 2 | 9 | Chr8 | 30809773 | 30810516 | + | 221 | 5.5 | 25388.2 | Nuclear |
| CmGSTU17 | MELO3C003200T1 | 2 | 9 | Chr8 | 30870912 | 30872729 | - | 224 | 7.11 | 25759.95 | Cytoplasmic |
| CmGSTU18 | MELO3C003201T3 | 2 | 9 | Chr8 | 30874789 | 30875530 | - | 223 | 5.5 | 25656.49 | Nuclear |
| CmGSTU19 | MELO3C025097T1 | 2 | 8 | Chr9 | 14361607 | 14363117 | - | 225 | 5.35 | 25515.3 | Cytoplasmic |
| CmGSTU20 | MELO3C012092T1 | 2 | 7 | Chr10 | 2667773 | 2668842 | - | 230 | 6.76 | 26483.79 | Cytoplasmic |
| CmGSTL3 | MELO3C011714T2 | 10 | 12 | Chr10 | 5577290 | 5583915 | - | 298 | 8.87 | 33629.35 | Chloroplast |
| CmDHAR2 | MELO3C023826T2 | 6 | 5 | Chr10 | 5852550 | 5856866 | + | 215 | 6 | 23693.46 | Chloroplast |
| CmGSTZ2 | MELO3C023220T1 | 9 | 6 | Chr11 | 342908 | 345467 | + | 221 | 5.19 | 24797.71 | Cytoplasmic |
| CmGSTZ3 | MELO3C023224T1 | 10 | 6 | Chr11 | 361429 | 365338 | + | 223 | 6.53 | 24856.42 | Cytoplasmic |
| CmGSTU21 | MELO3C001978T1 | 1 | 5 | Chr12 | 25952044 | 25952513 | - | 134 | 5.17 | 15464.83 | Cytoplasmic |
| CmGSTU22 | MELO3C001977T1 | 2 | 5 | Chr12 | 25952888 | 25955196 | - | 203 | 9.08 | 23344.44 | Cytoplasmic |
| CmGSTU23 | MELO3C001175T1 | 1 | 3 | Chr0 | 24569942 | 24570262 | + | 107 | 4.72 | 12175.21 | Cytoplasmic |
| CmGSTU24 | MELO3C001702T1 | 1 | 2 | Chr0 | 33965495 | 33965711 | + | 72 | 4.64 | 8083.38 | Cytoplasmic |

**Table S2**

GST genes identified from the genomes of *Arabidopsis*, cucumber, zucchini squash, watermelon.

| **Species** | ***Arabidopsis*** | **Cucumber** | **Zucchini squash** | **Watermelon** |
| --- | --- | --- | --- | --- |
| Sum of GSTs | 81 | 36 | 35 | 35 |
|  | AT1G02940.4 | CsaV3_2G024500.1 | Cp4.1LG12g05490.1 | Cla97C05G082750.1 |
|  | AT1G78360.1 | CsaV3_3G011460.1 | Cp4.1LG03g18240.1 | Cla97C05G084430.1 |
|  | AT5G17220.1 | CsaV3_4G025660.1 | Cp4.1LG03g17400.1 | Cla97C04G074280.1 |
|  | AT1G66235.1 | CsaV3_1G004150.1 | Cp4.1LG08g03030.1 | Cla97C09G168680.1 |
|  | AT2G29450.1 | CsaV3_4G025640.1 | Cp4.1LG03g18220.1 | Cla97C05G106350.1 |
|  | AT5G16710.1 | CsaV3_5G006450.1 | Cp4.1LG04g13870.1 | Cla97C05G083070.1 |
|  | AT1G02940.1 | CsaV3_4G025680.1 | Cp4.1LG14g03330.1 | Cla97C01G008830.1 |
|  | AT1G59670.1 | CsaV3_1G005450.1 | Cp4.1LG05g10520.1 | Cla97C04G074260.1 |
|  | AT1G02940.2 | CsaV3_5G006680.1 | Cp4.1LG07g03380.1 | Cla97C07G135180.1 |
|  | AT1G10370.1 | CsaV3_2G019500.1 | Cp4.1LG03g17360.1 | Cla97C06G123950.1 |
|  | AT1G02940.3 | CsaV3_3G009990.1 | Cp4.1LG01g04640.1 | Cla97C04G079270.1 |
|  | AT3G55040.1 | CsaV3_4G025760.1 | Cp4.1LG01g10520.1 | Cla97C02G039140.1 |
|  | AT2G02390.1 | CsaV3_1G005420.1 | Cp4.1LG01g19720.1 | Cla97C07G137340.1 |
|  | AT5G02790.1 | CsaV3_7G029050.1 | Cp4.1LG14g02050.1 | Cla97C07G137320.1 |
|  | AT3G43800.1 | CsaV3_3G035360.1 | Cp4.1LG09g01690.1 | Cla97C11G221990.1 |
|  | AT2G02390.4 | CsaV3_3G020840.1 | Cp4.1LG01g00840.1 | Cla97C09G168660.1 |
|  | AT2G02390.2 | CsaV3_3G011450.1 | Cp4.1LG08g01860.1 | Cla97C04G074140.1 |
|  | AT2G02390.3 | CsaV3_4G025630.1 | Cp4.1LG10g08250.1 | Cla97C02G026610.1 |
|  | AT1G69930.1 | CsaV3_4G025650.1 | Cp4.1LG00g08570.1 | Cla97C05G084420.1 |
|  | AT2G29420.1 | CsaV3_1G005440.1 | Cp4.1LG14g03320.1 | Cla97C09G168690.1 |
|  | AT5G41210.1 | CsaV3_4G025690.1 | Cp4.1LG08g11330.1 | Cla97C04G074270.1 |
|  | AT1G53680.1 | CsaV3_4G007700.1 | Cp4.1LG05g10530.1 | Cla97C02G026570.1 |
|  | AT1G19570.1 | CsaV3_4G007720.1 | Cp4.1LG10g08160.1 | Cla97C04G074290.1 |
|  | AT1G77290.1 | CsaV3_4G028000.1 | Cp4.1LG02g02970.1 | Cla97C08G157490.1 |
|  | AT1G77290.3 | CsaV3_4G025770.1 | Cp4.1LG03g06220.1 | Cla97C05G093610.1 |
|  | AT1G77290.2 | CsaV3_3G035330.1 | Cp4.1LG01g00050.1 | Cla97C04G079280.1 |
|  | AT1G69920.1 | CsaV3_2G019530.1 | Cp4.1LG19g02950.1 | Cla97C04G074250.1 |
|  | AT5G41240.1 | CsaV3_4G025750.1 | Cp4.1LG19g02970.1 | Cla97C04G079260.1 |
|  | AT1G78340.1 | CsaV3_7G029060.1 | Cp4.1LG01g10570.1 | Cla97C02G039380.1 |
|  | AT5G62480.1 | CsaV3_4G006000.1 | Cp4.1LG17g07080.1 | Cla97C10G203890.1 |
|  | AT5G62480.2 | CsaV3_4G007690.1 | Cp4.1LG20g00370.1 | Cla97C07G137330.1 |
|  | AT1G59700.1 | CsaV3_7G029040.1 | Cp4.1LG06g06000.1 | Cla97C10G203900.1 |
|  | AT1G17180.1 | CsaV3_3G009690.1 | Cp4.1LG19g03180.1 | Cla97C09G168670.1 |
|  | AT5G62480.3 | CsaV3_5G020500.1 | Cp4.1LG17g07000.1 | Cla97C04G074150.1 |
|  | AT1G27140.1 | CsaV3_3G035350.1 | Cp4.1LG00g14780.1 | Cla97C04G074130.1 |
|  | AT1G02930.2 | CsaV3_5G032650.1 | |  |
|  | AT1G02930.1 | |  |  |
|  | AT2G29480.1 | |  |  |
|  | AT1G19550.1 | |  |  |
|  | AT3G47680.1 | |  |  |
|  | AT2G30870.1 | |  |  |
|  | AT2G02380.1 | |  |  |
|  | AT1G78320.2 | |  |  |
|  | AT1G78320.1 | |  |  |
|  | AT5G02780.3 | |  |  |
|  | AT5G02780.2 | |  |  |
|  | AT5G02780.1 | |  |  |
|  | AT5G41220.2 | |  |  |
|  | AT5G41220.3 | |  |  |
|  | AT5G41220.1 | |  |  |
|  | AT2G47730.2 | |  |  |
|  | AT2G47730.1 | |  |  |
|  | AT1G02950.5 | |  |  |
|  | AT1G78370.1 | |  |  |
|  | AT1G74590.1 | |  |  |
|  | AT4G02520.1 | |  |  |
|  | AT2G29460.1 | |  |  |
|  | AT1G10360.1 | |  |  |
|  | AT1G17170.1 | |  |  |
|  | AT2G29490.1 | |  |  |
|  | AT2G02930.1 | |  |  |
|  | AT1G02950.1 | |  |  |
|  | AT1G02950.2 | |  |  |
|  | AT1G02950.3 | |  |  |
|  | AT1G02950.4 | |  |  |
|  | AT1G02920.1 | |  |  |
|  | AT2G30860.1 | |  |  |
|  | AT2G30860.2 | |  |  |
|  | AT1G78380.1 | |  |  |
|  | AT2G29470.1 | |  |  |
|  | AT3G09270.1 | |  |  |
|  | AT3G03190.1 | |  |  |
|  | AT1G75270.1 | |  |  |
|  | AT2G29440.1 | |  |  |
|  | AT1G49860.1 | |  |  |
|  | AT1G17190.1 | |  |  |
|  | AT1G27130.1 | |  |  |
|  | AT3G62760.1 | |  |  |
|  | AT1G65820.1 | |  |  |
|  | AT1G65820.3 | |  |  |
|  | AT1G65820.2 | |  |  |

**Table S3**

Top 36 in network string interactions ranked by MCC method.

| **Rank** | **Name** | **Score** | |
| --- | --- | --- | --- |
| 1 | CmDHAR2 | 90 |  |
| 2 | CmDHAR1 | 84 |  |
| 3 | CmGSTF4 | 38 |  |
| 4 | CmGSTT1 | 12 |  |
| 4 | CmTCHQD1 | 12 |  |
| 4 | CmGSTZ1 | 12 |  |
| 4 | CmGSTL3 | 12 |  |
| 4 | CmGSTL2 | 12 |  |
| 9 | CmGSTZ3 | 10 |  |
| 10 | CmGSTZ2 | 8 |  |
| 11 | CmGSTU20 | 7 |  |
| 11 | CmGSTU21 | 7 |  |
| 13 | CmGSTL1 | 6 |  |
| 13 | CmGSTU3 | 6 |  |
| 15 | CmGSTU19 | 4 |  |
| 16 | CmGSTF1 | 3 |  |
| 16 | CmGSTF2 | 3 |  |
| 16 | CmGSTU1 | 3 |  |
| 16 | CmGSTF3 | 3 |  |
| 16 | CmGSTF5 | 3 |  |
| 21 | CmGSTU17 | 2 |  |
| 21 | CmGSTU13 | 2 |  |
| 21 | CmGSTU14 | 2 |  |
| 21 | CmGSTU16 | 2 |  |
| 21 | CmGSTU18 | 2 |  |
| 21 | CmGSTU10 | 2 |  |
| 21 | CmGSTU15 | 2 |  |
| 21 | CmGSTU11 | 2 |  |
| 21 | CmGSTU8 | 2 |  |
| 21 | CmGSTU9 | 2 |  |
| 21 | CmGSTU12 | 2 |  |
| 21 | CmGSTU2 | 2 |  |
| 21 | CmGSTU4 | 2 |  |
| 21 | CmGSTU5 | 2 |  |
| 21 | CmGSTU6 | 2 |  |
| 36 | CmGSTU23 | 1 |  |

**Table S4**

Validation of predicted 3D models.

| **Name** | **Template** | **Seq Identity (%)** | **QMEAN** | **LGscore** | **MaxSub** | **C-score** |
| --- | --- | --- | --- | --- | --- | --- |
| CmGSTU9 | 5agy.1.A | 68.84 | 0.12 | 6.959 | 0.697 |  |
| CmGSTU12 | 4chs.1.A | 64.65 | 0.2 | 5.980 | 0.656 |  |
| CmGSTL1 | 4pqi.1.A | 78.11 | -0.05 | 5.674 | 0.495 |  |
| CmGSTZ3 | 1e6b.1.A | 66.97 | -1.38 | 6.520 | 0.631 |  |
| CmDHAR2 | 5d9t.1.A | 67.14 | -0.84 | 4.741 | 0.384 |  |
| CmGSTT1 | 4mpf.1.A | 34.23 | -1.96 | 6.460 | 0.686 |  |
| CmGSTF5 | 4ri6.1.A | 69.3 | -0.27 | 5.54 | 0.536 |  |
| CmTCHQD1 | - | - | - | - | - | -0.93 |

**Note:**

Different ranges of quality:

LGscore > 1.5 fairly good model

LGscore > 2.5 very good model

LGscore > 4 extremly good model

MaxSub > 0.1 fairly good model

MaxSub > 0.5 very good model

MaxSub > 0.8 extremly good model

C-score is typically in the range of [-5,2], where a C-score of higher value signifies a model with a high confidence and vice-versa.

**Table S5**

Classification of *cis*-elements identified in the promoters of CmGST genes.

| **Clade** | **Motif** | **Description** |
| --- | --- | --- |
| light-responsive elements | G-Box | cis-acting regulatory element involved in light responsiveness |
|  | AE-box | part of a module for light response |
|  | GT1-motif | light responsive element |
|  | Sp1 | light responsive element |
|  | ATCT-motif | part of a conserved DNA module involved in light responsiveness |
|  | Box 4 | part of a conserved DNA module involved in light responsiveness |
|  | I-box | part of a light responsive element |
|  | MRE | MYB binding site involved in light responsiveness |
|  | GATA-motif | part of a light responsive element |
|  | TCCC-motif | part of a light responsive element |
|  | TCT-motif | part of a light responsive element |
|  | LAMP-element | part of a light responsive element |
|  | Gap-box | part of a light responsive element |
|  | 3-AF1 binding site | light responsive element |
|  | GTGGC-motif | part of a light responsive element |
| hormone-responsive elements | P-box | gibberellin-responsive element |
|  | TCA-element | cis-acting element involved in salicylic acid responsiveness |
|  | TGA-element | auxin-responsive element |
|  | TATC-box | cis-acting element involved in gibberellin-responsiveness |
|  | SARE | cis-acting element involved in salicylic acid responsiveness |
|  | ERE |  |
|  | TGACG-motif | cis-acting regulatory element involved in the MeJA-responsiveness |
|  | CGTCA-motif | cis-acting regulatory element involved in the MeJA-responsiveness |
|  | ABRE | cis-acting element involved in the abscisic acid responsiveness |
| development-related elements | GCN4_motif | cis-regulatory element involved in endosperm expression |
|  | circadian | cis-acting regulatory element involved in circadian control |
|  | CAT-box | cis-acting regulatory element related to meristem expression |
|  | CCGTCC-box | |
|  | MSA-like | cis-acting element involved in cell cycle regulation |
|  | dOCT |  |
|  | HD-Zip 1 | element involved in differentiation of the palisade mesophyll cells |
|  | O2-site | cis-acting regulatory element involved in zein metabolism regulation |
| abiotic stress-response elements | LTR | cis-acting element involved in low-temperature responsiveness |
|  | WUN-motif | |
|  | GC-motif | enhancer-like element involved in anoxic specific inducibility |
|  | ARE | cis-acting regulatory element essential for the anaerobic induction |
|  | MBS | MYB binding site involved in drought-inducibility |
|  | W box |  |
|  | TC-rich repeats | cis-acting element involved in defense and stress responsiveness |
| other elements | TCA |  |
|  | Unnamed__4 | |
|  | STRE |  |
|  | MYC |  |
|  | CAAT-box |  |

**Table S6**

*Cis*-elements information in the promoters of CmGST genes in Hami melon genome.

| **Gene ID** | **Motif** | **Seq** | **Start** | **Length** | **Strand** |
| --- | --- | --- | --- | --- | --- |
| CmDHAR1 | DRE core | GCCGAC | 3 | 6 | - |
| CmDHAR1 | Unnamed__4 | CTCC | 9 | 4 | + |
| CmDHAR1 | Unnamed__4 | CTCC | 101 | 4 | + |
| CmDHAR1 | Unnamed__4 | CTCC | 123 | 4 | + |
| CmDHAR1 | Unnamed__4 | CTCC | 264 | 4 | - |
| CmDHAR1 | Unnamed__4 | CTCC | 449 | 4 | + |
| CmDHAR1 | Unnamed__4 | CTCC | 852 | 4 | - |
| CmDHAR1 | CTAG-motif | ACTAGCAGAA | 411 | 9 | + |
| CmDHAR1 | AAGAA-motif | GAAAGAA | 35 | 7 | - |
| CmDHAR1 | AAGAA-motif | GAAAGAA | 639 | 7 | - |
| CmDHAR1 | CAAT-box | CAAAT | 366 | 5 | - |
| CmDHAR1 | CAAT-box | CAAT | 382 | 4 | - |
| CmDHAR1 | CAAT-box | CAAAT | 428 | 5 | + |
| CmDHAR1 | CAAT-box | CAAAT | 456 | 5 | + |
| CmDHAR1 | CAAT-box | CAAT | 525 | 4 | + |
| CmDHAR1 | CAAT-box | CAAT | 538 | 4 | + |
| CmDHAR1 | CAAT-box | CAAT | 574 | 4 | - |
| CmDHAR1 | CAAT-box | CAAT | 645 | 4 | + |
| CmDHAR1 | CAAT-box | CAAT | 678 | 4 | + |
| CmDHAR1 | CAAT-box | CAAT | 739 | 4 | - |
| CmDHAR1 | CAAT-box | CCAAT | 744 | 5 | - |
| CmDHAR1 | CAAT-box | CCAAT | 761 | 5 | - |
| CmDHAR1 | CAAT-box | CAAT | 846 | 4 | - |
| CmDHAR1 | CAAT-box | CAAT | 862 | 4 | - |
| CmDHAR1 | TGACG-motif | TGACG | 192 | 5 | - |
| CmDHAR1 | TATA-box | TATAA | 398 | 5 | - |
| CmDHAR1 | TATA-box | TATA | 399 | 4 | + |
| CmDHAR1 | TATA-box | TATA | 483 | 4 | - |
| CmDHAR1 | TATA-box | TATA | 754 | 4 | - |
| CmDHAR1 | STRE | AGGGG | 176 | 5 | - |
| CmDHAR1 | STRE | AGGGG | 446 | 5 | - |
| CmDHAR1 | MYB | CAACCA | 19 | 6 | + |
| CmDHAR1 | MYB | CAACAG | 170 | 6 | - |
| CmDHAR1 | MYB | CAACCA | 513 | 6 | - |
| CmDHAR1 | MYB | CAACCA | 867 | 6 | - |
| CmDHAR1 | MRE | AACCTAA | 886 | 7 | - |
| CmDHAR1 | GARE-motif | TCTGTTG | 169 | 7 | + |
| CmDHAR1 | GT1-motif | GGTTAA | 889 | 6 | + |
| CmDHAR1 | WRE3 | CCACCT | 75 | 6 | + |
| CmDHAR1 | WRE3 | CCACCT | 433 | 6 | + |
| CmDHAR1 | Myb-binding site | CAACAG | 170 | 6 | - |
| CmDHAR1 | as-1 | TGACG | 192 | 5 | - |
| CmDHAR1 |  | motif_sequence | 32 | 4 | + |
| CmDHAR1 |  | motif_sequence | 385 | 4 | - |
| CmDHAR1 |  | motif_sequence | 459 | 4 | + |
| CmDHAR1 |  | motif_sequence | 477 | 4 | + |
| CmDHAR1 |  | motif_sequence | 608 | 4 | - |
| CmDHAR1 |  | motif_sequence | 767 | 4 | + |
| CmDHAR1 |  | motif_sequence | 798 | 4 | - |
| CmDHAR1 |  | motif_sequence | 804 | 4 | - |
| CmDHAR1 | CGTCA-motif | CGTCA | 192 | 5 | + |
| CmDHAR1 | TGA-element | AACGAC | 358 | 6 | - |
| CmDHAR2 | Unnamed__4 | CTCC | 90 | 4 | - |
| CmDHAR2 | Unnamed__4 | CTCC | 93 | 4 | - |
| CmDHAR2 | Unnamed__4 | CTCC | 243 | 4 | - |
| CmDHAR2 | Unnamed__4 | CTCC | 272 | 4 | + |
| CmDHAR2 | Unnamed__4 | CTCC | 275 | 4 | + |
| CmDHAR2 | Unnamed__4 | CTCC | 360 | 4 | - |
| CmDHAR2 | Unnamed__4 | CTCC | 430 | 4 | - |
| CmDHAR2 | Unnamed__4 | CTCC | 621 | 4 | - |
| CmDHAR2 | AuxRR-core | GGTCCAT | 413 | 7 | - |
| CmDHAR2 | Myb | CAACTG | 144 | 6 | - |
| CmDHAR2 | Myb | TAACTG | 440 | 6 | + |
| CmDHAR2 | MBS | CAACTG | 144 | 6 | - |
| CmDHAR2 | WUN-motif | CAATTACAT | 605 | 9 | - |
| CmDHAR2 | W box | TTGACC | 18 | 6 | - |
| CmDHAR2 | Unnamed__1 | CGTGG | 228 | 5 | + |
| CmDHAR2 | CAAT-box | CCAAT | 49 | 5 | - |
| CmDHAR2 | CAAT-box | CAAAT | 111 | 5 | + |
| CmDHAR2 | CAAT-box | CAAT | 114 | 4 | - |
| CmDHAR2 | CAAT-box | CAAT | 126 | 4 | + |
| CmDHAR2 | CAAT-box | CAAAT | 201 | 5 | + |
| CmDHAR2 | CAAT-box | CAAT | 280 | 4 | + |
| CmDHAR2 | CAAT-box | CAAAT | 452 | 5 | - |
| CmDHAR2 | CAAT-box | CAAT | 487 | 4 | - |
| CmDHAR2 | CAAT-box | CAAT | 610 | 4 | - |
| CmDHAR2 | Myc | TCTCTTA | 78 | 7 | + |
| CmDHAR2 | MYB | CAACAG | 29 | 6 | - |
| CmDHAR2 | MYB | CAACAG | 446 | 6 | - |
| CmDHAR2 | I-box | AAGATAAGGCT | 257 | 10 | - |
| CmDHAR2 | MYC | CATTTG | 201 | 6 | - |
| CmDHAR2 | Myb-binding site | CAACAG | 29 | 6 | - |
| CmDHAR2 | Myb-binding site | CAACAG | 446 | 6 | - |
| CmDHAR2 |  | motif_sequence | 130 | 4 | + |
| CmDHAR2 |  | motif_sequence | 300 | 4 | - |
| CmDHAR2 |  | motif_sequence | 373 | 4 | - |
| CmDHAR2 |  | motif_sequence | 430 | 4 | - |
| CmDHAR2 |  | motif_sequence | 601 | 4 | - |
| CmDHAR2 | TCA-element | TCAGAAGAGG | 355 | 9 | + |
| CmGSTF1 | box S | AGCCACC | 316 | 7 | + |
| CmGSTF1 | ARE | AAACCA | 367 | 6 | + |
| CmGSTF1 | CAT-box | GCCACT | 43 | 6 | + |
| CmGSTF1 |  | motif_sequence | 184 | 4 | - |
| CmGSTF1 |  | motif_sequence | 310 | 4 | - |
| CmGSTF1 |  | motif_sequence | 506 | 4 | + |
| CmGSTF1 |  | motif_sequence | 515 | 4 | + |
| CmGSTF1 | TCCC-motif | TCTCCCT | 516 | 7 | + |
| CmGSTF1 | Myb-binding site | CAACAG | 287 | 6 | - |
| CmGSTF1 | Myb-binding site | CAACAG | 535 | 6 | - |
| CmGSTF1 | GTGGC-motif | GATTCTGTGGC | 541 | 10 | - |
| CmGSTF1 | MYB | CAACAG | 287 | 6 | - |
| CmGSTF1 | MYB | CAACAG | 535 | 6 | - |
| CmGSTF1 | CAAT-box | CAAT | 85 | 4 | + |
| CmGSTF1 | CAAT-box | CAAAT | 105 | 5 | + |
| CmGSTF1 | CAAT-box | CAAT | 220 | 4 | + |
| CmGSTF1 | CAAT-box | CAAT | 304 | 4 | - |
| CmGSTF1 | CAAT-box | CAAAT | 471 | 5 | + |
| CmGSTF1 | CAAT-box | CAAT | 547 | 4 | + |
| CmGSTF1 | CAAT-box | CAAT | 582 | 4 | + |
| CmGSTF1 | Myb | CAACTG | 256 | 6 | - |
| CmGSTF1 | Myb | CAACTG | 401 | 6 | - |
| CmGSTF1 | MBS | CAACTG | 256 | 6 | - |
| CmGSTF1 | MBS | CAACTG | 401 | 6 | - |
| CmGSTF1 | W box | TTGACC | 160 | 6 | - |
| CmGSTF1 | W box | TTGACC | 326 | 6 | + |
| CmGSTF1 | Unnamed__4 | CTCC | 118 | 4 | - |
| CmGSTF1 | Unnamed__4 | CTCC | 184 | 4 | - |
| CmGSTF1 | Unnamed__4 | CTCC | 481 | 4 | - |
| CmGSTF1 | Unnamed__4 | CTCC | 517 | 4 | + |
| CmGSTF2 | CGTCA-motif | CGTCA | 270 | 5 | + |
| CmGSTF2 | circadian | CAAAGATATC | 439 | 9 | - |
| CmGSTF2 | box S | AGCCACC | 588 | 7 | - |
| CmGSTF2 | ARE | AAACCA | 545 | 6 | + |
| CmGSTF2 | as-1 | TGACG | 270 | 5 | - |
| CmGSTF2 |  | motif_sequence | 64 | 4 | + |
| CmGSTF2 |  | motif_sequence | 139 | 4 | + |
| CmGSTF2 |  | motif_sequence | 223 | 4 | + |
| CmGSTF2 |  | motif_sequence | 309 | 4 | - |
| CmGSTF2 |  | motif_sequence | 503 | 4 | + |
| CmGSTF2 | TGACG-motif | TGACG | 270 | 5 | - |
| CmGSTF2 | CAAT-box | CAAT | 96 | 4 | + |
| CmGSTF2 | CAAT-box | CAAAT | 102 | 5 | + |
| CmGSTF2 | CAAT-box | CAAT | 105 | 4 | - |
| CmGSTF2 | CAAT-box | CCCAATTT | 130 | 8 | + |
| CmGSTF2 | CAAT-box | CCAAT | 131 | 5 | + |
| CmGSTF2 | CAAT-box | CAAT | 132 | 4 | + |
| CmGSTF2 | CAAT-box | CAAT | 147 | 4 | + |
| CmGSTF2 | CAAT-box | CAAAT | 160 | 5 | + |
| CmGSTF2 | CAAT-box | CAAT | 209 | 4 | + |
| CmGSTF2 | CAAT-box | CAAT | 217 | 4 | + |
| CmGSTF2 | CAAT-box | CAAAT | 230 | 5 | + |
| CmGSTF2 | CAAT-box | CAAT | 255 | 4 | - |
| CmGSTF2 | CAAT-box | CAAT | 301 | 4 | - |
| CmGSTF2 | CAAT-box | CAAT | 555 | 4 | - |
| CmGSTF2 | CAAT-box | CAAT | 579 | 4 | + |
| CmGSTF2 | P-box | CCTTTTG | 151 | 7 | + |
| CmGSTF2 | P-box | CCTTTTG | 409 | 7 | - |
| CmGSTF2 | I-box | gGATAAGGTG | 618 | 9 | + |
| CmGSTF2 | MYB | CAACCA | 401 | 6 | - |
| CmGSTF2 | MYC | CATGTG | 42 | 6 | - |
| CmGSTF2 | Unnamed__4 | CTCC | 141 | 4 | + |
| CmGSTF2 | Unnamed__4 | CTCC | 225 | 4 | + |
| CmGSTF2 | Unnamed__4 | CTCC | 309 | 4 | - |
| CmGSTF2 | Unnamed__4 | CTCC | 537 | 4 | - |
| CmGSTF2 | AuxRR-core | GGTCCAT | 15 | 7 | + |
| CmGSTF2 | AP-1 | TGAGTTAG | 420 | 8 | + |
| CmGSTF2 | W box | TTGACC | 157 | 6 | - |
| CmGSTF3 | W box | TTGACC | 157 | 6 | - |
| CmGSTF3 | MBS | CAACTG | 253 | 6 | - |
| CmGSTF3 | Myb | CAACTG | 253 | 6 | - |
| CmGSTF3 | Unnamed__10 | TCCACGTAGA | 292 | 9 | - |
| CmGSTF3 | Unnamed__4 | CTCC | 141 | 4 | + |
| CmGSTF3 | Unnamed__4 | CTCC | 309 | 4 | - |
| CmGSTF3 | Box 4 | ATTAAT | 145 | 6 | + |
| CmGSTF3 | STRE | AGGGG | 132 | 5 | - |
| CmGSTF3 | STRE | AGGGG | 480 | 5 | + |
| CmGSTF3 | CAAT-box | CCCAATTT | 27 | 8 | + |
| CmGSTF3 | CAAT-box | CCAAT | 28 | 5 | + |
| CmGSTF3 | CAAT-box | CAAT | 29 | 4 | + |
| CmGSTF3 | CAAT-box | CAAT | 217 | 4 | + |
| CmGSTF3 | CAAT-box | CAAAT | 372 | 5 | - |
| CmGSTF3 | CAAT-box | CAAAT | 503 | 5 | - |
| CmGSTF3 | CAAT-box | CCAAT | 518 | 5 | + |
| CmGSTF3 | CAAT-box | CAAT | 519 | 4 | + |
| CmGSTF3 | CAAT-box | TGCCAAC | 534 | 7 | - |
| CmGSTF3 | CAAT-box | CAAT | 639 | 4 | + |
| CmGSTF3 | ABRE | ACGTG | 42 | 5 | - |
| CmGSTF3 | ABRE | ACGTG | 295 | 5 | + |
| CmGSTF3 | ABRE | GCCGCGTGGC | 570 | 9 | + |
| CmGSTF3 | Unnamed__1 | CGTGG | 41 | 5 | - |
| CmGSTF3 | Unnamed__1 | CGTGG | 227 | 5 | - |
| CmGSTF3 | Unnamed__1 | CGTGG | 296 | 5 | + |
| CmGSTF3 | Unnamed__1 | CGTGG | 614 | 5 | + |
| CmGSTF3 | Unnamed__8 | TCCACGTAGA | 292 | 9 | - |
| CmGSTF3 |  | motif_sequence | 139 | 4 | + |
| CmGSTF3 |  | motif_sequence | 406 | 4 | - |
| CmGSTF3 |  | motif_sequence | 442 | 4 | + |
| CmGSTF3 |  | motif_sequence | 556 | 4 | + |
| CmGSTF3 | Unnamed__14 | TCCACGTAGA | 292 | 9 | - |
| CmGSTF3 | G-Box | CACGTT | 294 | 6 | - |
| CmGSTF3 | Unnamed__12 | TCCACGTAGA | 292 | 9 | - |
| CmGSTF3 | WRE3 | CCACCT | 328 | 6 | + |
| CmGSTF3 | TGA-element | AACGAC | 265 | 6 | + |
| CmGSTF3 | TGA-element | AACGAC | 345 | 6 | - |
| CmGSTF3 | box S | AGCCACC | 336 | 7 | - |
| CmGSTF3 | circadian | CAAAGATATC | 595 | 9 | - |
| CmGSTF3 | G-box | CACGTC | 42 | 6 | + |
| CmGSTF4 | W box | TTGACC | 407 | 6 | + |
| CmGSTF4 | Unnamed__4 | CTCC | 309 | 4 | - |
| CmGSTF4 | Unnamed__4 | CTCC | 420 | 4 | - |
| CmGSTF4 | Unnamed__4 | CTCC | 463 | 4 | - |
| CmGSTF4 | Unnamed__4 | CTCC | 508 | 4 | + |
| CmGSTF4 | Unnamed__4 | CTCC | 517 | 4 | + |
| CmGSTF4 | MYB | CAACCA | 127 | 6 | + |
| CmGSTF4 | MYB | CAACCA | 393 | 6 | + |
| CmGSTF4 | TATC-box | TATCCCA | 592 | 7 | - |
| CmGSTF4 | Box 4 | ATTAAT | 562 | 6 | - |
| CmGSTF4 | STRE | AGGGG | 635 | 5 | + |
| CmGSTF4 | TGACG-motif | TGACG | 44 | 5 | - |
| CmGSTF4 | TGACG-motif | TGACG | 51 | 5 | - |
| CmGSTF4 | CAAT-box | CAAT | 24 | 4 | - |
| CmGSTF4 | CAAT-box | CAAAT | 58 | 5 | - |
| CmGSTF4 | CAAT-box | CAAAT | 76 | 5 | + |
| CmGSTF4 | CAAT-box | CAAT | 111 | 4 | + |
| CmGSTF4 | CAAT-box | CAAT | 147 | 4 | + |
| CmGSTF4 | CAAT-box | CAAAT | 160 | 5 | + |
| CmGSTF4 | CAAT-box | CAAT | 179 | 4 | - |
| CmGSTF4 | CAAT-box | CAAAT | 195 | 5 | - |
| CmGSTF4 | CAAT-box | CAAT | 209 | 4 | + |
| CmGSTF4 | CAAT-box | CAAT | 261 | 4 | - |
| CmGSTF4 | CAAT-box | CAAT | 292 | 4 | - |
| CmGSTF4 | CAAT-box | CAAT | 301 | 4 | + |
| CmGSTF4 | CAAT-box | CAAT | 338 | 4 | + |
| CmGSTF4 | CAAT-box | CAAT | 406 | 4 | - |
| CmGSTF4 | CAAT-box | CAAT | 557 | 4 | + |
| CmGSTF4 | CAAT-box | CAAT | 631 | 4 | - |
| CmGSTF4 | ABRE | ACGTG | 42 | 5 | - |
| CmGSTF4 | ABRE | ACGTG | 443 | 5 | + |
| CmGSTF4 | ABRE | ACGTG | 525 | 5 | - |
| CmGSTF4 |  | motif_sequence | 175 | 4 | - |
| CmGSTF4 |  | motif_sequence | 254 | 4 | + |
| CmGSTF4 |  | motif_sequence | 506 | 4 | + |
| CmGSTF4 | Sp1 | GGGCGG | 609 | 6 | - |
| CmGSTF4 | as-1 | TGACG | 44 | 5 | - |
| CmGSTF4 | as-1 | TGACG | 51 | 5 | - |
| CmGSTF4 | G-Box | CACGTT | 525 | 6 | + |
| CmGSTF4 | O2-site | GTTGACGTGA | 41 | 9 | - |
| CmGSTF4 | WRE3 | CCACCT | 513 | 6 | + |
| CmGSTF4 | TCT-motif | TCTTAC | 186 | 6 | + |
| CmGSTF4 | box S | AGCCACC | 465 | 7 | + |
| CmGSTF4 | G-box | CACGTC | 42 | 6 | + |
| CmGSTF4 | G-box | CACGTC | 442 | 6 | - |
| CmGSTF4 | CGTCA-motif | CGTCA | 44 | 5 | + |
| CmGSTF4 | CGTCA-motif | CGTCA | 51 | 5 | + |
| CmGSTF5 | MBS | CAACTG | 358 | 6 | + |
| CmGSTF5 | W box | TTGACC | 160 | 6 | - |
| CmGSTF5 | Myb | TAACTG | 277 | 6 | - |
| CmGSTF5 | Myb | CAACTG | 358 | 6 | + |
| CmGSTF5 | CCAAT-box | CAACGG | 31 | 6 | - |
| CmGSTF5 | Unnamed__4 | CTCC | 48 | 4 | + |
| CmGSTF5 | Unnamed__4 | CTCC | 118 | 4 | - |
| CmGSTF5 | Unnamed__4 | CTCC | 192 | 4 | + |
| CmGSTF5 | Unnamed__4 | CTCC | 243 | 4 | - |
| CmGSTF5 | Unnamed__4 | CTCC | 315 | 4 | - |
| CmGSTF5 | Unnamed__4 | CTCC | 420 | 4 | - |
| CmGSTF5 | Unnamed__4 | CTCC | 463 | 4 | - |
| CmGSTF5 | Unnamed__4 | CTCC | 484 | 4 | - |
| CmGSTF5 | Unnamed__4 | CTCC | 558 | 4 | - |
| CmGSTF5 | Unnamed__4 | CTCC | 585 | 4 | - |
| CmGSTF5 | Unnamed__4 | CTCC | 627 | 4 | - |
| CmGSTF5 | MSA-like | (T/C)C(T/C)AACGG(T/C)(T/C)A | 520 | 8.5 | + |
| CmGSTF5 | STRE | AGGGG | 248 | 5 | + |
| CmGSTF5 | STRE | AGGGG | 317 | 5 | + |
| CmGSTF5 | MYC | CATTTG | 225 | 6 | + |
| CmGSTF5 | MYC | CATTTG | 514 | 6 | + |
| CmGSTF5 | MYB recognition site | CCGTTG | 31 | 6 | + |
| CmGSTF5 | MYB | CAACCA | 632 | 6 | - |
| CmGSTF5 | ABRE | ACGTG | 567 | 5 | - |
| CmGSTF5 | ABRE | ACGTG | 581 | 5 | + |
| CmGSTF5 | CAAT-box | CAAT | 212 | 4 | + |
| CmGSTF5 | CAAT-box | CAAAT | 226 | 5 | - |
| CmGSTF5 | CAAT-box | CAAT | 301 | 4 | + |
| CmGSTF5 | CAAT-box | CAAT | 307 | 4 | - |
| CmGSTF5 | CAAT-box | CAAT | 330 | 4 | + |
| CmGSTF5 | CAAT-box | CAAAT | 476 | 5 | - |
| CmGSTF5 | CAAT-box | CAAAT | 515 | 5 | - |
| CmGSTF5 | CAAT-box | CAACCAACTCC | 627 | 10 | - |
| CmGSTF5 | CAAT-box | CAAT | 639 | 4 | - |
| CmGSTF5 | Unnamed__1 | CGTGG | 582 | 5 | + |
| CmGSTF5 |  | motif_sequence | 105 | 4 | - |
| CmGSTF5 |  | motif_sequence | 190 | 4 | + |
| CmGSTF5 |  | motif_sequence | 196 | 4 | + |
| CmGSTF5 |  | motif_sequence | 214 | 4 | + |
| CmGSTF5 |  | motif_sequence | 262 | 4 | + |
| CmGSTF5 |  | motif_sequence | 453 | 4 | - |
| CmGSTF5 |  | motif_sequence | 463 | 4 | - |
| CmGSTF5 |  | motif_sequence | 484 | 4 | - |
| CmGSTF5 |  | motif_sequence | 549 | 4 | - |
| CmGSTF5 |  | motif_sequence | 654 | 4 | + |
| CmGSTF5 | Sp1 | GGGCGG | 554 | 6 | + |
| CmGSTF5 | WRE3 | CCACCT | 629 | 6 | - |
| CmGSTF5 | G-Box | CACGTT | 580 | 6 | - |
| CmGSTF5 | TCCC-motif | TCTCCCT | 191 | 7 | + |
| CmGSTF5 | TCT-motif | TCTTAC | 95 | 6 | + |
| CmGSTF5 | G-box | CACGTC | 567 | 6 | + |
| CmGSTL1 |  | motif_sequence | 16 | 4 | - |
| CmGSTL1 |  | motif_sequence | 64 | 4 | + |
| CmGSTL1 |  | motif_sequence | 219 | 4 | + |
| CmGSTL1 |  | motif_sequence | 317 | 4 | + |
| CmGSTL1 |  | motif_sequence | 355 | 4 | - |
| CmGSTL1 |  | motif_sequence | 399 | 4 | + |
| CmGSTL1 |  | motif_sequence | 493 | 4 | - |
| CmGSTL1 |  | motif_sequence | 625 | 4 | + |
| CmGSTL1 | G-Box | CACGTT | 116 | 6 | - |
| CmGSTL1 | TGA-element | AACGAC | 33 | 6 | - |
| CmGSTL1 | TCA-element | CCATCTTTTT | 177 | 9 | + |
| CmGSTL1 | Box II -like sequence | TCCGTGTACCA | 199 | 10 | - |
| CmGSTL1 | AT~TATA-box | TATATA | 648 | 6 | - |
| CmGSTL1 | CCAAT-box | CAACGG | 249 | 6 | + |
| CmGSTL1 | TC-rich repeats | GTTTTCTTAC | 547 | 9 | + |
| CmGSTL1 | Unnamed__4 | CTCC | 29 | 4 | + |
| CmGSTL1 | Unnamed__4 | CTCC | 59 | 4 | + |
| CmGSTL1 | Unnamed__4 | CTCC | 207 | 4 | - |
| CmGSTL1 | Unnamed__4 | CTCC | 221 | 4 | + |
| CmGSTL1 | Unnamed__4 | CTCC | 265 | 4 | - |
| CmGSTL1 | Unnamed__4 | CTCC | 322 | 4 | + |
| CmGSTL1 | Unnamed__4 | CTCC | 345 | 4 | - |
| CmGSTL1 | Unnamed__4 | CTCC | 487 | 4 | + |
| CmGSTL1 | Unnamed__4 | CTCC | 493 | 4 | - |
| CmGSTL1 | STRE | AGGGG | 480 | 5 | - |
| CmGSTL1 | TATA-box | TATACA | 86 | 6 | - |
| CmGSTL1 | TATA-box | TATA | 88 | 4 | + |
| CmGSTL1 | TATA-box | TATA | 215 | 4 | + |
| CmGSTL1 | TATA-box | TATA | 513 | 4 | - |
| CmGSTL1 | TATA-box | TATACA | 646 | 6 | - |
| CmGSTL1 | TATA-box | TATATA | 648 | 6 | - |
| CmGSTL1 | TATA-box | ATATAT | 649 | 6 | - |
| CmGSTL1 | TATA-box | TATA | 650 | 4 | - |
| CmGSTL1 | MYB recognition site | CCGTTG | 249 | 6 | - |
| CmGSTL1 | MYC | CATTTG | 99 | 6 | + |
| CmGSTL1 | MYC | CATTTG | 326 | 6 | - |
| CmGSTL1 | MYC | CATTTG | 527 | 6 | + |
| CmGSTL1 | ABRE | ACGTG | 117 | 5 | + |
| CmGSTL1 | CAAT-box | CAAAT | 100 | 5 | - |
| CmGSTL1 | CAAT-box | CAAT | 177 | 4 | + |
| CmGSTL1 | CAAT-box | CAAAT | 326 | 5 | + |
| CmGSTL1 | CAAT-box | CAAT | 439 | 4 | + |
| CmGSTL1 | CAAT-box | CAAAT | 441 | 5 | - |
| CmGSTL1 | CAAT-box | CAAAT | 471 | 5 | - |
| CmGSTL1 | CAAT-box | CAAAT | 528 | 5 | - |
| CmGSTL1 | CAAT-box | CAAT | 571 | 4 | - |
| CmGSTL1 | CAAT-box | CAAT | 610 | 4 | - |
| CmGSTL1 | CAAT-box | CAAT | 644 | 4 | - |
| CmGSTL2 | GATA-motif | AAGATAAGATT | 161 | 10 | + |
| CmGSTL2 | ARE | AAACCA | 707 | 6 | - |
| CmGSTL2 | TCA-element | TCAGAAGAGG | 192 | 9 | + |
| CmGSTL2 | AT~TATA-box | TATATA | 95 | 6 | + |
| CmGSTL2 | CGTCA-motif | CGTCA | 41 | 5 | + |
| CmGSTL2 |  | motif_sequence | 73 | 4 | + |
| CmGSTL2 |  | motif_sequence | 195 | 4 | - |
| CmGSTL2 |  | motif_sequence | 364 | 4 | - |
| CmGSTL2 |  | motif_sequence | 470 | 4 | + |
| CmGSTL2 |  | motif_sequence | 522 | 4 | - |
| CmGSTL2 | as-1 | TGACG | 41 | 5 | - |
| CmGSTL2 | MYC | CATTTG | 116 | 6 | + |
| CmGSTL2 | TATA-box | TATATAA | 94 | 7 | - |
| CmGSTL2 | TATA-box | TATATA | 95 | 6 | + |
| CmGSTL2 | TATA-box | ATATAT | 96 | 6 | + |
| CmGSTL2 | TATA-box | TATA | 97 | 4 | + |
| CmGSTL2 | TATA-box | TATA | 211 | 4 | + |
| CmGSTL2 | TATA-box | TATAA | 378 | 5 | - |
| CmGSTL2 | TATA-box | TATA | 379 | 4 | - |
| CmGSTL2 | TATA-box | ATATAT | 531 | 6 | - |
| CmGSTL2 | TATA-box | TATA | 532 | 4 | - |
| CmGSTL2 | TGACG-motif | TGACG | 41 | 5 | - |
| CmGSTL2 | GC-motif | CCCCCG | 37 | 6 | + |
| CmGSTL2 | CAAT-box | CCAAT | 45 | 5 | - |
| CmGSTL2 | CAAT-box | CAAT | 110 | 4 | - |
| CmGSTL2 | CAAT-box | CAAAT | 117 | 5 | - |
| CmGSTL2 | CAAT-box | CAAT | 258 | 4 | + |
| CmGSTL2 | CAAT-box | CCAAT | 271 | 5 | - |
| CmGSTL2 | CAAT-box | CAAAT | 287 | 5 | - |
| CmGSTL2 | CAAT-box | CAAAT | 294 | 5 | + |
| CmGSTL2 | CAAT-box | CAAT | 355 | 4 | + |
| CmGSTL2 | CAAT-box | CAAAT | 357 | 5 | - |
| CmGSTL2 | CAAT-box | CAAAT | 480 | 5 | - |
| CmGSTL2 | CAAT-box | CAAT | 508 | 4 | + |
| CmGSTL2 | CAAT-box | CAAT | 526 | 4 | - |
| CmGSTL2 | CAAT-box | CAAT | 622 | 4 | - |
| CmGSTL2 | CAAT-box | CAAT | 640 | 4 | - |
| CmGSTL2 | CAAT-box | CAAT | 679 | 4 | - |
| CmGSTL2 | W box | TTGACC | 505 | 6 | - |
| CmGSTL2 | DRE core | GCCGAC | 52 | 6 | + |
| CmGSTL2 | Unnamed__4 | CTCC | 68 | 4 | + |
| CmGSTL2 | Unnamed__4 | CTCC | 274 | 4 | - |
| CmGSTL2 | Unnamed__4 | CTCC | 462 | 4 | - |
| CmGSTL2 | AuxRR-core | GGTCCAT | 488 | 7 | - |
| CmGSTL3 | WRE3 | CCACCT | 241 | 6 | + |
| CmGSTL3 | WRE3 | CCACCT | 406 | 6 | + |
| CmGSTL3 | O2-site | GATGATGTGG | 613 | 9 | + |
| CmGSTL3 | ABRE3a | TACGTG | 556 | 6 | + |
| CmGSTL3 |  | motif_sequence | 42 | 4 | + |
| CmGSTL3 |  | motif_sequence | 63 | 4 | + |
| CmGSTL3 |  | motif_sequence | 81 | 4 | + |
| CmGSTL3 |  | motif_sequence | 143 | 4 | - |
| CmGSTL3 |  | motif_sequence | 155 | 4 | - |
| CmGSTL3 |  | motif_sequence | 250 | 4 | + |
| CmGSTL3 |  | motif_sequence | 298 | 4 | - |
| CmGSTL3 |  | motif_sequence | 365 | 4 | + |
| CmGSTL3 |  | motif_sequence | 430 | 4 | - |
| CmGSTL3 |  | motif_sequence | 451 | 4 | - |
| CmGSTL3 |  | motif_sequence | 541 | 4 | - |
| CmGSTL3 |  | motif_sequence | 598 | 4 | - |
| CmGSTL3 |  | motif_sequence | 639 | 4 | + |
| CmGSTL3 |  | motif_sequence | 658 | 4 | - |
| CmGSTL3 |  | motif_sequence | 801 | 4 | - |
| CmGSTL3 |  | motif_sequence | 850 | 4 | - |
| CmGSTL3 | Box II -like sequence | TCCGTGTACCA | 385 | 10 | - |
| CmGSTL3 | LTR | CCGAAA | 727 | 6 | - |
| CmGSTL3 | G-box | TACGTG | 556 | 6 | + |
| CmGSTL3 | circadian | CAAAGATATC | 58 | 9 | - |
| CmGSTL3 | ARE | AAACCA | 581 | 6 | - |
| CmGSTL3 | ABRE4 | CACGTA | 556 | 6 | - |
| CmGSTL3 | GATA-motif | GATAGGA | 246 | 7 | - |
| CmGSTL3 | AuxRR-core | GGTCCAT | 664 | 7 | + |
| CmGSTL3 | Unnamed__4 | CTCC | 36 | 4 | + |
| CmGSTL3 | Unnamed__4 | CTCC | 105 | 4 | + |
| CmGSTL3 | Unnamed__4 | CTCC | 122 | 4 | + |
| CmGSTL3 | Unnamed__4 | CTCC | 215 | 4 | + |
| CmGSTL3 | Unnamed__4 | CTCC | 245 | 4 | + |
| CmGSTL3 | Unnamed__4 | CTCC | 451 | 4 | - |
| CmGSTL3 | Unnamed__4 | CTCC | 598 | 4 | - |
| CmGSTL3 | Unnamed__4 | CTCC | 658 | 4 | - |
| CmGSTL3 | Unnamed__4 | CTCC | 710 | 4 | + |
| CmGSTL3 | Unnamed__4 | CTCC | 801 | 4 | - |
| CmGSTL3 | AAGAA-motif | GAAAGAA | 116 | 7 | - |
| CmGSTL3 | AAGAA-motif | GAAAGAA | 564 | 7 | - |
| CmGSTL3 | ABRE | ACGTG | 557 | 5 | + |
| CmGSTL3 | CAAT-box | CAAT | 349 | 4 | + |
| CmGSTL3 | CAAT-box | CCAAT | 351 | 5 | - |
| CmGSTL3 | CAAT-box | CAAT | 478 | 4 | - |
| CmGSTL3 | CAAT-box | CAAT | 486 | 4 | + |
| CmGSTL3 | CAAT-box | CAAAT | 624 | 5 | - |
| CmGSTL3 | CAAT-box | CAAT | 718 | 4 | - |
| CmGSTL3 | CAAT-box | CCAAT | 741 | 5 | - |
| CmGSTL3 | CAAT-box | CAAT | 796 | 4 | - |
| CmGSTL3 | TATA-box | TATAA | 282 | 5 | - |
| CmGSTL3 | TATA-box | TATA | 283 | 4 | + |
| CmGSTL3 | TATA-box | ATATAT | 474 | 6 | - |
| CmGSTL3 | TATA-box | TATA | 475 | 4 | - |
| CmGSTL3 | TATA-box | TATA | 699 | 4 | - |
| CmGSTL3 | TATA-box | TATAA | 864 | 5 | - |
| CmGSTL3 | TATA-box | TATA | 865 | 4 | - |
| CmGSTL3 | MYC | CAATTG | 349 | 6 | + |
| CmGSTL3 | MYC | CATTTG | 623 | 6 | + |
| CmGSTL3 | MYB | CAACCA | 86 | 6 | + |
| CmGSTT1 | ARE | AAACCA | 490 | 6 | + |
| CmGSTT1 | ARE | AAACCA | 515 | 6 | - |
| CmGSTT1 | box S | AGCCACC | 481 | 7 | - |
| CmGSTT1 | CGTCA-motif | CGTCA | 165 | 5 | + |
| CmGSTT1 | circadian | CAAAGATATC | 135 | 9 | + |
| CmGSTT1 | A-box | CCGTCC | 47 | 6 | + |
| CmGSTT1 | A-box | CCGTCC | 375 | 6 | + |
| CmGSTT1 | CCGTCC motif | CCGTCC | 47 | 6 | + |
| CmGSTT1 | CCGTCC motif | CCGTCC | 375 | 6 | + |
| CmGSTT1 | CCGTCC-box | CCGTCC | 47 | 6 | + |
| CmGSTT1 | CCGTCC-box | CCGTCC | 375 | 6 | + |
| CmGSTT1 |  | motif_sequence | 19 | 4 | - |
| CmGSTT1 |  | motif_sequence | 55 | 4 | + |
| CmGSTT1 |  | motif_sequence | 179 | 4 | - |
| CmGSTT1 |  | motif_sequence | 190 | 4 | + |
| CmGSTT1 |  | motif_sequence | 214 | 4 | + |
| CmGSTT1 |  | motif_sequence | 244 | 4 | - |
| CmGSTT1 |  | motif_sequence | 353 | 4 | + |
| CmGSTT1 |  | motif_sequence | 411 | 4 | - |
| CmGSTT1 | GT1-motif | GGTTAAT | 66 | 7 | + |
| CmGSTT1 | WRE3 | CCACCT | 480 | 6 | - |
| CmGSTT1 | as-1 | TGACG | 165 | 5 | - |
| CmGSTT1 | STRE | AGGGG | 458 | 5 | + |
| CmGSTT1 | Box 4 | ATTAAT | 339 | 6 | + |
| CmGSTT1 | CAAT-box | CAAT | 27 | 4 | - |
| CmGSTT1 | CAAT-box | CAAT | 76 | 4 | - |
| CmGSTT1 | CAAT-box | CAAAT | 108 | 5 | + |
| CmGSTT1 | CAAT-box | CCAAT | 148 | 5 | + |
| CmGSTT1 | CAAT-box | CAAT | 149 | 4 | + |
| CmGSTT1 | CAAT-box | CAAT | 169 | 4 | - |
| CmGSTT1 | CAAT-box | CCAAT | 297 | 5 | - |
| CmGSTT1 | CAAT-box | CCAAT | 302 | 5 | - |
| CmGSTT1 | CAAT-box | CAAT | 348 | 4 | + |
| CmGSTT1 | CAAT-box | CAAT | 439 | 4 | - |
| CmGSTT1 | CAAT-box | CAACCAACTCC | 594 | 10 | - |
| CmGSTT1 | CAAT-box | CAAT | 693 | 4 | - |
| CmGSTT1 | CAAT-box | CAAT | 699 | 4 | - |
| CmGSTT1 | CAAT-box | CAAAT | 712 | 5 | - |
| CmGSTT1 | TGACG-motif | TGACG | 165 | 5 | - |
| CmGSTT1 | GCN4_motif | TGAGTCA | 441 | 7 | + |
| CmGSTT1 | Unnamed__4 | CTCC | 87 | 4 | - |
| CmGSTT1 | Unnamed__4 | CTCC | 125 | 4 | + |
| CmGSTT1 | Unnamed__4 | CTCC | 275 | 4 | - |
| CmGSTT1 | Unnamed__4 | CTCC | 335 | 4 | + |
| CmGSTT1 | Unnamed__4 | CTCC | 560 | 4 | - |
| CmGSTT1 | Unnamed__4 | CTCC | 594 | 4 | - |
| CmGSTT1 | Unnamed__4 | CTCC | 676 | 4 | + |
| CmGSTU1 |  | motif_sequence | 4 | 4 | - |
| CmGSTU1 |  | motif_sequence | 90 | 4 | - |
| CmGSTU1 |  | motif_sequence | 161 | 4 | + |
| CmGSTU1 |  | motif_sequence | 187 | 4 | + |
| CmGSTU1 |  | motif_sequence | 253 | 4 | - |
| CmGSTU1 |  | motif_sequence | 349 | 4 | - |
| CmGSTU1 |  | motif_sequence | 367 | 4 | - |
| CmGSTU1 |  | motif_sequence | 433 | 4 | - |
| CmGSTU1 | G-Box | CACGTT | 571 | 6 | + |
| CmGSTU1 | O2-site | GATGATGTGG | 496 | 9 | + |
| CmGSTU1 | AE-box | AGAAACAA | 109 | 8 | + |
| CmGSTU1 | CAT-box | GCCACT | 476 | 6 | + |
| CmGSTU1 | G-box | CACGTC | 225 | 6 | - |
| CmGSTU1 | AAGAA-motif | GAAAGAA | 416 | 7 | + |
| CmGSTU1 | MYB-like sequence | TAACCA | 444 | 6 | - |
| CmGSTU1 | Myb | TAACTG | 578 | 6 | - |
| CmGSTU1 | Unnamed__4 | CTCC | 42 | 4 | + |
| CmGSTU1 | Unnamed__4 | CTCC | 189 | 4 | + |
| CmGSTU1 | Unnamed__4 | CTCC | 234 | 4 | - |
| CmGSTU1 | Unnamed__4 | CTCC | 346 | 4 | - |
| CmGSTU1 | Unnamed__4 | CTCC | 433 | 4 | - |
| CmGSTU1 | Unnamed__4 | CTCC | 505 | 4 | - |
| CmGSTU1 | Unnamed__4 | CTCC | 632 | 4 | - |
| CmGSTU1 | MYB | TAACCA | 444 | 6 | - |
| CmGSTU1 | I-box | AAGATAAGGCT | 356 | 10 | + |
| CmGSTU1 | TATA-box | TAAAGATT | 11 | 8 | + |
| CmGSTU1 | TATA-box | TATACA | 144 | 6 | - |
| CmGSTU1 | TATA-box | TATA | 146 | 4 | + |
| CmGSTU1 | STRE | AGGGG | 629 | 5 | + |
| CmGSTU1 | MYC | CATTTG | 385 | 6 | + |
| CmGSTU1 | Unnamed__1 | CGTGG | 227 | 5 | + |
| CmGSTU1 | Unnamed__1 | CGTGG | 570 | 5 | - |
| CmGSTU1 | ABRE | ACGTG | 226 | 5 | + |
| CmGSTU1 | ABRE | ACGTG | 571 | 5 | - |
| CmGSTU1 | CAAT-box | CCAAT | 19 | 5 | - |
| CmGSTU1 | CAAT-box | CAAT | 79 | 4 | - |
| CmGSTU1 | CAAT-box | CAAT | 174 | 4 | + |
| CmGSTU1 | CAAT-box | CAAT | 208 | 4 | - |
| CmGSTU1 | CAAT-box | CAAT | 246 | 4 | - |
| CmGSTU1 | CAAT-box | CCAAT | 272 | 5 | + |
| CmGSTU1 | CAAT-box | CAAT | 273 | 4 | + |
| CmGSTU1 | CAAT-box | CAAAT | 386 | 5 | - |
| CmGSTU1 | CAAT-box | CCAAT | 442 | 5 | - |
| CmGSTU1 | CAAT-box | CAAAT | 472 | 5 | - |
| CmGSTU1 | CAAT-box | CAAAT | 662 | 5 | + |
| CmGSTU10 | ARE | AAACCA | 468 | 6 | - |
| CmGSTU10 | ARE | AAACCA | 490 | 6 | - |
| CmGSTU10 | TCT-motif | TCTTAC | 443 | 6 | - |
| CmGSTU10 | GATA-motif | AAGGATAAGG | 204 | 9 | - |
| CmGSTU10 | GATA-motif | AAGGATAAGG | 235 | 9 | + |
| CmGSTU10 | GARE-motif | TCTGTTG | 252 | 7 | + |
| CmGSTU10 | G-Box | CACGTT | 362 | 6 | - |
| CmGSTU10 | Myb-binding site | CAACAG | 253 | 6 | - |
| CmGSTU10 | F-box | CTATTCTCATT | 54 | 10 | - |
| CmGSTU10 |  | motif_sequence | 70 | 4 | - |
| CmGSTU10 |  | motif_sequence | 103 | 4 | - |
| CmGSTU10 |  | motif_sequence | 198 | 4 | + |
| CmGSTU10 |  | motif_sequence | 336 | 4 | - |
| CmGSTU10 |  | motif_sequence | 355 | 4 | - |
| CmGSTU10 | ABRE | ACGTG | 363 | 5 | + |
| CmGSTU10 | CAAT-box | CCAAT | 21 | 5 | - |
| CmGSTU10 | CAAT-box | CAAAT | 125 | 5 | - |
| CmGSTU10 | CAAT-box | CAAAT | 136 | 5 | + |
| CmGSTU10 | CAAT-box | CCAAT | 145 | 5 | + |
| CmGSTU10 | CAAT-box | CAAT | 146 | 4 | + |
| CmGSTU10 | CAAT-box | CAAAT | 190 | 5 | - |
| CmGSTU10 | CAAT-box | CAAT | 220 | 4 | - |
| CmGSTU10 | CAAT-box | CAAAT | 288 | 5 | + |
| CmGSTU10 | CAAT-box | CAAT | 403 | 4 | + |
| CmGSTU10 | CAAT-box | CCAAT | 405 | 5 | - |
| CmGSTU10 | CAAT-box | CAAAT | 456 | 5 | - |
| CmGSTU10 | CAAT-box | CCAAT | 466 | 5 | - |
| CmGSTU10 | CAAT-box | CCAAT | 475 | 5 | - |
| CmGSTU10 | CAAT-box | CCAAT | 511 | 5 | - |
| CmGSTU10 | CAAT-box | CAAT | 526 | 4 | - |
| CmGSTU10 | CAAT-box | CAAT | 612 | 4 | + |
| CmGSTU10 | CAAT-box | CAAT | 622 | 4 | + |
| CmGSTU10 | CAAT-box | CAAAT | 624 | 5 | - |
| CmGSTU10 | TATA-box | TATACA | 90 | 6 | - |
| CmGSTU10 | TATA-box | TATA | 92 | 4 | + |
| CmGSTU10 | TATA-box | TATA | 217 | 4 | + |
| CmGSTU10 | TATA-box | TATA | 320 | 4 | + |
| CmGSTU10 | TATA-box | TATAAAA | 493 | 7 | - |
| CmGSTU10 | TATA-box | TATAAA | 494 | 6 | - |
| CmGSTU10 | TATA-box | TATAA | 495 | 5 | - |
| CmGSTU10 | TATA-box | TATA | 496 | 4 | - |
| CmGSTU10 | MYC | CATTTG | 136 | 6 | - |
| CmGSTU10 | MYC | CATTTG | 189 | 6 | + |
| CmGSTU10 | MYC | CAATTG | 403 | 6 | - |
| CmGSTU10 | MYB | CAACAG | 253 | 6 | - |
| CmGSTU10 | MYB | CAACCA | 629 | 6 | - |
| CmGSTU10 | Unnamed__4 | CTCC | 6 | 4 | - |
| CmGSTU10 | Unnamed__4 | CTCC | 245 | 4 | + |
| CmGSTU10 | Unnamed__4 | CTCC | 355 | 4 | - |
| CmGSTU10 | Unnamed__4 | CTCC | 381 | 4 | - |
| CmGSTU10 | Unnamed__4 | CTCC | 439 | 4 | - |
| CmGSTU10 | AAGAA-motif | gGTAAAGAAA | 558 | 9 | + |
| CmGSTU11 | GATA-motif | AAGGATAAGG | 235 | 9 | + |
| CmGSTU11 | MYC | CATTTG | 136 | 6 | - |
| CmGSTU11 | MYC | CATTTG | 189 | 6 | + |
| CmGSTU11 | MYC | CATTTG | 631 | 6 | - |
| CmGSTU11 | ARE | AAACCA | 487 | 6 | - |
| CmGSTU11 | TATA-box | TATA | 217 | 4 | + |
| CmGSTU11 | TATA-box | TACAAAA | 569 | 7 | + |
| CmGSTU11 | CAAT-box | CAAAT | 136 | 5 | + |
| CmGSTU11 | CAAT-box | CCAAT | 145 | 5 | + |
| CmGSTU11 | CAAT-box | CAAT | 146 | 4 | + |
| CmGSTU11 | CAAT-box | CAAAT | 190 | 5 | - |
| CmGSTU11 | CAAT-box | CAAT | 220 | 4 | - |
| CmGSTU11 | CAAT-box | CAAT | 393 | 4 | - |
| CmGSTU11 | CAAT-box | CAAAT | 453 | 5 | - |
| CmGSTU11 | CAAT-box | CCAAT | 472 | 5 | - |
| CmGSTU11 | CAAT-box | CCAAT | 508 | 5 | - |
| CmGSTU11 | CAAT-box | CAAT | 523 | 4 | - |
| CmGSTU11 | CAAT-box | CAAAT | 631 | 5 | + |
| CmGSTU11 | ACTCATCCT sequence | ACTCATCCT | 259 | 9 | + |
| CmGSTU11 |  | motif_sequence | 40 | 4 | + |
| CmGSTU11 |  | motif_sequence | 103 | 4 | - |
| CmGSTU11 |  | motif_sequence | 156 | 4 | - |
| CmGSTU11 |  | motif_sequence | 164 | 4 | + |
| CmGSTU11 |  | motif_sequence | 198 | 4 | + |
| CmGSTU11 |  | motif_sequence | 352 | 4 | - |
| CmGSTU11 | AAGAA-motif | gGTAAAGAAA | 555 | 9 | + |
| CmGSTU11 | Unnamed__4 | CTCC | 245 | 4 | + |
| CmGSTU11 | Unnamed__4 | CTCC | 352 | 4 | - |
| CmGSTU11 | Unnamed__4 | CTCC | 357 | 4 | - |
| CmGSTU11 | Unnamed__4 | CTCC | 378 | 4 | - |
| CmGSTU11 | Unnamed__4 | CTCC | 420 | 4 | - |
| CmGSTU11 | Unnamed__4 | CTCC | 436 | 4 | - |
| CmGSTU11 | Gap-box | CAAATGAA(A/G)A | 631 | 9.5 | + |
| CmGSTU12 | CAAT-box | CAAAT | 136 | 5 | + |
| CmGSTU12 | CAAT-box | CCAAT | 145 | 5 | + |
| CmGSTU12 | CAAT-box | CAAT | 146 | 4 | + |
| CmGSTU12 | CAAT-box | CAAAT | 190 | 5 | - |
| CmGSTU12 | CAAT-box | CAAT | 208 | 4 | - |
| CmGSTU12 | CAAT-box | CAAT | 220 | 4 | - |
| CmGSTU12 | CAAT-box | CCAAT | 463 | 5 | - |
| CmGSTU12 | CAAT-box | CCAAT | 472 | 5 | - |
| CmGSTU12 | CAAT-box | CAAT | 506 | 4 | + |
| CmGSTU12 | CAAT-box | CCAAT | 508 | 5 | - |
| CmGSTU12 | CAAT-box | CAAT | 523 | 4 | - |
| CmGSTU12 | CAAT-box | CAAT | 631 | 4 | + |
| CmGSTU12 | MYB | CAACAG | 250 | 6 | - |
| CmGSTU12 | TATA-box | TATA | 217 | 4 | + |
| CmGSTU12 | TATA-box | TATA | 338 | 4 | - |
| CmGSTU12 | TATA-box | TATAA | 383 | 5 | - |
| CmGSTU12 | TATA-box | TATA | 384 | 4 | - |
| CmGSTU12 | STRE | AGGGG | 417 | 5 | + |
| CmGSTU12 | MYC | CATTTG | 136 | 6 | - |
| CmGSTU12 | MYC | CATTTG | 189 | 6 | + |
| CmGSTU12 | MYC | CAATTG | 506 | 6 | - |
| CmGSTU12 | Unnamed__4 | CTCC | 4 | 4 | - |
| CmGSTU12 | Unnamed__4 | CTCC | 245 | 4 | + |
| CmGSTU12 | Unnamed__4 | CTCC | 352 | 4 | - |
| CmGSTU12 | Unnamed__4 | CTCC | 357 | 4 | - |
| CmGSTU12 | Unnamed__4 | CTCC | 363 | 4 | - |
| CmGSTU12 | Unnamed__4 | CTCC | 367 | 4 | - |
| CmGSTU12 | Unnamed__4 | CTCC | 378 | 4 | - |
| CmGSTU12 | Unnamed__4 | CTCC | 436 | 4 | - |
| CmGSTU12 | Unnamed__4 | CTCC | 576 | 4 | - |
| CmGSTU12 | Myb | CAACTG | 400 | 6 | + |
| CmGSTU12 | MBS | CAACTG | 400 | 6 | + |
| CmGSTU12 | CCGTCC motif | CCGTCC | 256 | 6 | + |
| CmGSTU12 | A-box | CCGTCC | 256 | 6 | + |
| CmGSTU12 | GATA-motif | AAGGATAAGG | 235 | 9 | + |
| CmGSTU12 | ARE | AAACCA | 465 | 6 | - |
| CmGSTU12 | ARE | AAACCA | 487 | 6 | - |
| CmGSTU12 | TCCC-motif | TCTCCCT | 350 | 7 | - |
| CmGSTU12 | Myb-binding site | CAACAG | 250 | 6 | - |
| CmGSTU12 | GARE-motif | TCTGTTG | 249 | 7 | + |
| CmGSTU12 |  | motif_sequence | 4 | 4 | - |
| CmGSTU12 |  | motif_sequence | 70 | 4 | - |
| CmGSTU12 |  | motif_sequence | 156 | 4 | - |
| CmGSTU12 |  | motif_sequence | 198 | 4 | + |
| CmGSTU12 |  | motif_sequence | 312 | 4 | - |
| CmGSTU12 |  | motif_sequence | 333 | 4 | - |
| CmGSTU12 |  | motif_sequence | 352 | 4 | - |
| CmGSTU12 |  | motif_sequence | 480 | 4 | + |
| CmGSTU12 |  | motif_sequence | 576 | 4 | - |
| CmGSTU12 |  | motif_sequence | 593 | 4 | + |
| CmGSTU12 | CCGTCC-box | CCGTCC | 256 | 6 | + |
| CmGSTU13 | Unnamed__4 | CTCC | 352 | 4 | - |
| CmGSTU13 | Unnamed__4 | CTCC | 363 | 4 | - |
| CmGSTU13 | Unnamed__4 | CTCC | 387 | 4 | - |
| CmGSTU13 | Unnamed__4 | CTCC | 439 | 4 | - |
| CmGSTU13 | Unnamed__4 | CTCC | 594 | 4 | - |
| CmGSTU13 |  | motif_sequence | 103 | 4 | - |
| CmGSTU13 |  | motif_sequence | 167 | 4 | + |
| CmGSTU13 |  | motif_sequence | 355 | 4 | - |
| CmGSTU13 |  | motif_sequence | 459 | 4 | - |
| CmGSTU13 | CAAT-box | CCAAT | 60 | 5 | - |
| CmGSTU13 | CAAT-box | CCAAT | 84 | 5 | + |
| CmGSTU13 | CAAT-box | CAAT | 85 | 4 | + |
| CmGSTU13 | CAAT-box | CCAAT | 90 | 5 | + |
| CmGSTU13 | CAAT-box | CAAT | 91 | 4 | + |
| CmGSTU13 | CAAT-box | CAAT | 97 | 4 | - |
| CmGSTU13 | CAAT-box | CAAAT | 128 | 5 | - |
| CmGSTU13 | CAAT-box | CCCAATTT | 147 | 8 | + |
| CmGSTU13 | CAAT-box | CCAAT | 148 | 5 | + |
| CmGSTU13 | CAAT-box | CAAT | 149 | 4 | + |
| CmGSTU13 | CAAT-box | CAAT | 223 | 4 | - |
| CmGSTU13 | CAAT-box | CAAT | 263 | 4 | - |
| CmGSTU13 | CAAT-box | CCAAT | 393 | 5 | - |
| CmGSTU13 | CAAT-box | CAAAT | 426 | 5 | + |
| CmGSTU13 | CAAT-box | CCCAATTT | 446 | 8 | - |
| CmGSTU13 | CAAT-box | CCAAT | 448 | 5 | - |
| CmGSTU13 | CAAT-box | CAAT | 463 | 4 | - |
| CmGSTU13 | CAAT-box | CAAT | 517 | 4 | - |
| CmGSTU13 | CAAT-box | CAAAT | 537 | 5 | + |
| CmGSTU13 | CAAT-box | CAAT | 562 | 4 | - |
| CmGSTU13 | CAAT-box | TGCCAAC | 573 | 7 | + |
| CmGSTU13 | CAAT-box | CAAT | 582 | 4 | + |
| CmGSTU13 | ERE | ATTTTAAA | 629 | 8 | + |
| CmGSTU13 | P-box | CCTTTTG | 482 | 7 | + |
| CmGSTU13 | MRE | AACCTAA | 547 | 7 | + |
| CmGSTU13 | TATA-box | TATA | 301 | 4 | + |
| CmGSTU14 | TATA-box | TATA | 49 | 4 | + |
| CmGSTU14 | TATA-box | TATA | 313 | 4 | + |
| CmGSTU14 | TATA-box | TATA | 583 | 4 | - |
| CmGSTU14 | MYC | CATTTG | 567 | 6 | - |
| CmGSTU14 | MRE | AACCTAA | 559 | 7 | + |
| CmGSTU14 | CAAT-box | CAAT | 103 | 4 | - |
| CmGSTU14 | CAAT-box | CAAAT | 137 | 5 | - |
| CmGSTU14 | CAAT-box | CCCAATTT | 156 | 8 | + |
| CmGSTU14 | CAAT-box | CCAAT | 157 | 5 | + |
| CmGSTU14 | CAAT-box | CAAT | 158 | 4 | + |
| CmGSTU14 | CAAT-box | CAAT | 189 | 4 | + |
| CmGSTU14 | CAAT-box | CAAT | 232 | 4 | - |
| CmGSTU14 | CAAT-box | CAAT | 316 | 4 | - |
| CmGSTU14 | CAAT-box | CAAT | 360 | 4 | + |
| CmGSTU14 | CAAT-box | CCAAT | 460 | 5 | - |
| CmGSTU14 | CAAT-box | CAAAT | 465 | 5 | - |
| CmGSTU14 | CAAT-box | CAAT | 475 | 4 | - |
| CmGSTU14 | CAAT-box | CAAAT | 549 | 5 | + |
| CmGSTU14 | CAAT-box | CAAAT | 567 | 5 | + |
| CmGSTU14 | Unnamed__1 | CGTGG | 87 | 5 | + |
| CmGSTU14 | TGACG-motif | TGACG | 105 | 5 | + |
| CmGSTU14 | TCA | TCATCTTCAT | 362 | 9 | - |
| CmGSTU14 | CARE | CAACTCAC | 542 | 8 | + |
| CmGSTU14 | Unnamed__4 | CTCC | 7 | 4 | - |
| CmGSTU14 | Unnamed__4 | CTCC | 56 | 4 | - |
| CmGSTU14 | Unnamed__4 | CTCC | 63 | 4 | - |
| CmGSTU14 | Unnamed__4 | CTCC | 448 | 4 | - |
| CmGSTU14 | Unnamed__4 | CTCC | 451 | 4 | - |
| CmGSTU14 | ARE | AAACCA | 30 | 6 | - |
| CmGSTU14 | ARE | AAACCA | 485 | 6 | - |
| CmGSTU14 | AE-box | AGAAACTT | 237 | 8 | + |
| CmGSTU14 | GATA-motif | GATAGGG | 277 | 7 | - |
| CmGSTU14 | CGTCA-motif | CGTCA | 105 | 5 | - |
| CmGSTU14 |  | motif_sequence | 10 | 4 | - |
| CmGSTU14 |  | motif_sequence | 56 | 4 | - |
| CmGSTU14 |  | motif_sequence | 109 | 4 | - |
| CmGSTU14 |  | motif_sequence | 176 | 4 | + |
| CmGSTU14 |  | motif_sequence | 378 | 4 | - |
| CmGSTU14 | O2-site | GATGACATGG | 319 | 9 | + |
| CmGSTU14 | as-1 | TGACG | 105 | 5 | + |
| CmGSTU15 |  | motif_sequence | 112 | 4 | - |
| CmGSTU15 |  | motif_sequence | 176 | 4 | + |
| CmGSTU15 |  | motif_sequence | 378 | 4 | - |
| CmGSTU15 |  | motif_sequence | 394 | 4 | - |
| CmGSTU15 |  | motif_sequence | 626 | 4 | + |
| CmGSTU15 | as-1 | TGACG | 108 | 5 | + |
| CmGSTU15 | GATA-motif | GATAGGG | 277 | 7 | - |
| CmGSTU15 | TGA-element | AACGAC | 91 | 6 | - |
| CmGSTU15 | ARE | AAACCA | 33 | 6 | - |
| CmGSTU15 | TCA-element | CCATCTTTTT | 620 | 9 | - |
| CmGSTU15 | AE-box | AGAAACTT | 237 | 8 | + |
| CmGSTU15 | CGTCA-motif | CGTCA | 108 | 5 | - |
| CmGSTU15 | Unnamed__4 | CTCC | 7 | 4 | - |
| CmGSTU15 | Unnamed__4 | CTCC | 448 | 4 | - |
| CmGSTU15 | Unnamed__4 | CTCC | 451 | 4 | - |
| CmGSTU15 | Unnamed__4 | CTCC | 572 | 4 | - |
| CmGSTU15 | TATA-box | TATA | 52 | 4 | + |
| CmGSTU15 | TATA-box | ATTATA | 254 | 6 | + |
| CmGSTU15 | TATA-box | TATAA | 255 | 5 | - |
| CmGSTU15 | TATA-box | TATA | 256 | 4 | + |
| CmGSTU15 | TATA-box | TATA | 313 | 4 | + |
| CmGSTU15 | TATA-box | TACAAAA | 341 | 7 | + |
| CmGSTU15 | TATA-box | TACAAAA | 663 | 7 | + |
| CmGSTU15 | MYC | CAATTG | 458 | 6 | - |
| CmGSTU15 | TGACG-motif | TGACG | 108 | 5 | + |
| CmGSTU15 | CAAT-box | CCAAT | 69 | 5 | - |
| CmGSTU15 | CAAT-box | CAAT | 106 | 4 | - |
| CmGSTU15 | CAAT-box | CAAAT | 116 | 5 | - |
| CmGSTU15 | CAAT-box | CAAAT | 137 | 5 | - |
| CmGSTU15 | CAAT-box | CAAT | 316 | 4 | - |
| CmGSTU15 | CAAT-box | CAAT | 458 | 4 | + |
| CmGSTU15 | CAAT-box | CCAAT | 460 | 5 | - |
| CmGSTU15 | CAAT-box | CAAAT | 465 | 5 | - |
| CmGSTU15 | CAAT-box | CAAT | 475 | 4 | - |
| CmGSTU15 | CAAT-box | CAAAT | 557 | 5 | + |
| CmGSTU16 | CAAT-box | CAAT | 18 | 4 | - |
| CmGSTU16 | CAAT-box | CAAAT | 107 | 5 | - |
| CmGSTU16 | CAAT-box | CAAT | 141 | 4 | + |
| CmGSTU16 | CAAT-box | CCAAT | 145 | 5 | + |
| CmGSTU16 | CAAT-box | CAAT | 146 | 4 | + |
| CmGSTU16 | CAAT-box | CCAAT | 454 | 5 | - |
| CmGSTU16 | CAAT-box | CAAAT | 459 | 5 | - |
| CmGSTU16 | CAAT-box | CAAT | 469 | 4 | - |
| CmGSTU16 | CAAT-box | CAAT | 484 | 4 | - |
| CmGSTU16 | CAAT-box | CCAAT | 491 | 5 | - |
| CmGSTU16 | CAAT-box | CAAAT | 522 | 5 | - |
| CmGSTU16 | CAAT-box | CAAAT | 551 | 5 | + |
| CmGSTU16 | CAAT-box | CAAT | 577 | 4 | - |
| CmGSTU16 | TATA-box | TATATTTATATTT | 85 | 12 | - |
| CmGSTU16 | TATA-box | ATTATA | 86 | 6 | + |
| CmGSTU16 | TATA-box | TATAA | 87 | 5 | - |
| CmGSTU16 | TATA-box | TATA | 88 | 4 | + |
| CmGSTU16 | TATA-box | ATATAA | 93 | 6 | + |
| CmGSTU16 | TATA-box | TATA | 94 | 4 | + |
| CmGSTU16 | TATA-box | TATAA | 203 | 5 | - |
| CmGSTU16 | TATA-box | TATA | 204 | 4 | + |
| CmGSTU16 | TATA-box | TATATA | 304 | 6 | + |
| CmGSTU16 | TATA-box | TATA | 306 | 4 | + |
| CmGSTU16 | MYB recognition site | CCGTTG | 177 | 6 | - |
| CmGSTU16 | MYB recognition site | CCGTTG | 503 | 6 | + |
| CmGSTU16 | P-box | CCTTTTG | 129 | 7 | + |
| CmGSTU16 | MYB | CAACCA | 653 | 6 | + |
| CmGSTU16 | CCAAT-box | CAACGG | 177 | 6 | + |
| CmGSTU16 | CCAAT-box | CAACGG | 503 | 6 | - |
| CmGSTU16 | Unnamed__4 | CTCC | 50 | 4 | - |
| CmGSTU16 | Unnamed__4 | CTCC | 57 | 4 | - |
| CmGSTU16 | Unnamed__4 | CTCC | 393 | 4 | - |
| CmGSTU16 | Unnamed__4 | CTCC | 442 | 4 | - |
| CmGSTU16 | Unnamed__4 | CTCC | 566 | 4 | - |
| CmGSTU16 | W box | TTGACC | 594 | 6 | - |
| CmGSTU16 | AAGAA-motif | GAAAGAA | 250 | 7 | - |
| CmGSTU16 | AT~TATA-box | TATATA | 304 | 6 | + |
| CmGSTU16 | ARE | AAACCA | 582 | 6 | + |
| CmGSTU16 | O2-site | GATGACATGG | 310 | 9 | + |
| CmGSTU16 |  | motif_sequence | 50 | 4 | - |
| CmGSTU16 |  | motif_sequence | 103 | 4 | - |
| CmGSTU16 |  | motif_sequence | 361 | 4 | - |
| CmGSTU16 |  | motif_sequence | 465 | 4 | - |
| CmGSTU16 |  | motif_sequence | 571 | 4 | - |
| CmGSTU17 | TATA-box | TATA | 110 | 4 | + |
| CmGSTU17 | Box 4 | ATTAAT | 330 | 6 | + |
| CmGSTU17 | Unnamed__1 | CGTGG | 12 | 5 | + |
| CmGSTU17 | ERE | ATTTCATA | 479 | 8 | + |
| CmGSTU17 | ABRE | ACGTG | 11 | 5 | + |
| CmGSTU17 | CAAT-box | CAAAT | 21 | 5 | - |
| CmGSTU17 | CAAT-box | CCAAT | 60 | 5 | - |
| CmGSTU17 | CAAT-box | CAAT | 72 | 4 | - |
| CmGSTU17 | CAAT-box | CAAT | 97 | 4 | - |
| CmGSTU17 | CAAT-box | CAAAT | 131 | 5 | - |
| CmGSTU17 | CAAT-box | CAAAT | 141 | 5 | + |
| CmGSTU17 | CAAT-box | CAAT | 208 | 4 | - |
| CmGSTU17 | CAAT-box | CAAT | 220 | 4 | + |
| CmGSTU17 | CAAT-box | CAAAT | 235 | 5 | - |
| CmGSTU17 | CAAT-box | CAAT | 246 | 4 | + |
| CmGSTU17 | CAAT-box | CAAT | 377 | 4 | + |
| CmGSTU17 | CAAT-box | CCCAATTT | 451 | 8 | - |
| CmGSTU17 | CAAT-box | CCAAT | 453 | 5 | - |
| CmGSTU17 | CAAT-box | CAAAT | 459 | 5 | - |
| CmGSTU17 | CAAT-box | CAAT | 469 | 4 | - |
| CmGSTU17 | CAAT-box | CCAAT | 491 | 5 | - |
| CmGSTU17 | CAAT-box | CAAT | 543 | 4 | - |
| CmGSTU17 | CAAT-box | CAAT | 568 | 4 | + |
| CmGSTU17 | CAAT-box | CAAT | 598 | 4 | - |
| CmGSTU17 | CAAT-box | CAAT | 609 | 4 | + |
| CmGSTU17 | CAAT-box | CAAT | 652 | 4 | + |
| CmGSTU17 | CAAT-box | CAAAT | 654 | 5 | - |
| CmGSTU17 |  | motif_sequence | 123 | 4 | - |
| CmGSTU17 |  | motif_sequence | 170 | 4 | + |
| CmGSTU17 |  | motif_sequence | 199 | 4 | - |
| CmGSTU17 |  | motif_sequence | 344 | 4 | - |
| CmGSTU17 |  | motif_sequence | 370 | 4 | - |
| CmGSTU17 |  | motif_sequence | 415 | 4 | - |
| CmGSTU17 |  | motif_sequence | 445 | 4 | - |
| CmGSTU17 |  | motif_sequence | 487 | 4 | - |
| CmGSTU17 |  | motif_sequence | 611 | 4 | + |
| CmGSTU17 | Unnamed__4 | CTCC | 4 | 4 | - |
| CmGSTU17 | Unnamed__4 | CTCC | 15 | 4 | - |
| CmGSTU17 | Unnamed__4 | CTCC | 184 | 4 | - |
| CmGSTU17 | Unnamed__4 | CTCC | 344 | 4 | - |
| CmGSTU17 | Unnamed__4 | CTCC | 442 | 4 | - |
| CmGSTU17 | Unnamed__4 | CTCC | 445 | 4 | - |
| CmGSTU17 | Unnamed__4 | CTCC | 494 | 4 | - |
| CmGSTU17 | GT1-motif | GGTTAA | 398 | 6 | + |
| CmGSTU17 | AP-1 | TGAGTTAG | 562 | 8 | - |
| CmGSTU17 | O2-site | GATGACATGG | 310 | 9 | + |
| CmGSTU17 | G-Box | CACGTT | 10 | 6 | - |
| CmGSTU18 | CAAT-box | CAAT | 24 | 4 | - |
| CmGSTU18 | CAAT-box | CAAT | 147 | 4 | + |
| CmGSTU18 | CAAT-box | CCAAT | 151 | 5 | + |
| CmGSTU18 | CAAT-box | CAAT | 152 | 4 | + |
| CmGSTU18 | CAAT-box | CCAAT | 405 | 5 | - |
| CmGSTU18 | CAAT-box | CCAAT | 460 | 5 | - |
| CmGSTU18 | CAAT-box | CAAAT | 465 | 5 | - |
| CmGSTU18 | CAAT-box | CAAT | 475 | 4 | - |
| CmGSTU18 | CAAT-box | CAAT | 490 | 4 | - |
| CmGSTU18 | CAAT-box | CCAAT | 497 | 5 | - |
| CmGSTU18 | CAAT-box | CAAT | 511 | 4 | - |
| CmGSTU18 | CAAT-box | CAAAT | 528 | 5 | - |
| CmGSTU18 | CAAT-box | CAAAT | 557 | 5 | + |
| CmGSTU18 | CAAT-box | CAAT | 583 | 4 | - |
| CmGSTU18 | MYB | CAACCA | 659 | 6 | + |
| CmGSTU18 | MYB recognition site | CCGTTG | 183 | 6 | - |
| CmGSTU18 | TATA-box | TATATTTATATTT | 91 | 12 | - |
| CmGSTU18 | TATA-box | ATTATA | 92 | 6 | + |
| CmGSTU18 | TATA-box | TATAA | 93 | 5 | - |
| CmGSTU18 | TATA-box | TATA | 94 | 4 | + |
| CmGSTU18 | TATA-box | ATATAA | 99 | 6 | + |
| CmGSTU18 | TATA-box | TATA | 100 | 4 | + |
| CmGSTU18 | TATA-box | TATAA | 209 | 5 | - |
| CmGSTU18 | TATA-box | TATA | 210 | 4 | + |
| CmGSTU18 | TATA-box | TATATA | 310 | 6 | + |
| CmGSTU18 | TATA-box | TATA | 312 | 4 | + |
| CmGSTU18 | TATA-box | TATA | 336 | 4 | - |
| CmGSTU18 | Unnamed__4 | CTCC | 63 | 4 | - |
| CmGSTU18 | Unnamed__4 | CTCC | 399 | 4 | - |
| CmGSTU18 | Unnamed__4 | CTCC | 448 | 4 | - |
| CmGSTU18 | Unnamed__4 | CTCC | 572 | 4 | - |
| CmGSTU18 | Unnamed__4 | CTCC | 617 | 4 | + |
| CmGSTU18 | CCAAT-box | CAACGG | 183 | 6 | + |
| CmGSTU18 | AAGAA-motif | GAAAGAA | 256 | 7 | - |
| CmGSTU18 | W box | TTGACC | 600 | 6 | - |
| CmGSTU18 | AT~TATA-box | TATATA | 310 | 6 | + |
| CmGSTU18 | ARE | AAACCA | 588 | 6 | + |
| CmGSTU18 | O2-site | GATGACATGG | 316 | 9 | + |
| CmGSTU18 |  | motif_sequence | 56 | 4 | - |
| CmGSTU18 |  | motif_sequence | 367 | 4 | - |
| CmGSTU18 |  | motif_sequence | 471 | 4 | - |
| CmGSTU18 |  | motif_sequence | 577 | 4 | - |
| CmGSTU19 | circadian | CAAAGATATC | 438 | 9 | + |
| CmGSTU19 | circadian | CAAAGATATC | 442 | 9 | - |
| CmGSTU19 | ATCT-motif | AATCTAATCC | 152 | 9 | + |
| CmGSTU19 | TCA-element | TCAGAAGAGG | 4 | 9 | + |
| CmGSTU19 | TCT-motif | TCTTAC | 185 | 6 | + |
| CmGSTU19 | LAMP-element | CTTTATCA | 636 | 8 | + |
| CmGSTU19 | GT1-motif | GGTTAA | 57 | 6 | + |
| CmGSTU19 | WRE3 | CCACCT | 126 | 6 | - |
| CmGSTU19 |  | motif_sequence | 30 | 4 | + |
| CmGSTU19 |  | motif_sequence | 103 | 4 | - |
| CmGSTU19 |  | motif_sequence | 179 | 4 | + |
| CmGSTU19 |  | motif_sequence | 370 | 4 | - |
| CmGSTU19 | CAAT-box | CAAT | 51 | 4 | + |
| CmGSTU19 | CAAT-box | CAAT | 64 | 4 | - |
| CmGSTU19 | CAAT-box | CAAAT | 110 | 5 | - |
| CmGSTU19 | CAAT-box | CAAAT | 150 | 5 | + |
| CmGSTU19 | CAAT-box | CAAT | 204 | 4 | - |
| CmGSTU19 | CAAT-box | CAAT | 223 | 4 | - |
| CmGSTU19 | CAAT-box | CAAT | 235 | 4 | - |
| CmGSTU19 | CAAT-box | CAAT | 310 | 4 | - |
| CmGSTU19 | CAAT-box | CAAAT | 333 | 5 | - |
| CmGSTU19 | CAAT-box | CAAAT | 352 | 5 | - |
| CmGSTU19 | CAAT-box | CAAT | 415 | 4 | + |
| CmGSTU19 | CAAT-box | CCAAT | 417 | 5 | - |
| CmGSTU19 | CAAT-box | CAAT | 469 | 4 | - |
| CmGSTU19 | CAAT-box | CAAT | 485 | 4 | + |
| CmGSTU19 | Myc | TCTCTTA | 183 | 7 | + |
| CmGSTU19 | TATA-box | TATAA | 17 | 5 | - |
| CmGSTU19 | TATA-box | TATA | 18 | 4 | + |
| CmGSTU19 | TATA-box | TATAAATA | 162 | 8 | - |
| CmGSTU19 | TATA-box | TATAAAT | 163 | 7 | - |
| CmGSTU19 | TATA-box | TATAAA | 164 | 6 | - |
| CmGSTU19 | TATA-box | TATAA | 165 | 5 | - |
| CmGSTU19 | TATA-box | TATA | 166 | 4 | + |
| CmGSTU19 | TATA-box | TATA | 232 | 4 | + |
| CmGSTU19 | TATA-box | TATAAAT | 628 | 7 | - |
| CmGSTU19 | TATA-box | TATAAA | 629 | 6 | - |
| CmGSTU19 | TATA-box | TATAA | 630 | 5 | - |
| CmGSTU19 | TATA-box | TATA | 631 | 4 | - |
| CmGSTU19 | STRE | AGGGG | 432 | 5 | + |
| CmGSTU19 | MYB recognition site | CCGTTG | 363 | 6 | - |
| CmGSTU19 | MYC | CAATTG | 415 | 6 | - |
| CmGSTU19 | P-box | CCTTTTG | 586 | 7 | - |
| CmGSTU19 | CCAAT-box | CAACGG | 363 | 6 | + |
| CmGSTU19 | TC-rich repeats | GTTTTCTTAC | 181 | 9 | + |
| CmGSTU19 | Unnamed__4 | CTCC | 12 | 4 | - |
| CmGSTU19 | Unnamed__4 | CTCC | 82 | 4 | - |
| CmGSTU19 | Unnamed__4 | CTCC | 216 | 4 | + |
| CmGSTU19 | Unnamed__4 | CTCC | 356 | 4 | - |
| CmGSTU19 | Unnamed__4 | CTCC | 367 | 4 | - |
| CmGSTU19 | Unnamed__4 | CTCC | 451 | 4 | - |
| CmGSTU19 | Unnamed__4 | CTCC | 645 | 4 | + |
| CmGSTU19 | CARE | CAACTCAC | 137 | 8 | - |
| CmGSTU19 | AAGAA-motif | GAAAGAA | 653 | 7 | + |
| CmGSTU2 | MYB | CAACAG | 275 | 6 | - |
| CmGSTU2 | box S | AGCCACC | 470 | 7 | - |
| CmGSTU2 | MYC | CATTTG | 397 | 6 | + |
| CmGSTU2 | MYC | CATTTG | 552 | 6 | - |
| CmGSTU2 | TATA-box | TATA | 324 | 4 | + |
| CmGSTU2 | STRE | AGGGG | 435 | 5 | + |
| CmGSTU2 | STRE | AGGGG | 541 | 5 | - |
| CmGSTU2 | CAAT-box | CAAT | 72 | 4 | - |
| CmGSTU2 | CAAT-box | CAAT | 126 | 4 | - |
| CmGSTU2 | CAAT-box | CAAT | 141 | 4 | + |
| CmGSTU2 | CAAT-box | CAAT | 220 | 4 | - |
| CmGSTU2 | CAAT-box | CAAT | 246 | 4 | - |
| CmGSTU2 | CAAT-box | CAAAT | 297 | 5 | - |
| CmGSTU2 | CAAT-box | CAAAT | 389 | 5 | - |
| CmGSTU2 | CAAT-box | CAAAT | 398 | 5 | - |
| CmGSTU2 | CAAT-box | CCAAT | 445 | 5 | - |
| CmGSTU2 | CAAT-box | CAAT | 460 | 4 | - |
| CmGSTU2 | CAAT-box | CAAT | 519 | 4 | - |
| CmGSTU2 | CAAT-box | CAAAT | 552 | 5 | + |
| CmGSTU2 | CAAT-box | CAAAT | 567 | 5 | - |
| CmGSTU2 | CAAT-box | CCAAT | 580 | 5 | + |
| CmGSTU2 | CAAT-box | CAAT | 581 | 4 | + |
| CmGSTU2 | CAAT-box | CAAT | 588 | 4 | + |
| CmGSTU2 | CAAT-box | CAAT | 636 | 4 | - |
| CmGSTU2 | Myb | TAACTG | 524 | 6 | + |
| CmGSTU2 |  | motif_sequence | 7 | 4 | - |
| CmGSTU2 |  | motif_sequence | 19 | 4 | + |
| CmGSTU2 |  | motif_sequence | 26 | 4 | - |
| CmGSTU2 |  | motif_sequence | 103 | 4 | - |
| CmGSTU2 |  | motif_sequence | 164 | 4 | + |
| CmGSTU2 |  | motif_sequence | 406 | 4 | - |
| CmGSTU2 | 3-AF1 binding site | TAAGAGAGGAA | 356 | 10 | + |
| CmGSTU2 | 3-AF1 binding site | TAAGAGAGGAA | 494 | 10 | + |
| CmGSTU2 | 3-AF1 binding site | TAAGAGAGGAA | 539 | 10 | - |
| CmGSTU2 | Myb-binding site | CAACAG | 275 | 6 | - |
| CmGSTU2 | Unnamed__4 | CTCC | 3 | 4 | - |
| CmGSTU2 | Unnamed__4 | CTCC | 135 | 4 | - |
| CmGSTU2 | Unnamed__4 | CTCC | 384 | 4 | - |
| CmGSTU2 | Unnamed__4 | CTCC | 433 | 4 | - |
| CmGSTU2 | Unnamed__4 | CTCC | 438 | 4 | - |
| CmGSTU2 | Unnamed__4 | CTCC | 578 | 4 | + |
| CmGSTU2 | O2-site | GATGACATGG | 304 | 9 | + |
| CmGSTU2 | WRE3 | CCACCT | 601 | 6 | + |
| CmGSTU20 |  | motif_sequence | 35 | 4 | - |
| CmGSTU20 |  | motif_sequence | 112 | 4 | - |
| CmGSTU20 |  | motif_sequence | 173 | 4 | + |
| CmGSTU20 |  | motif_sequence | 232 | 4 | - |
| CmGSTU20 |  | motif_sequence | 415 | 4 | - |
| CmGSTU20 |  | motif_sequence | 538 | 4 | - |
| CmGSTU20 |  | motif_sequence | 598 | 4 | - |
| CmGSTU20 |  | motif_sequence | 620 | 4 | + |
| CmGSTU20 |  | motif_sequence | 660 | 4 | - |
| CmGSTU20 | WUN-motif | CAATTACAT | 542 | 9 | - |
| CmGSTU20 | Myb-binding site | CAACAG | 380 | 6 | - |
| CmGSTU20 | Unnamed__4 | CTCC | 361 | 4 | - |
| CmGSTU20 | Unnamed__4 | CTCC | 387 | 4 | - |
| CmGSTU20 | Unnamed__4 | CTCC | 538 | 4 | - |
| CmGSTU20 | Y-box | TGTGGAGGAGCA | 381 | 11 | + |
| CmGSTU20 | MYB | CAACAG | 380 | 6 | - |
| CmGSTU20 | I-box | GATAAGGGT | 402 | 9 | + |
| CmGSTU20 | MYC | CATGTG | 576 | 6 | - |
| CmGSTU20 | TATA-box | TATA | 43 | 4 | + |
| CmGSTU20 | CAAT-box | CAAT | 64 | 4 | - |
| CmGSTU20 | CAAT-box | CAAT | 106 | 4 | - |
| CmGSTU20 | CAAT-box | CAAT | 135 | 4 | - |
| CmGSTU20 | CAAT-box | CAAT | 150 | 4 | + |
| CmGSTU20 | CAAT-box | CAAT | 199 | 4 | - |
| CmGSTU20 | CAAT-box | CAAT | 238 | 4 | + |
| CmGSTU20 | CAAT-box | CAAT | 307 | 4 | + |
| CmGSTU20 | CAAT-box | CAAAT | 351 | 5 | + |
| CmGSTU20 | CAAT-box | CAAT | 391 | 4 | + |
| CmGSTU20 | CAAT-box | CAAT | 399 | 4 | - |
| CmGSTU20 | CAAT-box | CAAAT | 411 | 5 | - |
| CmGSTU20 | CAAT-box | CAAAT | 515 | 5 | - |
| CmGSTU20 | CAAT-box | CAAT | 547 | 4 | - |
| CmGSTU20 | circadian | CAAAGATATC | 484 | 9 | - |
| CmGSTU21 | circadian | CAAAGATATC | 81 | 9 | + |
| CmGSTU21 | CAAT-box | CAAT | 33 | 4 | - |
| CmGSTU21 | CAAT-box | CAAAT | 141 | 5 | + |
| CmGSTU21 | CAAT-box | CAAT | 147 | 4 | + |
| CmGSTU21 | CAAT-box | CAAT | 183 | 4 | + |
| CmGSTU21 | CAAT-box | CAAAT | 196 | 5 | - |
| CmGSTU21 | CAAT-box | CAAT | 246 | 4 | + |
| CmGSTU21 | CAAT-box | CAAT | 341 | 4 | + |
| CmGSTU21 | CAAT-box | CCAAT | 346 | 5 | - |
| CmGSTU21 | CAAT-box | CAAT | 365 | 4 | - |
| CmGSTU21 | CAAT-box | CCAAT | 378 | 5 | + |
| CmGSTU21 | CAAT-box | CAAT | 379 | 4 | + |
| CmGSTU21 | TATA | TATAAAAT | 333 | 8 | - |
| CmGSTU21 | TATA-box | TACAAAA | 48 | 7 | - |
| CmGSTU21 | TATA-box | TATAAA | 155 | 6 | - |
| CmGSTU21 | TATA-box | TATAA | 156 | 5 | - |
| CmGSTU21 | TATA-box | TATA | 157 | 4 | + |
| CmGSTU21 | TATA-box | TATA | 210 | 4 | - |
| CmGSTU21 | TATA-box | ATATAT | 329 | 6 | - |
| CmGSTU21 | TATA-box | TATA | 330 | 4 | - |
| CmGSTU21 | TATA-box | TATAAAA | 334 | 7 | - |
| CmGSTU21 | TATA-box | TATAAA | 335 | 6 | - |
| CmGSTU21 | TATA-box | TATAA | 336 | 5 | - |
| CmGSTU21 | TATA-box | TATA | 337 | 4 | - |
| CmGSTU21 | TGA-element | AACGAC | 241 | 6 | + |
| CmGSTU21 | MYC | CATTTG | 195 | 6 | + |
| CmGSTU21 | HD-Zip 1 | CAAT(A/T)ATTG | 341 | 8.5 | - |
| CmGSTU21 | P-box | CCTTTTG | 46 | 7 | + |
| CmGSTU21 | P-box | CCTTTTG | 91 | 7 | + |
| CmGSTU21 | SARE | TTCGACCATCTT | 55 | 11 | - |
| CmGSTU21 | Unnamed__4 | CTCC | 132 | 4 | - |
| CmGSTU21 |  | motif_sequence | 109 | 4 | - |
| CmGSTU21 |  | motif_sequence | 170 | 4 | + |
| CmGSTU21 |  | motif_sequence | 188 | 4 | - |
| CmGSTU21 |  | motif_sequence | 222 | 4 | - |
| CmGSTU21 |  | motif_sequence | 257 | 4 | + |
| CmGSTU21 |  | motif_sequence | 325 | 4 | - |
| CmGSTU22 | CAAT-box | CAAAT | 141 | 5 | + |
| CmGSTU22 | CAAT-box | CAAT | 144 | 4 | - |
| CmGSTU22 | CAAT-box | CAAT | 183 | 4 | + |
| CmGSTU22 | CAAT-box | CAAAT | 196 | 5 | - |
| CmGSTU22 | CAAT-box | CAAT | 246 | 4 | + |
| CmGSTU22 | CAAT-box | CAAT | 295 | 4 | + |
| CmGSTU22 | CAAT-box | CAAT | 374 | 4 | + |
| CmGSTU22 | CAAT-box | CAAT | 397 | 4 | + |
| CmGSTU22 | CAAT-box | CAAT | 423 | 4 | + |
| CmGSTU22 | CAAT-box | CAAT | 499 | 4 | + |
| CmGSTU22 | CAAT-box | CCAAT | 506 | 5 | + |
| CmGSTU22 | CAAT-box | CAAT | 507 | 4 | + |
| CmGSTU22 | CAAT-box | CAAT | 509 | 4 | - |
| CmGSTU22 | CAAT-box | CAAT | 532 | 4 | - |
| CmGSTU22 | CAAT-box | CAAT | 546 | 4 | + |
| CmGSTU22 | CAAT-box | CAAT | 602 | 4 | + |
| CmGSTU22 | STRE | AGGGG | 468 | 5 | - |
| CmGSTU22 | TATA-box | TACAAAA | 48 | 7 | - |
| CmGSTU22 | TATA-box | ccTATAAAaa | 153 | 9 | - |
| CmGSTU22 | TATA-box | TATAAA | 155 | 6 | - |
| CmGSTU22 | TATA-box | TATAA | 156 | 5 | - |
| CmGSTU22 | TATA-box | TATA | 157 | 4 | + |
| CmGSTU22 | TATA-box | TATA | 210 | 4 | + |
| CmGSTU22 | TATA-box | TACAAAA | 475 | 7 | - |
| CmGSTU22 | MYC | CATTTG | 195 | 6 | + |
| CmGSTU22 | MYC | CAATTG | 507 | 6 | - |
| CmGSTU22 | P-box | CCTTTTG | 46 | 7 | + |
| CmGSTU22 | P-box | CCTTTTG | 91 | 7 | + |
| CmGSTU22 | P-box | CCTTTTG | 473 | 7 | + |
| CmGSTU22 | MYB | CAACCA | 521 | 6 | - |
| CmGSTU22 | SARE | TTCGACCATCTT | 55 | 11 | - |
| CmGSTU22 | TATC-box | TATCCCA | 461 | 7 | - |
| CmGSTU22 | Unnamed__4 | CTCC | 132 | 4 | - |
| CmGSTU22 | Unnamed__4 | CTCC | 342 | 4 | + |
| CmGSTU22 | Unnamed__4 | CTCC | 433 | 4 | - |
| CmGSTU22 | Unnamed__4 | CTCC | 471 | 4 | + |
| CmGSTU22 | Unnamed__4 | CTCC | 556 | 4 | - |
| CmGSTU22 | GCN4_motif | TGAGTCA | 534 | 7 | + |
| CmGSTU22 | W box | TTGACC | 524 | 6 | + |
| CmGSTU22 | AAGAA-motif | GAAAGAA | 482 | 7 | + |
| CmGSTU22 | LAMP-element | CTTTATCA | 417 | 8 | + |
| CmGSTU22 |  | motif_sequence | 109 | 4 | - |
| CmGSTU22 |  | motif_sequence | 170 | 4 | + |
| CmGSTU22 |  | motif_sequence | 188 | 4 | - |
| CmGSTU22 |  | motif_sequence | 222 | 4 | - |
| CmGSTU22 |  | motif_sequence | 257 | 4 | + |
| CmGSTU22 |  | motif_sequence | 428 | 4 | - |
| CmGSTU22 |  | motif_sequence | 486 | 4 | - |
| CmGSTU22 |  | motif_sequence | 491 | 4 | - |
| CmGSTU22 |  | motif_sequence | 556 | 4 | - |
| CmGSTU23 | STRE | AGGGG | 36 | 5 | + |
| CmGSTU23 | STRE | AGGGG | 98 | 5 | + |
| CmGSTU23 | MYC | CATTTG | 131 | 6 | + |
| CmGSTU23 | MYB | CAACCA | 262 | 6 | - |
| CmGSTU23 | CAAT-box | CAAT | 58 | 4 | - |
| CmGSTU23 | CAAT-box | CCAAT | 114 | 5 | - |
| CmGSTU23 | CAAT-box | CAAAT | 132 | 5 | - |
| CmGSTU23 | CAAT-box | CAAAT | 158 | 5 | - |
| CmGSTU23 | CAAT-box | CAAT | 226 | 4 | - |
| CmGSTU23 | CAAT-box | CAAT | 273 | 4 | - |
| CmGSTU23 | CAAT-box | CAAT | 277 | 4 | - |
| CmGSTU23 | MBS | CAACTG | 248 | 6 | - |
| CmGSTU23 |  | motif_sequence | 61 | 4 | - |
| CmGSTU23 |  | motif_sequence | 94 | 4 | - |
| CmGSTU23 |  | motif_sequence | 118 | 4 | - |
| CmGSTU23 |  | motif_sequence | 128 | 4 | - |
| CmGSTU23 |  | motif_sequence | 162 | 4 | - |
| CmGSTU23 |  | motif_sequence | 190 | 4 | + |
| CmGSTU23 |  | motif_sequence | 205 | 4 | - |
| CmGSTU23 |  | motif_sequence | 235 | 4 | - |
| CmGSTU23 |  | motif_sequence | 240 | 4 | - |
| CmGSTU23 |  | motif_sequence | 280 | 4 | - |
| CmGSTU23 | Myb | CAACTG | 248 | 6 | - |
| CmGSTU23 | Unnamed__4 | CTCC | 142 | 4 | - |
| CmGSTU23 | Unnamed__4 | CTCC | 217 | 4 | - |
| CmGSTU23 | Unnamed__4 | CTCC | 297 | 4 | - |
| CmGSTU23 | Unnamed__4 | CTCC | 305 | 4 | + |
| CmGSTU23 | Unnamed__4 | CTCC | 308 | 4 | + |
| CmGSTU24 | STRE | AGGGG | 36 | 5 | + |
| CmGSTU24 | STRE | AGGGG | 98 | 5 | + |
| CmGSTU24 | MYC | CATTTG | 131 | 6 | + |
| CmGSTU24 | TCA | TCATCTTCAT | 140 | 9 | - |
| CmGSTU24 |  | motif_sequence | 94 | 4 | - |
| CmGSTU24 |  | motif_sequence | 105 | 4 | - |
| CmGSTU24 |  | motif_sequence | 118 | 4 | - |
| CmGSTU24 |  | motif_sequence | 142 | 4 | - |
| CmGSTU24 |  | motif_sequence | 205 | 4 | - |
| CmGSTU24 | CAAT-box | CAAT | 23 | 4 | + |
| CmGSTU24 | CAAT-box | CAAAT | 132 | 5 | - |
| CmGSTU24 | CAAT-box | CAAT | 179 | 4 | + |
| CmGSTU24 | Unnamed__4 | CTCC | 118 | 4 | - |
| CmGSTU24 | Unnamed__4 | CTCC | 142 | 4 | - |
| CmGSTU3 |  | motif_sequence | 4 | 4 | - |
| CmGSTU3 |  | motif_sequence | 42 | 4 | - |
| CmGSTU3 |  | motif_sequence | 143 | 4 | + |
| CmGSTU3 |  | motif_sequence | 149 | 4 | + |
| CmGSTU3 |  | motif_sequence | 346 | 4 | - |
| CmGSTU3 |  | motif_sequence | 353 | 4 | - |
| CmGSTU3 |  | motif_sequence | 391 | 4 | - |
| CmGSTU3 |  | motif_sequence | 496 | 4 | - |
| CmGSTU3 |  | motif_sequence | 571 | 4 | - |
| CmGSTU3 |  | motif_sequence | 709 | 4 | - |
| CmGSTU3 | CAAT-box | CAAT | 21 | 4 | - |
| CmGSTU3 | CAAT-box | CAAT | 66 | 4 | - |
| CmGSTU3 | CAAT-box | CAAT | 72 | 4 | - |
| CmGSTU3 | CAAT-box | CAAT | 123 | 4 | + |
| CmGSTU3 | CAAT-box | CAAAT | 217 | 5 | - |
| CmGSTU3 | CAAT-box | CAAT | 234 | 4 | - |
| CmGSTU3 | CAAT-box | CAAAT | 282 | 5 | + |
| CmGSTU3 | CAAT-box | CAAAT | 285 | 5 | - |
| CmGSTU3 | CAAT-box | CCAAT | 374 | 5 | - |
| CmGSTU3 | CAAT-box | CAAAT | 383 | 5 | - |
| CmGSTU3 | CAAT-box | CCAAT | 430 | 5 | - |
| CmGSTU3 | CAAT-box | CAAT | 445 | 4 | - |
| CmGSTU3 | CAAT-box | CAAT | 474 | 4 | + |
| CmGSTU3 | CAAT-box | CAAT | 511 | 4 | + |
| CmGSTU3 | CAAT-box | CAAT | 513 | 4 | - |
| CmGSTU3 | CAAT-box | CAAAT | 549 | 5 | + |
| CmGSTU3 | CAAT-box | CAAT | 578 | 4 | + |
| CmGSTU3 | CAAT-box | CAAT | 604 | 4 | - |
| CmGSTU3 | CAAT-box | CAAAT | 633 | 5 | - |
| CmGSTU3 | CAAT-box | CAAT | 642 | 4 | - |
| CmGSTU3 | CAAT-box | CAAAT | 673 | 5 | + |
| CmGSTU3 | CAAT-box | CAAT | 700 | 4 | + |
| CmGSTU3 | CAAT-box | CAAAT | 742 | 5 | + |
| CmGSTU3 | CAAT-box | CAAT | 748 | 4 | + |
| CmGSTU3 | CAAT-box | CAAAT | 769 | 5 | + |
| CmGSTU3 | CAAT-box | CCAAT | 878 | 5 | + |
| CmGSTU3 | CAAT-box | CAAT | 879 | 4 | + |
| CmGSTU3 | TATA-box | TATA | 312 | 4 | + |
| CmGSTU3 | TATA-box | TATA | 788 | 4 | - |
| CmGSTU3 | STRE | AGGGG | 420 | 5 | + |
| CmGSTU3 | STRE | AGGGG | 538 | 5 | - |
| CmGSTU3 | MYC | CATTTG | 382 | 6 | + |
| CmGSTU3 | MYC | CAATTG | 511 | 6 | - |
| CmGSTU3 | MYC | CATTTG | 549 | 6 | - |
| CmGSTU3 | MYC | CATTTG | 673 | 6 | - |
| CmGSTU3 | MYC | CATTTG | 769 | 6 | - |
| CmGSTU3 | MYB recognition site | CCGTTG | 263 | 6 | + |
| CmGSTU3 | P-box | CCTTTTG | 540 | 7 | + |
| CmGSTU3 | I-box | cCATATCCAAT | 763 | 10 | + |
| CmGSTU3 | MYB | CAACCA | 491 | 6 | - |
| CmGSTU3 | MYB | CAACCA | 760 | 6 | + |
| CmGSTU3 | CCAAT-box | CAACGG | 263 | 6 | - |
| CmGSTU3 | AuxRR-core | GGTCCAT | 503 | 7 | - |
| CmGSTU3 | Unnamed__4 | CTCC | 128 | 4 | + |
| CmGSTU3 | Unnamed__4 | CTCC | 418 | 4 | - |
| CmGSTU3 | Unnamed__4 | CTCC | 483 | 4 | - |
| CmGSTU3 | W box | TTGACC | 279 | 6 | - |
| CmGSTU4 | Unnamed__1 | CGTGG | 227 | 5 | + |
| CmGSTU4 | TGACG-motif | TGACG | 512 | 5 | + |
| CmGSTU4 | ABRE | GACACGTGGC | 223 | 9 | + |
| CmGSTU4 | ABRE | ACGTG | 226 | 5 | + |
| CmGSTU4 | CAAT-box | CAAT | 9 | 4 | - |
| CmGSTU4 | CAAT-box | CAAAT | 55 | 5 | - |
| CmGSTU4 | CAAT-box | CAAT | 94 | 4 | - |
| CmGSTU4 | CAAT-box | CAAAT | 114 | 5 | + |
| CmGSTU4 | CAAT-box | CAAT | 133 | 4 | + |
| CmGSTU4 | CAAT-box | CAAT | 138 | 4 | + |
| CmGSTU4 | CAAT-box | CAAAT | 291 | 5 | + |
| CmGSTU4 | CAAT-box | CAAT | 403 | 4 | + |
| CmGSTU4 | CAAT-box | CAAAT | 464 | 5 | - |
| CmGSTU4 | CAAT-box | CAAT | 481 | 4 | - |
| CmGSTU4 | CAAT-box | CCAAT | 488 | 5 | - |
| CmGSTU4 | CAAT-box | CAAT | 502 | 4 | - |
| CmGSTU4 | CAAT-box | CAAT | 525 | 4 | - |
| CmGSTU4 | CAAT-box | CAAT | 565 | 4 | - |
| CmGSTU4 | P-box | CCTTTTG | 126 | 7 | + |
| CmGSTU4 | MYB | CAACCA | 490 | 6 | - |
| CmGSTU4 | MYB | CAACCA | 558 | 6 | - |
| CmGSTU4 | MYB | CAACAG | 655 | 6 | - |
| CmGSTU4 | STRE | AGGGG | 241 | 5 | - |
| CmGSTU4 | TATA-box | ATATAT | 213 | 6 | + |
| CmGSTU4 | TATA-box | TATA | 214 | 4 | + |
| CmGSTU4 | MYC | CATTTG | 54 | 6 | + |
| CmGSTU4 | Unnamed__4 | CTCC | 16 | 4 | + |
| CmGSTU4 | Unnamed__4 | CTCC | 108 | 4 | + |
| CmGSTU4 | Unnamed__4 | CTCC | 124 | 4 | + |
| CmGSTU4 | Unnamed__4 | CTCC | 421 | 4 | - |
| CmGSTU4 | Unnamed__4 | CTCC | 439 | 4 | - |
| CmGSTU4 | Unnamed__4 | CTCC | 442 | 4 | - |
| CmGSTU4 | Unnamed__4 | CTCC | 517 | 4 | - |
| CmGSTU4 | Unnamed__4 | CTCC | 644 | 4 | - |
| CmGSTU4 | Unnamed__4 | CTCC | 648 | 4 | - |
| CmGSTU4 | Unnamed__4 | CTCC | 665 | 4 | + |
| CmGSTU4 | DRE core | GCCGAC | 535 | 6 | - |
| CmGSTU4 | Myb | TAACTG | 42 | 6 | - |
| CmGSTU4 | Myb | CAACTG | 652 | 6 | + |
| CmGSTU4 | MBS | CAACTG | 652 | 6 | + |
| CmGSTU4 | CAT-box | GCCACT | 271 | 6 | + |
| CmGSTU4 | CGTCA-motif | CGTCA | 512 | 5 | - |
| CmGSTU4 | LTR | CCGAAA | 667 | 6 | + |
| CmGSTU4 | GATA-motif | GATAGGG | 183 | 7 | - |
| CmGSTU4 | ARE | AAACCA | 317 | 6 | - |
| CmGSTU4 | as-1 | TGACG | 512 | 5 | + |
| CmGSTU4 | Myb-binding site | CAACAG | 655 | 6 | - |
| CmGSTU4 | G-Box | CACGTT | 225 | 6 | - |
| CmGSTU4 |  | motif_sequence | 100 | 4 | - |
| CmGSTU4 |  | motif_sequence | 106 | 4 | + |
| CmGSTU4 |  | motif_sequence | 117 | 4 | + |
| CmGSTU4 |  | motif_sequence | 122 | 4 | + |
| CmGSTU4 |  | motif_sequence | 153 | 4 | - |
| CmGSTU4 |  | motif_sequence | 421 | 4 | - |
| CmGSTU4 |  | motif_sequence | 442 | 4 | - |
| CmGSTU4 |  | motif_sequence | 602 | 4 | + |
| CmGSTU4 |  | motif_sequence | 663 | 4 | + |
| CmGSTU5 | CAAT-box | CAAAT | 55 | 5 | - |
| CmGSTU5 | CAAT-box | CAAAT | 114 | 5 | + |
| CmGSTU5 | CAAT-box | CAAT | 133 | 4 | + |
| CmGSTU5 | CAAT-box | CAAT | 138 | 4 | + |
| CmGSTU5 | CAAT-box | CAAAT | 291 | 5 | + |
| CmGSTU5 | CAAT-box | CAAAT | 461 | 5 | - |
| CmGSTU5 | CAAT-box | CAAAT | 533 | 5 | + |
| CmGSTU5 | CAAT-box | CAAT | 597 | 4 | + |
| CmGSTU5 | CAAT-box | CAAT | 654 | 4 | - |
| CmGSTU5 | CAAT-box | CAAAT | 658 | 5 | + |
| CmGSTU5 | CAAT-box | CCCAATTT | 659 | 8 | - |
| CmGSTU5 | CAAT-box | CCAAT | 661 | 5 | - |
| CmGSTU5 | MYB | CAACCA | 497 | 6 | - |
| CmGSTU5 | MYC | CATTTG | 54 | 6 | + |
| CmGSTU5 | TATA-box | ATATAA | 354 | 6 | + |
| CmGSTU5 | TATA-box | TATA | 355 | 4 | - |
| CmGSTU5 | STRE | AGGGG | 241 | 5 | - |
| CmGSTU5 | STRE | AGGGG | 345 | 5 | + |
| CmGSTU5 | Unnamed__4 | CTCC | 16 | 4 | + |
| CmGSTU5 | Unnamed__4 | CTCC | 119 | 4 | + |
| CmGSTU5 | Unnamed__4 | CTCC | 189 | 4 | + |
| CmGSTU5 | Unnamed__4 | CTCC | 208 | 4 | + |
| CmGSTU5 | Unnamed__4 | CTCC | 234 | 4 | - |
| CmGSTU5 | Unnamed__4 | CTCC | 252 | 4 | + |
| CmGSTU5 | Unnamed__4 | CTCC | 325 | 4 | - |
| CmGSTU5 | Unnamed__4 | CTCC | 564 | 4 | - |
| CmGSTU5 | Unnamed__4 | CTCC | 641 | 4 | - |
| CmGSTU5 | Unnamed__4 | CTCC | 645 | 4 | - |
| CmGSTU5 | Unnamed__4 | CTCC | 665 | 4 | - |
| CmGSTU5 | Myb | TAACTG | 42 | 6 | - |
| CmGSTU5 | LTR | CCGAAA | 571 | 6 | - |
| CmGSTU5 | G-box | CACGAC | 610 | 6 | + |
| CmGSTU5 | box S | AGCCACC | 485 | 7 | - |
| CmGSTU5 | GATA-motif | AAGGATAAGG | 358 | 9 | + |
| CmGSTU5 | TCA-element | TCAGAAGAGG | 259 | 9 | - |
| CmGSTU5 | ARE | AAACCA | 620 | 6 | - |
| CmGSTU5 | WRE3 | CCACCT | 506 | 6 | - |
| CmGSTU5 | Sp1 | GGGCGG | 560 | 6 | + |
| CmGSTU5 |  | motif_sequence | 100 | 4 | - |
| CmGSTU5 |  | motif_sequence | 117 | 4 | + |
| CmGSTU5 |  | motif_sequence | 122 | 4 | + |
| CmGSTU5 |  | motif_sequence | 153 | 4 | - |
| CmGSTU5 |  | motif_sequence | 187 | 4 | + |
| CmGSTU5 |  | motif_sequence | 213 | 4 | - |
| CmGSTU5 |  | motif_sequence | 260 | 4 | + |
| CmGSTU5 |  | motif_sequence | 502 | 4 | - |
| CmGSTU5 |  | motif_sequence | 599 | 4 | + |
| CmGSTU6 | Sp1 | GGGCGG | 311 | 6 | + |
| CmGSTU6 | Sp1 | GGGCGG | 339 | 6 | + |
| CmGSTU6 |  | motif_sequence | 47 | 4 | - |
| CmGSTU6 |  | motif_sequence | 64 | 4 | + |
| CmGSTU6 |  | motif_sequence | 257 | 4 | + |
| CmGSTU6 |  | motif_sequence | 328 | 4 | + |
| CmGSTU6 |  | motif_sequence | 394 | 4 | - |
| CmGSTU6 |  | motif_sequence | 430 | 4 | - |
| CmGSTU6 |  | motif_sequence | 435 | 4 | - |
| CmGSTU6 |  | motif_sequence | 472 | 4 | - |
| CmGSTU6 |  | motif_sequence | 505 | 4 | - |
| CmGSTU6 |  | motif_sequence | 554 | 4 | - |
| CmGSTU6 | dOCT | CACGGATC | 594 | 8 | + |
| CmGSTU6 | GT1-motif | GGTTAA | 29 | 6 | + |
| CmGSTU6 | O2-site | GATGA(C/T)(A/G)TG(A/G) | 4 | 8 | + |
| CmGSTU6 | TCCC-motif | TCTCCCT | 161 | 7 | + |
| CmGSTU6 | as-1 | TGACG | 219 | 5 | + |
| CmGSTU6 | TCA-element | TCAGAAGAGG | 391 | 9 | + |
| CmGSTU6 | CGTCA-motif | CGTCA | 219 | 5 | - |
| CmGSTU6 | G-box | CACGTC | 225 | 6 | - |
| CmGSTU6 | MYB-like sequence | TAACCA | 28 | 6 | - |
| CmGSTU6 | CCAAT-box | CAACGG | 740 | 6 | - |
| CmGSTU6 | Unnamed__4 | CTCC | 16 | 4 | + |
| CmGSTU6 | Unnamed__4 | CTCC | 122 | 4 | + |
| CmGSTU6 | Unnamed__4 | CTCC | 144 | 4 | + |
| CmGSTU6 | Unnamed__4 | CTCC | 162 | 4 | + |
| CmGSTU6 | Unnamed__4 | CTCC | 195 | 4 | + |
| CmGSTU6 | Unnamed__4 | CTCC | 247 | 4 | + |
| CmGSTU6 | Unnamed__4 | CTCC | 251 | 4 | + |
| CmGSTU6 | Unnamed__4 | CTCC | 259 | 4 | + |
| CmGSTU6 | Unnamed__4 | CTCC | 363 | 4 | - |
| CmGSTU6 | Unnamed__4 | CTCC | 430 | 4 | - |
| CmGSTU6 | Unnamed__4 | CTCC | 484 | 4 | - |
| CmGSTU6 | Unnamed__4 | CTCC | 505 | 4 | - |
| CmGSTU6 | Unnamed__4 | CTCC | 551 | 4 | - |
| CmGSTU6 | Unnamed__4 | CTCC | 615 | 4 | - |
| CmGSTU6 | Unnamed__4 | CTCC | 705 | 4 | - |
| CmGSTU6 | STRE | AGGGG | 240 | 5 | - |
| CmGSTU6 | STRE | AGGGG | 308 | 5 | + |
| CmGSTU6 | STRE | AGGGG | 354 | 5 | + |
| CmGSTU6 | TATA-box | TATA | 318 | 4 | + |
| CmGSTU6 | MYC | CATTTG | 54 | 6 | + |
| CmGSTU6 | MYB recognition site | CCGTTG | 740 | 6 | + |
| CmGSTU6 | MYB | TAACCA | 28 | 6 | - |
| CmGSTU6 | MYB | CAACCA | 478 | 6 | - |
| CmGSTU6 | ABRE | GACACGTGGC | 223 | 9 | + |
| CmGSTU6 | ABRE | ACGTG | 226 | 5 | + |
| CmGSTU6 | CAAT-box | CCAAT | 26 | 5 | - |
| CmGSTU6 | CAAT-box | CAAAT | 55 | 5 | - |
| CmGSTU6 | CAAT-box | CCAAT | 87 | 5 | + |
| CmGSTU6 | CAAT-box | CAAT | 88 | 4 | + |
| CmGSTU6 | CAAT-box | CAAT | 217 | 4 | - |
| CmGSTU6 | CAAT-box | CAAT | 298 | 4 | - |
| CmGSTU6 | CAAT-box | CAAT | 466 | 4 | + |
| CmGSTU6 | CAAT-box | CAAAT | 468 | 5 | - |
| CmGSTU6 | CAAT-box | CAAT | 513 | 4 | - |
| CmGSTU6 | CAAT-box | CCAAT | 674 | 5 | + |
| CmGSTU6 | CAAT-box | CAAT | 675 | 4 | + |
| CmGSTU6 | Unnamed__1 | CGTGG | 174 | 5 | - |
| CmGSTU6 | Unnamed__1 | CGTGG | 227 | 5 | + |
| CmGSTU6 | TGACG-motif | TGACG | 219 | 5 | + |
| CmGSTU7 | Myb | CAACTG | 389 | 6 | + |
| CmGSTU7 | MBS | CAACTG | 389 | 6 | + |
| CmGSTU7 | W box | TTGACC | 34 | 6 | + |
| CmGSTU7 | Unnamed__4 | CTCC | 55 | 4 | + |
| CmGSTU7 | Unnamed__4 | CTCC | 222 | 4 | - |
| CmGSTU7 | Unnamed__4 | CTCC | 304 | 4 | - |
| CmGSTU7 | Unnamed__4 | CTCC | 362 | 4 | + |
| CmGSTU7 | Unnamed__4 | CTCC | 368 | 4 | + |
| CmGSTU7 | CCAAT-box | CAACGG | 373 | 6 | - |
| CmGSTU7 | I-box | ccttatcct | 215 | 9 | - |
| CmGSTU7 | I-box | gGATAAGGTG | 216 | 9 | + |
| CmGSTU7 | MYB | CAACAG | 42 | 6 | + |
| CmGSTU7 | MYB | CAACAG | 159 | 6 | - |
| CmGSTU7 | STRE | AGGGG | 70 | 5 | - |
| CmGSTU7 | TATA-box | TATA | 129 | 4 | + |
| CmGSTU7 | MYC | CATTTG | 353 | 6 | - |
| CmGSTU7 | MYB recognition site | CCGTTG | 373 | 6 | + |
| CmGSTU7 | TGACG-motif | TGACG | 116 | 5 | - |
| CmGSTU7 | CAAT-box | CAAT | 22 | 4 | - |
| CmGSTU7 | CAAT-box | CAAAT | 32 | 5 | - |
| CmGSTU7 | CAAT-box | CAAT | 66 | 4 | + |
| CmGSTU7 | CAAT-box | CAAAT | 107 | 5 | + |
| CmGSTU7 | CAAT-box | CAAAT | 204 | 5 | + |
| CmGSTU7 | CAAT-box | CCAAT | 342 | 5 | + |
| CmGSTU7 | CAAT-box | CAAT | 343 | 4 | + |
| CmGSTU7 | CAAT-box | CAAAT | 353 | 5 | + |
| CmGSTU7 | CAAT-box | CAAT | 398 | 4 | + |
| CmGSTU7 | CAAT-box | CAAAT | 425 | 5 | + |
| CmGSTU7 |  | motif_sequence | 25 | 4 | - |
| CmGSTU7 |  | motif_sequence | 73 | 4 | + |
| CmGSTU7 |  | motif_sequence | 81 | 4 | - |
| CmGSTU7 |  | motif_sequence | 102 | 4 | + |
| CmGSTU7 |  | motif_sequence | 245 | 4 | - |
| CmGSTU7 |  | motif_sequence | 366 | 4 | + |
| CmGSTU7 |  | motif_sequence | 400 | 4 | + |
| CmGSTU7 | as-1 | TGACG | 116 | 5 | - |
| CmGSTU7 | Myb-binding site | CAACAG | 42 | 6 | + |
| CmGSTU7 | Myb-binding site | CAACAG | 159 | 6 | - |
| CmGSTU7 | GARE-motif | TCTGTTG | 158 | 7 | + |
| CmGSTU7 | GATA-motif | AAGGATAAGG | 214 | 9 | + |
| CmGSTU7 | CGTCA-motif | CGTCA | 116 | 5 | + |
| CmGSTU7 | LTR | CCGAAA | 86 | 6 | - |
| CmGSTU8 | CCGTCC-box | CCGTCC | 259 | 6 | + |
| CmGSTU8 | CCGTCC-box | CCGTCC | 531 | 6 | - |
| CmGSTU8 | Sp1 | GGGCGG | 563 | 6 | + |
| CmGSTU8 |  | motif_sequence | 131 | 4 | + |
| CmGSTU8 |  | motif_sequence | 162 | 4 | - |
| CmGSTU8 |  | motif_sequence | 196 | 4 | + |
| CmGSTU8 |  | motif_sequence | 222 | 4 | - |
| CmGSTU8 |  | motif_sequence | 251 | 4 | + |
| CmGSTU8 |  | motif_sequence | 349 | 4 | - |
| CmGSTU8 |  | motif_sequence | 355 | 4 | - |
| CmGSTU8 |  | motif_sequence | 374 | 4 | - |
| CmGSTU8 |  | motif_sequence | 406 | 4 | - |
| CmGSTU8 |  | motif_sequence | 442 | 4 | - |
| CmGSTU8 |  | motif_sequence | 447 | 4 | - |
| CmGSTU8 |  | motif_sequence | 522 | 4 | - |
| CmGSTU8 |  | motif_sequence | 632 | 4 | + |
| CmGSTU8 | O2-site | GATGACATGG | 109 | 9 | + |
| CmGSTU8 | TGA-element | AACGAC | 229 | 6 | + |
| CmGSTU8 | GATA-motif | GATAGGG | 91 | 7 | - |
| CmGSTU8 | GATA-motif | GATAGGG | 192 | 7 | - |
| CmGSTU8 | GATA-motif | AAGGATAAGG | 310 | 9 | + |
| CmGSTU8 | A-box | CCGTCC | 259 | 6 | + |
| CmGSTU8 | A-box | CCGTCC | 531 | 6 | - |
| CmGSTU8 | CCGTCC motif | CCGTCC | 259 | 6 | + |
| CmGSTU8 | CCGTCC motif | CCGTCC | 531 | 6 | - |
| CmGSTU8 | TCA | TCATCTTCAT | 520 | 9 | - |
| CmGSTU8 | CARE | CAACTCCC | 31 | 8 | - |
| CmGSTU8 | DRE core | GCCGAC | 644 | 6 | + |
| CmGSTU8 | Unnamed__4 | CTCC | 25 | 4 | + |
| CmGSTU8 | Unnamed__4 | CTCC | 32 | 4 | - |
| CmGSTU8 | Unnamed__4 | CTCC | 133 | 4 | + |
| CmGSTU8 | Unnamed__4 | CTCC | 136 | 4 | + |
| CmGSTU8 | Unnamed__4 | CTCC | 332 | 4 | - |
| CmGSTU8 | Unnamed__4 | CTCC | 349 | 4 | - |
| CmGSTU8 | Unnamed__4 | CTCC | 366 | 4 | - |
| CmGSTU8 | Unnamed__4 | CTCC | 374 | 4 | - |
| CmGSTU8 | Unnamed__4 | CTCC | 439 | 4 | - |
| CmGSTU8 | Unnamed__4 | CTCC | 442 | 4 | - |
| CmGSTU8 | Unnamed__4 | CTCC | 447 | 4 | - |
| CmGSTU8 | Unnamed__4 | CTCC | 553 | 4 | + |
| CmGSTU8 | Unnamed__4 | CTCC | 628 | 4 | + |
| CmGSTU8 | Unnamed__4 | CTCC | 634 | 4 | + |
| CmGSTU8 | Unnamed__4 | CTCC | 658 | 4 | + |
| CmGSTU8 | Box 4 | ATTAAT | 451 | 6 | - |
| CmGSTU8 | MYC | CATTTG | 619 | 6 | - |
| CmGSTU8 | TATA-box | TACATAAA | 223 | 8 | + |
| CmGSTU8 | I-box | ccttatcct | 311 | 9 | - |
| CmGSTU8 | MYB | CAACCA | 458 | 6 | - |
| CmGSTU8 | CAAT-box | CAAAT | 64 | 5 | - |
| CmGSTU8 | CAAT-box | CAAT | 147 | 4 | + |
| CmGSTU8 | CAAT-box | CAAAT | 300 | 5 | + |
| CmGSTU8 | CAAT-box | CAAAT | 464 | 5 | - |
| CmGSTU8 | CAAT-box | CAAAT | 619 | 5 | + |
| CmGSTU8 | ABRE | GACACGTGGC | 232 | 9 | + |
| CmGSTU9 | CAAT-box | CAAT | 97 | 4 | - |
| CmGSTU9 | CAAT-box | CAAAT | 136 | 5 | + |
| CmGSTU9 | CAAT-box | CCAAT | 145 | 5 | + |
| CmGSTU9 | CAAT-box | CAAT | 146 | 4 | + |
| CmGSTU9 | CAAT-box | CAAT | 177 | 4 | + |
| CmGSTU9 | CAAT-box | CAAAT | 190 | 5 | - |
| CmGSTU9 | CAAT-box | CAAT | 208 | 4 | - |
| CmGSTU9 | CAAT-box | CAAT | 220 | 4 | - |
| CmGSTU9 | CAAT-box | CAAT | 304 | 4 | - |
| CmGSTU9 | CAAT-box | CAAT | 393 | 4 | - |
| CmGSTU9 | CAAT-box | CAAT | 400 | 4 | + |
| CmGSTU9 | CAAT-box | CCAAT | 402 | 5 | - |
| CmGSTU9 | CAAT-box | CAAT | 463 | 4 | - |
| CmGSTU9 | CAAT-box | CCAAT | 472 | 5 | - |
| CmGSTU9 | CAAT-box | CAAT | 506 | 4 | + |
| CmGSTU9 | CAAT-box | CAAT | 523 | 4 | - |
| CmGSTU9 | CAAT-box | CAAAT | 655 | 5 | - |
| CmGSTU9 | TATA-box | TATA | 94 | 4 | + |
| CmGSTU9 | TATA-box | TATA | 217 | 4 | + |
| CmGSTU9 | TATA-box | ATTATA | 454 | 6 | + |
| CmGSTU9 | TATA-box | TATAA | 455 | 5 | - |
| CmGSTU9 | TATA-box | TATA | 456 | 4 | - |
| CmGSTU9 | TATA-box | TATA | 493 | 4 | - |
| CmGSTU9 | MYC | CATTTG | 136 | 6 | - |
| CmGSTU9 | MYC | CATTTG | 189 | 6 | + |
| CmGSTU9 | MYC | CAATTG | 400 | 6 | - |
| CmGSTU9 | AE-box | AGAAACAA | 112 | 8 | + |
| CmGSTU9 | TCCC-motif | TCTCCCT | 350 | 7 | - |
| CmGSTU9 | Unnamed__4 | CTCC | 245 | 4 | + |
| CmGSTU9 | Unnamed__4 | CTCC | 352 | 4 | - |
| CmGSTU9 | Unnamed__4 | CTCC | 378 | 4 | - |
| CmGSTU9 | Unnamed__4 | CTCC | 418 | 4 | - |
| CmGSTU9 | Unnamed__4 | CTCC | 436 | 4 | - |
| CmGSTU9 | Unnamed__4 | CTCC | 603 | 4 | + |
| CmGSTU9 | AAGAA-motif | GAAAGAA | 371 | 7 | + |
| CmGSTU9 |  | motif_sequence | 4 | 4 | - |
| CmGSTU9 |  | motif_sequence | 70 | 4 | - |
| CmGSTU9 |  | motif_sequence | 103 | 4 | - |
| CmGSTU9 |  | motif_sequence | 198 | 4 | + |
| CmGSTU9 |  | motif_sequence | 333 | 4 | - |
| CmGSTU9 |  | motif_sequence | 355 | 4 | - |
| CmGSTU9 |  | motif_sequence | 378 | 4 | - |
| CmGSTU9 | Unnamed_1 | GGATTTTACAGT | 475 | 11 | + |
| CmGSTZ1 | AAGAA-motif | GAAAGAA | 69 | 7 | - |
| CmGSTZ1 | TCA | TCATCTTCAT | 330 | 9 | + |
| CmGSTZ1 | DRE core | GCCGAC | 303 | 6 | + |
| CmGSTZ1 | TC-rich repeats | GTTTTCTTAC | 126 | 9 | + |
| CmGSTZ1 | Unnamed__4 | CTCC | 30 | 4 | + |
| CmGSTZ1 | Unnamed__4 | CTCC | 80 | 4 | - |
| CmGSTZ1 | Unnamed__4 | CTCC | 84 | 4 | + |
| CmGSTZ1 | Unnamed__4 | CTCC | 158 | 4 | - |
| CmGSTZ1 | Unnamed__4 | CTCC | 514 | 4 | - |
| CmGSTZ1 | Unnamed__4 | CTCC | 548 | 4 | + |
| CmGSTZ1 | TATA-box | TATA | 348 | 4 | - |
| CmGSTZ1 | TGACG-motif | TGACG | 41 | 5 | - |
| CmGSTZ1 | Unnamed__1 | CGTGG | 92 | 5 | + |
| CmGSTZ1 | Unnamed__1 | CGTGG | 234 | 5 | + |
| CmGSTZ1 | CAAT-box | CCAAT | 9 | 5 | + |
| CmGSTZ1 | CAAT-box | CAAT | 10 | 4 | + |
| CmGSTZ1 | CAAT-box | CAAT | 169 | 4 | + |
| CmGSTZ1 | CAAT-box | CAAT | 387 | 4 | - |
| CmGSTZ1 | CAAT-box | CAAAT | 411 | 5 | - |
| CmGSTZ1 | CAAT-box | CAAT | 442 | 4 | + |
| CmGSTZ1 | CAAT-box | CAAT | 465 | 4 | + |
| CmGSTZ1 | CAAT-box | CCAAT | 501 | 5 | + |
| CmGSTZ1 | CAAT-box | CAAT | 502 | 4 | + |
| CmGSTZ1 | CAAT-box | CAAAT | 553 | 5 | + |
| CmGSTZ1 | CAAT-box | CAAT | 568 | 4 | - |
| CmGSTZ1 | CAAT-box | CAAT | 583 | 4 | - |
| CmGSTZ1 |  | motif_sequence | 138 | 4 | - |
| CmGSTZ1 |  | motif_sequence | 161 | 4 | - |
| CmGSTZ1 |  | motif_sequence | 223 | 4 | - |
| CmGSTZ1 |  | motif_sequence | 274 | 4 | - |
| CmGSTZ1 |  | motif_sequence | 311 | 4 | + |
| CmGSTZ1 |  | motif_sequence | 326 | 4 | + |
| CmGSTZ1 |  | motif_sequence | 332 | 4 | + |
| CmGSTZ1 |  | motif_sequence | 421 | 4 | - |
| CmGSTZ1 |  | motif_sequence | 514 | 4 | - |
| CmGSTZ1 | as-1 | TGACG | 41 | 5 | - |
| CmGSTZ1 | TCT-motif | TCTTAC | 130 | 6 | + |
| CmGSTZ1 | TCT-motif | TCTTAC | 625 | 6 | + |
| CmGSTZ1 | ARE | AAACCA | 395 | 6 | + |
| CmGSTZ1 | CAT-box | GCCACT | 339 | 6 | - |
| CmGSTZ1 | ERE | ATTTTAAA | 318 | 8 | - |
| CmGSTZ1 | CGTCA-motif | CGTCA | 41 | 5 | + |
| CmGSTZ2 | G-Box | CACGTT | 62 | 6 | - |
| CmGSTZ2 | Myb-binding site | CAACAG | 113 | 6 | - |
| CmGSTZ2 | Myb-binding site | CAACAG | 167 | 6 | - |
| CmGSTZ2 | TCCC-motif | TCTCCCT | 128 | 7 | - |
| CmGSTZ2 |  | motif_sequence | 18 | 4 | + |
| CmGSTZ2 |  | motif_sequence | 31 | 4 | + |
| CmGSTZ2 |  | motif_sequence | 130 | 4 | - |
| CmGSTZ2 |  | motif_sequence | 338 | 4 | + |
| CmGSTZ2 |  | motif_sequence | 556 | 4 | + |
| CmGSTZ2 |  | motif_sequence | 600 | 4 | - |
| CmGSTZ2 |  | motif_sequence | 616 | 4 | - |
| CmGSTZ2 | ARE | AAACCA | 171 | 6 | - |
| CmGSTZ2 | ARE | AAACCA | 633 | 6 | + |
| CmGSTZ2 | AE-box | AGAAACAA | 317 | 8 | - |
| CmGSTZ2 | AE-box | AGAAACTT | 392 | 8 | + |
| CmGSTZ2 | CCAAT-box | CAACGG | 22 | 6 | - |
| CmGSTZ2 | Unnamed__4 | CTCC | 20 | 4 | + |
| CmGSTZ2 | Unnamed__4 | CTCC | 130 | 4 | - |
| CmGSTZ2 | Unnamed__4 | CTCC | 143 | 4 | + |
| CmGSTZ2 | Unnamed__4 | CTCC | 240 | 4 | - |
| CmGSTZ2 | Unnamed__4 | CTCC | 390 | 4 | - |
| CmGSTZ2 | Unnamed__4 | CTCC | 481 | 4 | - |
| CmGSTZ2 | Unnamed__4 | CTCC | 647 | 4 | + |
| CmGSTZ2 | TCA | TCATCTTCAT | 519 | 9 | + |
| CmGSTZ2 | MBS | CAACTG | 455 | 6 | - |
| CmGSTZ2 | Myb | CAACTG | 455 | 6 | - |
| CmGSTZ2 | CAAT-box | CAAT | 73 | 4 | - |
| CmGSTZ2 | CAAT-box | CAAT | 123 | 4 | - |
| CmGSTZ2 | CAAT-box | CAAAT | 137 | 5 | - |
| CmGSTZ2 | CAAT-box | CAAT | 162 | 4 | + |
| CmGSTZ2 | CAAT-box | CAAT | 208 | 4 | - |
| CmGSTZ2 | CAAT-box | CAAAT | 236 | 5 | - |
| CmGSTZ2 | CAAT-box | CAAAT | 311 | 5 | + |
| CmGSTZ2 | CAAT-box | CAAT | 316 | 4 | - |
| CmGSTZ2 | CAAT-box | CAAT | 352 | 4 | - |
| CmGSTZ2 | CAAT-box | CCAAT | 418 | 5 | - |
| CmGSTZ2 | CAAT-box | CAAAT | 471 | 5 | - |
| CmGSTZ2 | CAAT-box | CAAAT | 487 | 5 | + |
| CmGSTZ2 | CAAT-box | CAAT | 535 | 4 | - |
| CmGSTZ2 | CAAT-box | CAAT | 546 | 4 | + |
| CmGSTZ2 | CAAT-box | CCAAT | 568 | 5 | + |
| CmGSTZ2 | CAAT-box | CAAT | 569 | 4 | + |
| CmGSTZ2 | CAAT-box | CAAAT | 612 | 5 | - |
| CmGSTZ2 | ABRE | ACGTG | 63 | 5 | + |
| CmGSTZ2 | MYC | CATTTG | 136 | 6 | + |
| CmGSTZ2 | MYC | CATGTG | 409 | 6 | - |
| CmGSTZ2 | MYC | CATTTG | 611 | 6 | + |
| CmGSTZ2 | Box 4 | ATTAAT | 295 | 6 | + |
| CmGSTZ2 | MYB recognition site | CCGTTG | 22 | 6 | + |
| CmGSTZ2 | TATA-box | TACAAAA | 174 | 7 | - |
| CmGSTZ2 | TATA-box | TATA | 225 | 4 | + |
| CmGSTZ2 | MYB | CAACAG | 113 | 6 | - |
| CmGSTZ2 | MYB | CAACAG | 167 | 6 | - |
| CmGSTZ2 | MRE | AACCTAA | 82 | 7 | + |
| CmGSTZ3 | CGTCA-motif | CGTCA | 645 | 5 | - |
| CmGSTZ3 | TCT-motif | TCTTAC | 40 | 6 | + |
| CmGSTZ3 | TCCC-motif | TCTCCCT | 131 | 7 | - |
| CmGSTZ3 | Myb-binding site | CAACAG | 268 | 6 | - |
| CmGSTZ3 | as-1 | TGACG | 645 | 5 | + |
| CmGSTZ3 | GARE-motif | TCTGTTG | 267 | 7 | + |
| CmGSTZ3 |  | motif_sequence | 122 | 4 | + |
| CmGSTZ3 |  | motif_sequence | 133 | 4 | - |
| CmGSTZ3 |  | motif_sequence | 144 | 4 | + |
| CmGSTZ3 |  | motif_sequence | 179 | 4 | + |
| CmGSTZ3 |  | motif_sequence | 341 | 4 | + |
| CmGSTZ3 |  | motif_sequence | 356 | 4 | + |
| CmGSTZ3 |  | motif_sequence | 366 | 4 | - |
| CmGSTZ3 |  | motif_sequence | 484 | 4 | - |
| CmGSTZ3 |  | motif_sequence | 559 | 4 | + |
| CmGSTZ3 |  | motif_sequence | 660 | 4 | + |
| CmGSTZ3 | TGACG-motif | TGACG | 645 | 5 | + |
| CmGSTZ3 | CAAT-box | CAAT | 76 | 4 | - |
| CmGSTZ3 | CAAT-box | CAAT | 139 | 4 | + |
| CmGSTZ3 | CAAT-box | CCAAT | 172 | 5 | - |
| CmGSTZ3 | CAAT-box | CAAAT | 239 | 5 | - |
| CmGSTZ3 | CAAT-box | CAAAT | 314 | 5 | + |
| CmGSTZ3 | CAAT-box | CAAT | 370 | 4 | - |
| CmGSTZ3 | CAAT-box | CAAT | 421 | 4 | - |
| CmGSTZ3 | CAAT-box | CAAAT | 523 | 5 | + |
| CmGSTZ3 | CAAT-box | CAAT | 540 | 4 | + |
| CmGSTZ3 | CAAT-box | CAAT | 549 | 4 | + |
| CmGSTZ3 | CAAT-box | CAAT | 553 | 4 | - |
| CmGSTZ3 | CAAT-box | CAAAT | 564 | 5 | + |
| CmGSTZ3 | CAAT-box | CAAT | 600 | 4 | + |
| CmGSTZ3 | MYB | TAACCA | 174 | 6 | - |
| CmGSTZ3 | MYB | CAACAG | 268 | 6 | - |
| CmGSTZ3 | TATA-box | TATA | 228 | 4 | + |
| CmGSTZ3 | TATA-box | TATA | 606 | 4 | - |
| CmGSTZ3 | Box 4 | ATTAAT | 298 | 6 | + |
| CmGSTZ3 | Unnamed__4 | CTCC | 47 | 4 | - |
| CmGSTZ3 | Unnamed__4 | CTCC | 50 | 4 | - |
| CmGSTZ3 | Unnamed__4 | CTCC | 133 | 4 | - |
| CmGSTZ3 | Unnamed__4 | CTCC | 146 | 4 | + |
| CmGSTZ3 | Unnamed__4 | CTCC | 243 | 4 | - |
| CmGSTZ3 | Unnamed__4 | CTCC | 246 | 4 | - |
| CmGSTZ3 | Unnamed__4 | CTCC | 484 | 4 | - |
| CmGSTZ3 | Unnamed__4 | CTCC | 518 | 4 | + |
| CmGSTZ3 | Unnamed__4 | CTCC | 653 | 4 | + |
| CmGSTZ3 | DRE core | GCCGAC | 214 | 6 | + |
| CmGSTZ3 | MYB-like sequence | TAACCA | 174 | 6 | - |
| CmTCHQD1 | TCT-motif | TCTTAC | 550 | 6 | + |
| CmTCHQD1 | AT~TATA-box | TATATA | 211 | 6 | + |
| CmTCHQD1 | AT~TATA-box | TATATA | 640 | 6 | - |
| CmTCHQD1 | CGTCA-motif | CGTCA | 519 | 5 | - |
| CmTCHQD1 | LTR | CCGAAA | 407 | 6 | + |
| CmTCHQD1 |  | motif_sequence | 58 | 4 | - |
| CmTCHQD1 |  | motif_sequence | 111 | 4 | - |
| CmTCHQD1 |  | motif_sequence | 120 | 4 | + |
| CmTCHQD1 |  | motif_sequence | 565 | 4 | - |
| CmTCHQD1 |  | motif_sequence | 631 | 4 | - |
| CmTCHQD1 |  | motif_sequence | 664 | 4 | - |
| CmTCHQD1 |  | motif_sequence | 674 | 4 | - |
| CmTCHQD1 |  | motif_sequence | 687 | 4 | - |
| CmTCHQD1 |  | motif_sequence | 794 | 4 | - |
| CmTCHQD1 | Myb-binding site | CAACAG | 479 | 6 | - |
| CmTCHQD1 | as-1 | TGACG | 519 | 5 | + |
| CmTCHQD1 | GARE-motif | TCTGTTG | 478 | 7 | + |
| CmTCHQD1 | MYB | CAACAG | 479 | 6 | - |
| CmTCHQD1 | TATA-box | TATAA | 7 | 5 | - |
| CmTCHQD1 | TATA-box | TATA | 8 | 4 | + |
| CmTCHQD1 | TATA-box | TATATA | 211 | 6 | + |
| CmTCHQD1 | TATA-box | TATA | 213 | 4 | + |
| CmTCHQD1 | TATA-box | TATAAAT | 366 | 7 | - |
| CmTCHQD1 | TATA-box | TATAAA | 367 | 6 | - |
| CmTCHQD1 | TATA-box | TATAA | 368 | 5 | - |
| CmTCHQD1 | TATA-box | TATA | 369 | 4 | + |
| CmTCHQD1 | TATA-box | TATATA | 640 | 6 | - |
| CmTCHQD1 | TATA-box | TATA | 642 | 4 | - |
| CmTCHQD1 | STRE | AGGGG | 561 | 5 | + |
| CmTCHQD1 | MYC | CAATTG | 194 | 6 | + |
| CmTCHQD1 | MYC | CAATTG | 227 | 6 | + |
| CmTCHQD1 | TGACG-motif | TGACG | 519 | 5 | + |
| CmTCHQD1 | CAAT-box | CAAT | 70 | 4 | - |
| CmTCHQD1 | CAAT-box | CAAT | 93 | 4 | + |
| CmTCHQD1 | CAAT-box | CAAT | 194 | 4 | + |
| CmTCHQD1 | CAAT-box | CAAT | 196 | 4 | - |
| CmTCHQD1 | CAAT-box | CAAT | 223 | 4 | - |
| CmTCHQD1 | CAAT-box | CAAT | 227 | 4 | + |
| CmTCHQD1 | CAAT-box | CAAT | 229 | 4 | - |
| CmTCHQD1 | CAAT-box | CAAT | 301 | 4 | + |
| CmTCHQD1 | CAAT-box | CAAAT | 462 | 5 | + |
| CmTCHQD1 | CAAT-box | CAAT | 465 | 4 | - |
| CmTCHQD1 | CAAT-box | CAAAT | 474 | 5 | + |
| CmTCHQD1 | CAAT-box | CAAT | 621 | 4 | - |
| CmTCHQD1 | CAAT-box | CAAT | 661 | 4 | - |
| CmTCHQD1 | CAAT-box | CCAAT | 671 | 5 | - |
| CmTCHQD1 | CAAT-box | CCAAT | 678 | 5 | - |
| CmTCHQD1 | CAAT-box | CCAAT | 712 | 5 | - |
| CmTCHQD1 | CAAT-box | CAAT | 800 | 4 | - |
| CmTCHQD1 | Myb | TAACTG | 4 | 6 | - |
| CmTCHQD1 | Unnamed__4 | CTCC | 234 | 4 | + |
| CmTCHQD1 | Unnamed__4 | CTCC | 404 | 4 | + |
| CmTCHQD1 | Unnamed__4 | CTCC | 734 | 4 | - |
| CmTCHQD1 | Unnamed__4 | CTCC | 751 | 4 | + |

**Table S7**

The sequences of specific primers used for q-PCR analysis

| **ID** | **Description** | **Forward primer (5’3’)** | **Reverse primer (5’3’)** |
| --- | --- | --- | --- |
| LOC103484230 | GAPDH | AAAGACTGGAGAGGTGGAAGAGC | TCAACGGTAGGAACACGGAAAGA |
| CmGSTU3 | MELO3C006221T1 | CCTTCACATCAAGCCAAGACAA | GCCATCCAACTACAATATCCACAA |
| CmGSTU4 | MELO3C006352T1 | GAAGATTCCAGTCCTCGTCCAT | ACGGTTCTCAGCATCTCTAAGC |
| CmGSTU5 | MELO3C006353T1 | AAGTGTTGAAGACGGTGGAAGAA | CCAAGCATACAGCCGAGGAA |
| CmGSTU7 | MELO3C006356T1 | GGAAGAGCGAGAGCGAGAG | GGCAGATTGAAGCAGTTGTTGT |
| CmGSTU10 | MELO3C016167T1 | AGGAAGGGAAGAAGGAGGTGATA | GCAAGGACTTAGCCACACTCT |
| CmGSTU11 | MELO3C016168T1 | TGTGAATCTTCCATAATCGTCCAGT | CCTTCTTCTTGCTCCTCTCCTT |
| CmGSTU12 | MELO3C016169T1 | TGTGGCGTATGAGTATGTGGAA | ATCGGACGGCAACAGAGG |
| CmGSTU14 | MELO3C003190T1 | TCAGGTTCGTATAGTCGGAGAGT | GGCACTTTCTTGTAAATTGGGTTGT |
| CmGSTU15 | MELO3C003192T1 | CACAGGCGGCACTTGAAC | GCTTCTTCGGTGGCAGGA |
| CmGSTU16 | MELO3C003194T1 | ACAGAGGAGACATTAGAACCACTTG | GCTTCTTCAACGGCTGGAATC |
| CmGSTU18 | MELO3C003201T3 | AAGGTTGTGGCTGCTATACTGA | GCTTCTTCAATGGCTGGAATCC |
| CmGSTU19 | MELO3C025097T1 | CTTCTTCCTTCCTCGCCCTAC | TTGCTCTTCTCCGTTGCTTCT |
| CmGSTU20 | MELO3C012092T1 | GGTGCCTTGCCTATCCTCAA | TACATCTCCAACAACCTCAAACAGT |
| CmGSTU22 | MELO3C001977T1 | ATCAAAGTAACTCCACACAGACAAG | CACCAGATTCTTCTCCTCTACCAT |
| CmGSTU23 | MELO3C001175T1 | GGAAGCACAAGAGAAAGCCATT | TATCCAAGTTCGTCTCCATTCACAA |
| CmGSTF1 | MELO3C016031T1 | GGCTGGTGCTATCAAGGTTCA | CGCTTGTGTTCTCCTTCGTGTA |
| CmGSTF2 | MELO3C016032T1 | GCACGAAGGCGAGCATAAG | ATGATGACTCTCCACTTCAATCCA |
| CmGSTF3 | MELO3C016033T1 | AGCGGTGGCAGAGAATGAA | CAGCTTCTTCGACGGTGTTG |
| CmGSTF4 | MELO3C016034T1 | CCAGAGAAGAAGCCATTGTGAAG | CGAAGGTGAGCAGTGAAGAAC |
| CmGSTF5 | MELO3C011334T1 | ACCGCCGTCTCCAGAGTA | TGATCCAGTCCGTACAGTCCTT |
| CmGSTL1 | MELO3C009300T3 | AGTATGCTGAAGAGTTGCTATCCT | ATGTATGCTATATCCACCAGACTGA |
| CmGSTL2 | MELO3C009299T2 | CCGAACAACCTCCGCTCTT | TTCCAACGCTGGCACCTT |
| CmGSTL3 | MELO3C011714T2 | CCACTCTTCGTCTTCCTCAGAT | GATATGTAAAGCCTCGTTGTTCCA |
| CmGSTT1 | MELO3C016349T2 | TTGAGGAGGTGAAGGTAGCC | ATGATGCCAATCCAATACTGAATGT |
| CmGSTZ1 | MELO3C006189T1 | GCTATTGAGAACTTCGGCATTGAC | CTGGCGATGAGGCTTGGA |
| CmGSTZ2 | MELO3C023220T1 | TGGCAGTGGACGAAGAATCT | TGCTCTCCCTTCAATAAATCAACAG |
| CmTCHQD1 | MELO3C010516T3 | ACCGACGACATAATGCCAAGTA | ATGCGAGCGATCACCACT |
| CmDHAR2 | MELO3C023826T2 | GCCTTCTCTTGTTACTCCTCCT | AACTTCGGTGCCAGACTCA |

**Table S8**

KEGG enrichment analysis of 28 stress-related CmGSTs.

| **Description** | **Count** | **Gene ratio** | ***P* value** |
| --- | --- | --- | --- |
| Glutathione metabolism | 15 | 81.34920635 | 0 |
| Metabolism of other amino acids | 15 | 35.77661431 | 0 |
| Transporters | 15 | 15.89147287 | 0 |
| Protein families: signaling and cellular processes | 15 | 5.800792303 | 3.38E-10 |
| Metabolism | 17 | 3.37742889 | 1.19E-08 |

**Table S9**

The list of identified glutathione biosynthesis-related amino acids in JS and GE at different storage periods.

| **Name** | **Species** | **0_d** | **6_d** | **12_d** | **18_d** | **24_d** |
| --- | --- | --- | --- | --- | --- | --- |
| L-glutamic_acid | JS_1 | 99494.422 | 56768.676 | 167184.696 | 242828.94 | 155440 |
|  | JS_2 | 62143.046 | 126536.507 | 158065.428 | 182838.66 | 81161.17 |
|  | JS_3 | 80836.72 | 109635.61 | 182635.2 | 210056.98 | 129863.6 |
|  | JS_4 | 98663.21 | 89745.36 | 202301.51 | 198743.55 | 105331.1 |
|  | JS_5 | 59596.98 | 98756.78 | 142376.22 | 205563.51 | 92037.69 |
|  | JS_6 | 84178.026 | 68472.616 | 123187.318 | 236971.1 | 145970 |
|  | GE_1 | 519854.417 | 683465.21 | 424509.187 | 474726.39 | 258428.7 |
|  | GE_2 | 153150.721 | 328767.622 | 434733.645 | 319768.37 | 243113.1 |
|  | GE_3 | 497563.021 | 435913.21 | 389965.214 | 368774.21 | 267859.6 |
|  | GE_4 | 358994.127 | 624558.312 | 456339.18 | 431229.23 | 281335.2 |
|  | GE_5 | 236497.177 | 532117.302 | 402559.369 | 396587.53 | 220635.6 |
|  | GE_6 | 252955.951 | 431876.84 | 469621.901 | 392398.53 | 233253.2 |
| gamma-glutamylcysteine | JS_1 | 1906401.57 | 2094655.92 | 3968720.46 | 1570277.5 | 485120.6 |
|  | JS_2 | 455912.195 | 943572.267 | 2028824.8 | 1317794.3 | 721006.8 |
|  | JS_3 | 936978.63 | 1159794.34 | 3633785.66 | 1489035.3 | 542398.3 |
|  | JS_4 | 1563874.32 | 1863434.01 | 3098779.27 | 1603215.3 | 658997.3 |
|  | JS_5 | 1355896.15 | 1667856.12 | 3422368.2 | 1387123.5 | 587763.2 |
|  | JS_6 | 867878.43 | 1385371.9 | 1840157.39 | 1296769.6 | 623095.9 |
|  | GE_1 | 13865491.8 | 17286114 | 1712245.57 | 5216484.8 | 1108748 |
|  | GE_2 | 531202.862 | 5154212.46 | 5299606.05 | 2035833.4 | 621067.8 |
|  | GE_3 | 6593321.21 | 12566945.2 | 3025498.25 | 3014879.5 | 985701.2 |
|  | GE_4 | 7856473.49 | 15820136.2 | 2879149.57 | 3956721.3 | 857717.2 |
|  | GE_5 | 11532479.2 | 9036548.35 | 3825143.2 | 4133578 | 775983 |
|  | GE_6 | 2811115.47 | 7457023.14 | 4293912.22 | 3399457.5 | 840229.8 |
